# Associations between aspirin use and the risk of cancers: a meta-analysis of observational studies

**DOI:** 10.1186/s12885-018-4156-5

**Published:** 2018-03-13

**Authors:** Yan Qiao, Tingting Yang, Yong Gan, Wenzhen Li, Chao Wang, Yanhong Gong, Zuxun Lu

**Affiliations:** 1 Department of Social Medicine and Health Management, School of Public Health, Tongji Medical College, Huazhong University of Science and Technology, Wuhan, 430030 People’s Republic of China; 2 Department of Nutriology, The People’s Hospital of Henan Province, Zhengzhou, Henan 450003 People’s Republic of China

**Keywords:** Aspirin, Cancers, Meta-analysis, Observational studies

## Abstract

**Background:**

Epidemiological studies have clarified the potential associations between regular aspirin use and cancers. However, it remains controversial on whether aspirin use decreases the risk of cancers risks. Therefore, we conducted an updated meta-analysis to assess the associations between aspirin use and cancers.

**Methods:**

The PubMed, Embase, and Web of Science databases were systematically searched up to March 2017 to identify relevant studies. Relative risks (RRs) with 95% confidence intervals (CIs) were used to assess the strength of associations.

**Results:**

A total of 218 studies with 309 reports were eligible for this meta-analysis. Aspirin use was associated with a significant decrease in the risk of overall cancer (RR = 0.89, 95% CI: 0.87–0.91), and gastric (RR = 0.75, 95% CI: 0.65–0.86), esophageal (RR = 0.75, 95% CI: 0.62–0.89), colorectal (RR = 0.79, 95% CI: 0.74–0.85), pancreatic (RR = 0.80, 95% CI: 0.68–0.93), ovarian (RR = 0.89, 95% CI: 0.83–0.95), endometrial (RR = 0.92, 95% CI: 0.85–0.99), breast (RR = 0.92, 95% CI: 0.88–0.96), and prostate (RR = 0.94, 95% CI: 0.90–0.99) cancers, as well as small intestine neuroendocrine tumors (RR = 0.17, 95% CI: 0.05–0.58).

**Conclusions:**

These findings suggest that aspirin use is associated with a reduced risk of gastric, esophageal, colorectal, pancreatic, ovarian, endometrial, breast, and prostate cancers, and small intestine neuroendocrine tumors.

**Electronic supplementary material:**

The online version of this article (10.1186/s12885-018-4156-5) contains supplementary material, which is available to authorized users.

## Background

Aspirin has been used as an analgesic and in the prevention of cardiovascular diseases events in the past decades and is one of the most commonly used drugs worldwide [1, 2]. Clinical and epidemiological studies reported that the rates of aspirin usage in different populations across different countries ranging from 11% to 54% [3–5]. Since the 1970s, many researchers started to focus on the effects of aspirin on cancers [6, 7]. However, these original studies were not comprehensive, and the effects on some cancers were controversial [8, 9].

Although several meta-analyses have been conducted to assess the associations between aspirin use and the risk of cancers(e.g., gastric, esophageal, pancreatic, lung, squamous cell carcinoma, breast, ovarian, and prostate cancers) [10–18], most of these studies were restricted to certain types of cancers, and some types such as hepatobiliary and cervical cancer could not be investigated. In addition, 70 new studies have been published since 2012. Therefore, this comprehensive systematic review and updated meta-analysis was conducted to explore the reliability of risk estimates between aspirin usage and most types of cancers and provide a landscape of aspirin use and cancer incidence.

## Methods

### Search strategy

This systematic review was conducted in accordance with the checklist proposed by the Meta-analysis of Observational Studies in Epidemiology group [19]. We searched multiple electronic bibliographic databases to identify studies published from database inception till March 2017, including PubMed, Embase, and Web of Science databases, with the following search terms: (“cancer” OR “neoplasm” OR “carcinoma”) AND (“aspirin” OR “acetylsalicylic acid” OR “non-steroidal anti-inflammatory drugs” OR “NSAIDs”). We restricted our search to human studies and published in English. In addition, reference lists from relevant reviews and retrieved articles were searched for qualifying studies.

### Inclusion criteria

The inclusion criteria were: 1) case-control or cohort studies; 2) studies that evaluated the relationships between the use of aspirin and the risk of cancers; 3) studies that reported risk estimates with 95% confidence interval (CI) or provided information that enabled us to calculate them. The exclusion criteria were: 1) studies that used other combinations of NSAIDs, which prevented the determination of the specific effect of aspirin, and 2) studies involving patients with specific diseases (e.g., Barrett’s esophagus, Crohn’s disease, or ulcerative colitis). Only the latest or the most informative study was included when multiple studies were published on the same study population.

### Data extraction

The following information was obtained from each study: first author name, year of publication, study period, study location, study design, number of cases, number of participants, gender, definition of aspirin exposure, as curtained methods of exposure, odds ratios (ORs), hazard ratios (HRs) or relative risks (RRs) with their corresponding 95% CIs, and confounding factors adjusted in the analysis. The most fully-adjusted risk estimates with its corresponding 95% CIs (when available) were preferentially extracted. Data extraction was conducted independently by two authors (Y.Q. and T.T.Y.), and discrepancies were resolved by discussion with a third investigator (Z.X.L.).

### Quality assessment

Quality assessment of eligible studies was performed independently by two reviewers (Y.Q. and T.T.Y.) according to the Newcastle-Ottawa Quality Assessment Scale [20]. This scale allocates a maximum of nine points based on the selection (0–4 points), comparability (0–2 points), and exposure/outcome of the study participants (0–3 points). Scores of 0–3, 4–6, and 7–9 were classified as low, moderate, and high-quality studies respectively.

### Statistical analysis

RRs were used as the common measurement of the associations between aspirin use and the risk of cancer. Because cancer is a rare event in general, we could generally ignore the distinctions among the various measures of relative risk (e.g., odds ratios, rate ratios, and risk ratios) [21], and considered that ORs and HRs were similar to RRs. When risk estimates for different durations of aspirin use or different levels of aspirin utilization were available, the study-specific RRs were subsequently recalculated in the primary analysis by pooling the risk estimates compared with the reference group. A random effects model was selected to estimate the pooled RRs (95% CI) for the associations between aspirin use and the risk of cancer if the risk estimates for different subtypes of cancer were available. Summary estimates were derived from meta-analyses using random effects models. Studies involving different populations or different types of cancers were treated as independent studies.

To assess the heterogeneity in results of individual studies, *I*^2^ statistic (values of 25%, 50%, and 75% represented cutoff points for low, moderate, and high degrees of heterogeneity, respectively) were used [22]. Publication bias was assessed with Funnel plots, the Begg’s rank correlations and Egger’s regression model. Subgroup analyses for study design, study location, gender, exposure assessment, quality assessment, duration of aspirin use (years), and frequency of aspirin use (tablets/week) were conducted to explore the potential heterogeneity among studies. Subgroup analysis was not conducted for strata with less than five studies. Because time-related biases are common in observational studies of medications and are often responsible for apparent protective effects of drugs, we conducted analyses both including and excluding studies with immortal time bias (bias because of the inclusion of follow-up time during which events cannot occur) [23]. Statistical analyses were performed with Stata version 12.0. (College Station, TX, USA). All reported probabilities (*P* values) were two-tailed with a significance level of 0.05.

## Results

### Literature search and study characteristic

**Fig. 1:**
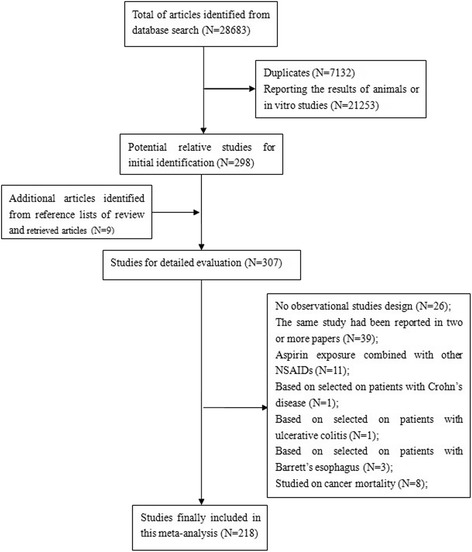
Flow chart of study selection

Figure 1 shows the process for the identification of eligible studies. A total of 28,683 studies were identified and 298 studies remained in the analysis after assessing the titles and abstracts according to the criteria mentioned above. In total, 307 potentially relevant articles were reviewed in their entirety. Among them, 89 articles were further excluded due to the following reasons: 26 articles were not observational design, 11 articles defined exposure combined with other NSAIDs, 8 articles evaluated cancer mortality, 39 articles were duplicate publications on the same subject population, and 5 articles (1 for Crohn’s disease [24], 1 for ulcerative colitis [25], 3 for Barrett’s esophagus [26–28]) included patients with specific diseases. Ultimately, 218 studies with 309 independent reports were included in the present meta-analysis.

The main characteristics of the 218 eligible articles published between 1985 and 2016 are summarized in Tables 1, 2, 3, 4, 5, 6, 7, 8, 9, 10, 11, 12, 13, 14, 15, 16, 17, 18, 19, 20 and 21. Results were presented according to study design. This study altogether included 161 cohort studies and 148 case-control studies. Among them, 135 studies were conducted in North America, 12 in Asia, 61 in Europe, 8 in Oceania, and 2 were multi-country studies. Overall, the summarized RR was 0.89(95%CI: 0.87–0.91), indicating a decreased risk of cancer associated with the use of aspirin. The combined RRs were 0.82 (95% CI: 0.79–0.85) for the case-control studies and 0.94 (95% CI: 0.92–0.97) for the cohort studies. We also observed a apparent beneficial effect of aspirin use when excluding 41 studies deemed to be prone to immortal time bias (RR = 0.87, 95%CI:0.85–0.89) in the meta-analysis.

**Table 1:** Characteristics of included studies- gastric cancer

Study source	Sex	Study period	Source of subjects	No of case	No of control/cohort size	Cancer site	Exposure assessment	Exposure Definition	Adjustment for covariates	Study quality
Case-control studies										
Iqbal U [47], 2017, China	M/F	2001–2011	The Taiwan NHI database	22,574	90,296	Gastric cancer	Prescription	Use at least for 2 months during the 3-year period before the initial cancer diagnosis	1,2,13,14,15,16,17	7
Wang Y [48], 2015, China	M/F	2005–2010	Population from China	175	350	Gastric cancer	Structured questionnaire	Use at least once a week for one year (regular)	2,3,5,6,7,10,18,19,20	7
Gong EJ [49], 2014, Korea	M/F	2000–2010	Asian Medical Center	327	327	Gastric cancer	Self-administered questionnaire	Use of aspirin - not further defined	1,2,3,4,6,8,10,11,12,18,21,22,23,	6
Bertuccio P [50], 2010, Italy	M/F	1997–2007	Population from Italy	229	543	Gastric cancer	Structured questionnaire	Use at least once a week for more than 6 months (regular)	1,2,4,5,6,10,24	7
Figueroa JD [51], 2009, US	M/F	1993–1995	Population from Connecticut, New Jersey, and western Washington state	367	695	Gastric adenocarcinomas	Structured interviews	Use at least once per week for 6 months or more	1,2,3,10,25,26	7
Duan L [52], 2008, US	M/F	1992–1997	Los Angeles County Cencer Surveillance Program	718	1356	Gastric adenocarcinoas	Structured questionnaire	Use of aspirin - not further defined	1,2,3,5,10,20,25,27,28	7
Fortuny J [53], 2007, US	M/F	1980–2002 1993–2004	GHC and HFHS	496	3996	Gastric cancer	Outpatient pharmacy records	No prescription for aspirin (never users)	1,2,25,29,30	7
Akre K [54], 2001, Sweden	M/F	1989–1995	Population from Swedish counties	567	1165	Gastric cancer	Interviews	Ever use of aspirin (ever users)	1,2,9	7
Coogan PF [55], 2000, US	M/F	1977–1998	Population from Baltimore, Boston, New York, and Philadelphia	254	5952	Stomach cancer	Administered questionnaires	Use at least 4 days/week for at least 3 months (regular)	1,2,3,4,5,6,25,32,33,34	8
Zaridze D [56], 1999, Russia	M/F	1993–1997	Moscow City Oncology Hospital and Cancer Research Center and were Moscow City residents	448	610	Stomach cancer	Self-administered questionnaire	Use at least 2 days a week for 6 months or more (regular)	1,5	6
Cohort studies										
Kim YI [57], 2016, Korea^a^	M/F	2004–2010	KNHI database	117	11,598	Gastric cancer	Prescription database	Never make claims for aspirin prescription or less than 6 months of aspirin prescriptions (non-users)	1,2,20, 35	7
Lee J [58], 2012, Korea	M/F	1999–2008	Samsung Medical Center	184	347	Gastric cancer	Prescription	Have aspirin fill prescriptions for at least 6 months	1,2,14	6
Abnet CC [59], 2009, US	M/F	1995–2003	AARP	360	311,115	Gastric cancer	Questionnaire	Any use in the past 12 months	1,2,3,5,6,10,34,36,37	7
Epplein M [60], 2009, US	M/F	1993–2004	Multiethnic Cohort (Hawaii and Los Angeles, California)	643	169,292	Gastric cancer	Self-administered questionnaire	Use any aspirin at least 2 times a week (for 1 month or longer)	1,2,3,6,10,25	7
Lindblad M [61], 2005, UK^a^	M/F	1994–2001	General Practitioners Research Database	1023	1000	Gastric Cancer	Prescription database	Any recorded use of aspirin (ever use)	1,2,3,6,10,28, 31	8
Friis S [62], 2003, Denmark^a^	M/F	1989–1997	Population from North Jutland County	68	29,470	Stomach cancer	Prescription database	75–150 mg once daily(low-dose aspirin)	1,2	8
Schreinemachers DM [63], 1994, US	M/F	1971–1987	The National Health and Examination Survey Ι	39	12,668	Stomach cancer	Self-reported	Use aspirin during the 30-day period before the interview	1,2	6

1 = age, 2 = sex, 3 = smoking, 4 = family history, 5 = educational level, 6 = alcohol intake, 7 = marriage, 8 = fat distribution, 9 = social status, 10 = BMI, 11 = total cholesterol, 12 = triglyceride, 13 = charlson comorbidity index, 14 = statin, 15 = metformin, 16 = ACE inhibitors, 17 = angiotensin II receptor blockers, 18 = helicobacter pylori, 19 = history of diabetes, 20 = resident district, 21 = percent body fat, 22 = HDL cholesterol, 23 = LDL cholesterol, 24 = period of interview, 25 = race, 26 = gastro-esopageal refiux disease, 27 = antacid use, 28 = upper gastrointestinal tract history, 29 = health plan, 30 = duration of continuous, 31 = calendar year enrollment in the health plan at the date of diagnosis, 32 = interview year, 33 = center, 34 = religion, 35 = comorbidity, 36 = total calorie, fibre and calcium intake, 37 = fruit, vegetable and/or vitamin intake, 38 = physical activity, 39 = processed meat intake

*AARP* AARP diet and health study, *GHC* Group Health Cooperative, *HFHS* Henry Ford health system’s health alliance plan, *KNHI* Korean National Health Insurance database

^a^Study deemed to be prone to immortal time bias

**Table 2:** Characteristics of included studies- esophagus cancer

Study source	Sex	Study period	Source of subjects	No of case	No of control/cohort size	Cancer site	Exposure assessment	Exposure Definition	Adjustment for covariates	Study quality
Case-control studies										
Figueroa JD [51], 2009, US	M/F	1993–1995	Population from Connecticut, New Jersey, and western Washington state	282	695	Oesophageal cancer	Structured interviews	Use at least once per week for 6 months or more	1,2,3,10,11,12	7
Sadeghi S [64], 2008, Australia	M/F	2001–2005	Population from Australia	1102	1580	Oesophageal cancer	Questionnaire	Use at least once a week for duration of 6 months or more(regular)	1,2,4,6,10,16,28,29	6
Duan L [52], 2008, US	M/F	1992–1997	Los Angeles County Cencer Surveillance Program	220	1356	Esophageal adenocarcinoma	Structured questionnaire	Use of aspirin - not further defined	1,2,3,5,10,11,14,15,16,	7
Fortuny J [53], 2007, US	M/F	1980–2002 1993–2004	GHC and HFHS	277	3996	Oesophageal cancer	Outpatient pharmacy records	No prescription for aspirin (never users)	1,2,11,17,18	7
Ranka S [65], 2006, UK	M/F	1999–2004	Population from Norfolk	411	1644	Oesophageal cancer	Self-reported,medical admission notes and nursing records	Use of aspirin - not further defined	3,6	8
Anderson LA [66], 2006, Ireland	M/F	2002–2004	The FINBAR study	224	260	Esophageal adenocarcinoma	Interview	Use aspirin at least once weekly for ≥ 6 months	1,2,3,5, 6,10,30,31,	6
Jayaprakash V [67], 2006, US	M/F	1982–1998	RPCI	163	482	Oesophageal cancer	Questionnaire	Use at least once a week for 6 months (regular)	1,2,3,6,10,32,	6
Sharp L [68], 2001, UK	F	1993–1996	Population in England and Scotland	159	159	Oesophagus squamous cell carcinoma	Interview	Daily use of aspirin for at least a month	1,33	7
Cohort sutdies										
Macfarlane TV [69], 2014, UK^a^	M/F	1996–2010	PCCIU database	1197	3585	Oesophageal cancer	Prescription database	Had at least one Prescription (users)	1,2,13,23,24,25,26,27	7
Abnet CC [59], 2009, US	M/F	1995–2003	AARP	228	311,115	Oesophageal adenocarcinoma	Questionnaire	Any use in the past 12 months	1,2,3,5,6,10,20,21,22	7
Lindblad M [61], 2005, UK^a^	M/F	1994–2001	GPRD database	909	1000	Esophageal cancer	Prescription database	Any recorded use of aspirin (ever use)	1,2,3,6, 10,14, 19	8
Friis S [62], 2003, Denmark^a^	M/F	1989–1997	Population of North Jutland County	26	29,470	Oesophagus cancer	Prescription database	75–150 mg once daily (low-dose aspirin)	1,2	8

1 = age, 2 = sex, 3 = smoking, 4 = family history, 5 = educational level, 6 = alcohol intake, 7 = marriage, 8 = Fat distribution, 9 = social status, 10 = BMI, 11 = race 12 = gastroesopageal refiux disease, 13 = other NSAID 14 = upper gastrointestinal tract history, 15 = antacid use, 16 = birthplace,17 = health plan, 18 = duration of continuous enrollment in the health plan at the date of diagnosis, 19 = calendar year, 20 = total calorie, fibre and calcium intake, 21 = fruit, vegetable and/or vitamin intake, 22 = physical activity, 23 = CHD, 24 = stroke, 25 = COX-2 inhibitors, 26 = duration of observation in the database, 27 = deprivation, 28 = household income, 29 = cumulative and frequency of gastroesophageal reflux symptoms 10 y before diagnosis, 30 = location, 31 = job type, 32 = year of completing the questionnaire, 33 = general practice

*AARP* AARP diet and health study, *FINBAR* the factors influencing the Barrett’s adenocarcinoma relationship study, *GHC* Group Health Cooperative, *GPRD* General Practitioners research database, *HFHS* Henry Ford health system’s health alliance plan, *PCCIU* primary care clinical informatics unit database, *RPCI* the Roswell park cancer Institute

^a^Study deemed to be prone to immortal time bias

**Table 3:** Characteristics of included studies- colorectal cancer

Study source	Sex	Study period	Source of subjects	No of case	No of control/cohort size	Cancer site	Exposure assessment	Exposure Definition	Adjustment for covariates	Study quality
Case-control studies										
Iqbal U [47], 2017, China	M/F	2001–2011	The Taiwan NHI database	86,597	346,388	Colorectal cancer	Prescription	Patients had aspirin prescribed at least for 2 months during the 3-year period before the initial cancer diagnosis	1,2,13,14,15,16,17	7
Friis S [70], 2015, Dermark	M/F	1994–2011	Danish Cancer Registry, Aarhus University Prescription Database, Danish National Patient Registry, Danish Civil Registration System	10,280	10,280	Colorectal cancer	Prescription database	Have 2 or more prescriptions for aspirin(ever use)	1,2,14,26,27,28,29,30,31,32,33.	8
Rennert G [71], 2010, Israel	M/F	1988–2006	The MECC	2648	2566	Colorectal cancer	Interviewed	Daily aspirin use for at least 3 years	1,2,7,26	5
Din FV [72], 2010, UK	M/F	2001–2008	SCCS	2279	2907	Colorectal cancer	Questionnaire	Use > 4 tablets/week for > 1 month	1,2,3,6,8,18,19,34,35	4
Harris RE [73], 2008, US	M/F	2003–2004	The CHRI	326	652	Colon cancer	Questionnaire	Use at least once per week for more than 1 year	1,3,4,6,7,8,26,36	5
Kim S [74], 2008, US	M/F	2001–2006	North Carolina Colon Cancer Study II	1057	1019	Colorectal cancer	Questionnaire	Any use of aspirin in the past 5 years (ever users)	1,2,7, 8,18,37, 38,39,40	6
Hoffmeister M [75], 2007, Germany	M/F	2003–2004	The Rhine–Neckar–Odenwald region in the South-West of Germany	477	517	Colorectal cancer	Questionnaire	Use at least 2 times per week for at least 1 year(current regular use)	1,2,3,4,5,6,8,22,27,30,41,42,43	8
Slattery ML [76], 2006, US	M/F	1991–1994	KPMCP	2351	2972	Colorectal cancer	Questionnaire	Use at least three times a week for 1 month(regular)	1,2,7	7
Macarthur M [77], 2005, UK	M/F	1998–2000	Grampian Health Board residents	264	408	Colorectal cancer	Questionnaire	Use aspirin every day for a month or more(regular)	1,2	6
Juarranz M [78], 2002, Spain	M/F	1995–1996	The Research Unit of the Council of Health and Social Services of the Community ofMadrid	196	228	Colon cancer	Questionnaire	Consider aspirin use as a continuous numeric variable in milligrams/week -not further defined	1,2	8
Evans RC [79], 2002, UK	M/F	–	Merseyside and Cheshire Cancer Registry	512	512	Colorectal cancer	Questionnaire	Use at least once per day(regular)	1,2,26,38	8
Neugut AI [80], 1998, US	M/F	1989–1992	Columbia-Prebyterian Medical Center	256	322	Colon cancer	Medical record	Use aspirin-not further defined	1,4,5	6
Rosenberg L [81], 1998, US	M/F	1992–1994	Hospital in Massachusetts	942	935	Large bowel carcinoma	Questionnaire	Use at least 4 days a week for at least 3 months	1,2	9
La VC [82], 1997, Italy	M/F	1992–1996	Population from Italian areas	1357	1891	Colorectal adenoma	Questionnaire	Use more than four times per week for > 6 months	1,2,5,6,8,18,26,34, 43	7
Reeves MJ [83], 1996, US	F	1991–1992	Wisconsin Cancer Reporting system	21	22	Colorectal cancer	Self-reported	Use at least one table twice weekly or more than at least 12 months	1,4,8,30	8
Suh O [84], 1993, US	M/F	1982–1991	Roswell Park Tumor Registry and Diagnostic Index	830	1662	Colorectal adenoma	Questionnaire	Use aspirins for at least 1 year(users)	1,2,5,26	9
Kune GA [85], 1988, Australia	M/F	1980–1981	Population in Melbourne	715	727	Colorectal adenoma	Questionnaire, hospital records, and interview	Use aspirin “daily” “weekly” or “don’t know- not further defined”	1,2	8
Cohort studies										
Park SY [86], 2017, US	M/F	1993–2012	The MEC Study	3879	183,199	Colorectal cancer	Questionnaire	Had ever use of aspirin	1,3,4,6,8,18,19,27,30,34,37,43, 48,49	8
Kim C [87], 2016, US	M	1982–2000	Physicians Health Study	268	446	Colorectal cancer	Questionnaire	Use of aspirin- not further defined	6,8,18,19, 20	9
Soriano LC [88], 2016, UK(STUDY 1)	M/F	2000–2011	THIN	3033	10,000	Colorectal cancer	Prescription	No recorded use at any time(non user)	1,2,3,8,21, 22,24,25	9
Soriano LC [88], 2016, UK(STUDY 2)	M/F	2001–2012	THIN	3174	10,000	Colorectal cancer	Prescription	No recorded use at any time(non user)	1,2,3,8,21,22,23	9
Soriano LC [88], 2016, UK(STUDY 3)	M/F	2001–2012	THIN	12,333	20,000	Colorectal cancer	Prescription	No recorded use at any time(non user)	1,2,3,8,21,22	9
Vaughan LE [89], 2016, US	F	2004–2011	IWHS	218	14,386	Colon cancer	Questionnaire	Never use aspirin (non-user)	1,3,8,22	8
Cao Y [8], 2016, US	M/F	1980–2010 1986–2012	NHS and HPFS	2895	135,965	Colorectal cancer	Questionnaire	Use at least 2 times per week(regular)	3,4,6,7,8,18,19,27,30,34,37,42,43,49,50,51,52,53	9
Lin CC [90], 2015, China^a^	M/F	2000–2009	The Longitudinal Health Insurance Database	467	60,828	Colorectal cancer	Prescription database	Use any low-dose aspirin (75–165 mg)	1,2,54,55	8
Hollestein LM [91], 2014, Netherlands^a^	M/F	1998–2010	PHARMO and the Eindhoven Cancer Registry	972	109,276	Colorectal cancer	Prescription database	Low dose aspirin (≤100 mg daily)- not further defined	1,2,56,72	8
Brasky TM [92], 2014, US	F	1998–2010	WHI	1397	140,933	Colorectal cancer	Self-administered questionnaires	Use at both baseline and year 3 visits (consistent)	1,3,4,5,6,7,8,37,18,19,22,26,27,33,43,50,57,58,59,60,61,62,63,64,65,66,67,68,69,70,71	9
Brasky TM [93], 2012, US	M/F	2002–2008.12.30	The VITAL	451	64,847	Colorectal cancer	Questionnaire	Use ≥1 day/week for ≥ 1 year(regular)	1,3,4,5,6,7,8,9,10,18,19,22,28,30,33,42,43, 50,67,68,69,71	8
Ruder EH [94], 2011, US	M/F	1996–2006	National Institutes of Health-AARP Diet and Health Study	3894	301,240	Colorectal cancer	Self-administered questionnaire	Use aspirin during the previous 12 months	1,2,3,4,5, 6,7,8,18,27	7
Friis S [95], 2009, Denmark	M/F	1995–2006	Danish Diet, Cancer, and Health Study	615	51,053	Colorectal cancer	Questionnaire	Use fewer than 2 pills per month (nonuse)	1,2,6,8,14,22,27,30,	7
Siemes C [96], 2008, Netherlands	M/F	1992–2004	The Rotterdam Study	195	7621	Colorectal cancer	Questionnaire and prescriptions	The absence of a prescription for any non-aspirin or aspirin NSAID(no use)	1,2,3,8,18, 27,34,70,73,74	8
Vinogradova Y [97], 2007, UK^a^	M/F	1995–2005	QRESEARCH database	1226	5369	Colorectal cancer	Prescription database	Receive ≥1 prescription for aspirin in the 13 to 48 months before index date	3, 8,22,41	8
Jacobs EJ [98], 2007, US	M/F	1992–2003	Cancer Prevention Study II Nutrition Cohort	1861	146,113	Colorectal cancer	Questionnaire	Use at least 30 “times” per month(daily use of adult-strength)	1,2,3,5,7,18,22,27,28,30,36,68,52,53	8
Larsson SC [99], 2006, Sweden	M/F	1998–2005	Swedish Mammography Cohort and Cohort of Swedish Men	705	74,250	Colorectal cancer	Questionnaire	Aspirin use- not further defined	1,2,3,4,5,8,18 28	9
Muscat JE [100], 2005, US	M/F	1983–1999	The Framingham Heart study	145	433	Colorectal cancer	Questionnaire	Never/< 1/week, 1–3/week, > 3/week	1,2,3,44	9
Rahme E [101], 2003, Canada	M/F	1997–2001	RAMQ	179	2568	Colorectal adenoma	Prescription	Use at least 1 year	45,46,47	7
Rodríguez LAG [102], 2001, UK^a^	M/F	1994–1997	The GPRD	2002	943,903	Colorectal cancer	Prescription database	Never received a single prescription(non-user)	1,2	8
Schreinemachers DM [63], 1994, US	M/F	1971–1987	The National Health and Examination Survey Ι	169	12,668	Colorectal cancer	Self reported	Use aspirin during the 30-day period before the interview	1,2	6
Paganini-Hill A [103], 1989, US	M/F	1981–1988	Population from Leisure World, Laguna Hills, US	181	13,870	Colon cancer	Questionnaire	Aspirin use: none,<daily, daily	2	4

1 = age, 2 = sex, 3 = smoking, 4 = family history, 5 = educational level, 6 = alcohol intake, 7 = race, 8 = BMI, 9 = marital status, 10 = self-rated health, 11==C-reactive protein level, 12 = cholesterol, 13 = Charlson comorbidity index, 14 = statin, 15 = metformin, 16 = ACE inhibitors, 17 = angiotensin II receptor blockers, 18 = physical activity 19 = fruit, vegetable and/or vitamin intake, 20 = seafood and dairy foods intake, 21 = number of PCP visits in the year before the index date, 22 = other NSAIDs, 23 = paracetamol, 24 = insulin, 25 = oral steroids, 26 = area (county/region), 27 = hormone replacement therapy, 28 = history of diabetes mellitus, 29 = history of cholecystectomy, 30 = history of colonoscopy, 31 = chronic obstructive pulmonary disease or asthma,32 = antidepressants, 33 = migraine,34 = total energy intake, 35 = deprivation index, 36 = hypertension ampling probability, 37 = ever use of calcium supplements in the past 5 years, 38 = primary care practitioner, 39 = dietary fat intake, 40 = sampling probability, 41 = morbidity (diabetes, ischemic heart disease, hypertension, stroke, colitis, rheumatoid arthritis, and osteoarthritis), 42 = former health checkup, 43 = red meat, 44 = Nitro-vasodilator use, 45 = number of drugs, 46 = number of physician encounters, 47 = all-cause hospitalization in prior year, 48 = dietary fiber, 49 = folate, 50 = height, 51 = Alternate Healthy Eating Index-2010, 52 = PSA test in past 2 y, 53 = mammogram in past 2 y, 54 = duration of diabetes, 55 = propensity score at baseline, 56 = unique number of hospitalizations in the year prior to start of follow up, 57 = observational study enrollment, 58 = diet modification trial enrollment, 59 = screening for cancer, 60 = age at menarche, 61 = age at menopause, 62 = gravidity, 63 = age atfirst birth, 64 = duration of estrogen therapy, 65 = duration of combined postmenopausal hormone therapy, 66 = hysterectomy status, 67 = use of antihypertensive medication, 68 = history of coronary heart disease, 69 = use of cholesterol-lowering medication, 70 = history of arthritis, 71 = history of Ulcer, 72 = unique number of dispensing

*AARP* AARP diet and health study, *CHRI* Cancer Hospital and Richard J. Solove Research Institute, *GPRD* General Practitioners Research Database, *HPFS* Health Professionals follow-up study, *IWHS* Iowa Women’s Health Study, *KPMCP* Kaiser Permanente Medical Care Program of Northern California, *MEC* Multiethnic Cohort Study, *MECC* the molecular epidemiology of colorectal cancer, *NHS* nurses’ health study, *RAMQ* Re′gie de l’Assurance Maladie du Que’bec, *SCCS* study of colorectal cancer in Scotland, *THIN* the health improvement Network, *VITAL* the vitamins and lifestyle, *WHI* women’s health initiative

^a^Study deemed to be prone to immortal time bias

**Table 4:** Characteristics of included studies- hepato-biliary cancer

Study source	Sex	Study period	Source of subjects	No of case	No of control/cohort size	Cancer site	Exposure assessment	Exposure Definition	Adjustment for covariates	Study quality
Case-control studies										
Choi J [104], 2016, US	M/F	2000–2014	Patients seen at the Mayo Clinic	2395	4769	Cholangiocarcinoma	Electronic medical record	Use at least once per week at the index date	1,2,3,11,12,13,14,15	9
Yang B [105], 2016, UK	M/F	1988–2011	CPRD	814	3180	Primary liver cancer	Medical records database	Had two or more aspirin prescriptions recorded prior to the index date(ever use)	3,6,10,15,16,17,18	7
Burr NE [106], 2014, UK	M/F	2004–2010	NNUH and LGH	81	275	Cholangiocarcinoma	Letters from general practitioners (GPs), hospital clerkings, surgical records, nursing notes and radiological reports	Drug was recorded in any of the data sources	1,2,3,15	7
Cohort studies										
Kim G [107], 2017, Korea	M/F	2003–2012	NHIS-NSC	229	1145	Hepatocellular carcinoma	Prescription	At least one prescription of aspirin between the cohort entry and the index date	1,2,19,20,	6
Petrick JL [108], 2015, US	M/F	from 1993	AARP,AHS, USRT,BCDDP, PLCO,HPFS, CPSII, BWHS WHI,NHS	904	1,084,133	Hepatocellular carcinoma and intrahepatic cholangiocarcinoma	Questionnaire	Any reported aspirin use in the 12 months prior to baseline	1,2,3,6,10,11,15, 21	7
Liu E [109], 2005, China	M/F	1997–2001	Population from Shanghai	368	1013	Gallbladder Cancer	Questionnaire	Use at least twice a week for longer than a month 1 year before interview	1,2,5, 22	6
S Friis [62], 2003, Denmark^a^	M/F	1989–1997	Population from North Jutland County	21	29,470	Liver cancer	Prescription database	75–150 mg once daily(low-dose aspirin)	1,2	8

1 = age, 2 = sex, 3 = smoking, 4 = family history, 5 = educational level, 6 = alcohol intake, 7 = marriage, 8 = Fat distribution, 9 = social status, 10 = BMI, 11 = race,12 = primary sclerosing cholangitis (PSC), 13 = non-PSC-related cirrhosis, 14 = biliary tract diseases, 15 = diabetes, 16 = hepatitis B or C virus infection, 17 = rare metabolic disorders, 18 = use of paracetamol, antidiabetic medications, and statins,19 = follow–up duration, 20 = the date of the diabetes diagnosis, 21 = cohort (AARP, AHS, USRT, PLCO, HPFS, CPSII, IWHS, BWHS, WHI, NHS), 22 = biliary stone status

*AARP* AARP diet and health study, *AHS* Agriculture Health Study, *BCDDP* the breast cancer detection demonstration project, *BWHS* black women’s health study, *CPRD* clinical practice research datalink, *CPSII* cancer prevention study II, *HPFS* Health Professionals follow-up study, *IWHS* Iowa Women’s Health Study, *LGH* Leicester General Hospital NHS Trust, *NHIS-NSC* National Health Insurance Service National Sample Cohort, *NHS* nurses’ health study, *NNUH* Norfolk and Norwich University Hospital, *PLCO* prostate, lung, colorectal and ovarian cancer screening trial, *USRT* United State Radiologic Technologist Study, *WHI* women’s health initiative

^a^Study deemed to be prone to immortal time bias

**Table 5:** Characteristics of included studies- pancreatic cancer

Study source	Sex	Study period	Source of subjects	No of case	No of control/cohort size	Cancer site	Exposure assessment	Exposure Definition	Adjustment for covariates	Study quality
Case-control studies										
Risch HA [110], 2017, China	M/F	2006–2011	Our Shanghai study	761	794	Pancreatic cancer	In-person questionnaire interviews	Use at least one tablet per week for 3 months or longer(regular)	1,2,3,5,7,10,50,51	7
Kho PF [111], 2016, Australia	M/F	2007–2011	The QPCS	522	652	Pancreatic cancer	Questionnaire	Long-term use of aspirin ((> 2 years)	1,2,3,6,10	8
Streicher SA [112], 2014, US	M/F	2005–2009	Population from Connecticut	360	682	Pancreatic cancer	Questionnaire	Use at least once a week on average, for 3 months or more	1,2,3,5,7,10,11,52	8
Tan XL [113], 2011, US	M/F	2004–2010	Patients from the Mayo Clinic	740	1043	Pancreatic cancer	Questionnaire	Use aspirin ≥1 day per month	1,2,3,7,10	6
Pugh TFG [114], 2011, UK	M/F	2004–2007	Clinical management databases in Norfolk and Leicestershire	206	251	Pancreatic cancer	Medical records	Use of aspirin - not further defined	1,2,3,7	6
Bonifazi M [115], 2010, Italy	M/F	1991–2008	Patients in in the province of Pordenone and in the greater Milan area, northern Italy	308	477	Pancreatic cancer	Questionnaire	Use at least once a week for more than 6 months(regular)	1,2,3,5,7,10,53,54	8
Menezes RJ [116], 2002, US	M/F	1982–1998	The RPCI	194	585	Pancreatic cancer	Patient Epidemiology Data System (PEDS) and questionnaire	Use at least once a week for six consecutive months(regular)	1,3,4	5
Cohort studies										
Cao Y [8], 2016, US	M/F	1980–2010 1986–2012	NHS and HPFS	607	135,965	Pancreatic cancer	Questionnaire	Use at least 2 times per week(regular)	3,4,6,10,11,12,13,14,15,16,17,18,19,20,21,22,23,24	9
Brasky TM [92], 2014, US	F	1998–2010	WHI	397	142,330	Pancreatic cancer	Self-administered questionnaires	Use at both baseline and year 3 visits (consistent)	1,3,4,5, 6,10,11,17,18,19,25,26,28,29,30,31,32,34,35,36,37,38,39,40,41,42,43,44,45,46,47,48	9
Bradley MC [117], 2010, UK^a^	M/F	1995–2006	GPRD	564	3984	Pancreatic cancer	Prescription Database	Use 300 mg or more a day (high-dose)	3,6,7,10, 25,27,47,55,	8
Jacobs EJ [98], 2007, US	M/F	1992–2003	Cancer Prevention Study II Nutrition Cohort	404	146,113	Pancreatic cancer	Questionnaire	Use at least 30 “times” per month(daily use of adult-strength)	1,2,3,5,7,10,11, 15,16,17,18,20,25, 45, 49	8
Friis S [62], 2003, Denmark^a^	M/F	1989–1997	Population from North Jutland County	62	29,470	Pancreatic cancer	Prescription Database	75–150 mg once daily(low-dose aspirin)	1,2	8
Anderson KE [118], 2002, US	F	1992–1999	IWHS	80	28,283	Pancreatic cancer	Questionnaire	Never use any type of medication (never use)	1,3,7,19	7
Schreinemachers DM [63], 1994, US	M/F	1971–1987	The National Health and Examination Survey Ι	30	12,668	Pancreatic cancer	Self reported	Use aspirin during the 30-day period before the interview	1,2	6

1 = age, 2 = sex, 3 = smoking, 4 = family history, 5 = educational level, 6 = alcohol intake, 7 = diabetes, 8 = Fat distribution, 9 = social status, 10 = BMI, 11 = race, 12 = folate, 13 = height, 14 = Alternate Healthy Eating Index-2010, 15 = PSA test in past 2 y, 16 = mammogram in past 2 y, 17 = hormone replacement therapy, 18 = physical activity, 19 = fruit, vegetable and/or vitamin intake, 20 = history of colonoscopy, 21 = total energy intake, 22 = ever use of calcium supplements in the past 5 years, 23 = former health checkup, 24 = red meat, 25 = other NSAIDs, 26 = area (county/region), 27 = prior cancer, 28 = migraine, 29 = ever use of calcium supplements in the past 5 years, 30 = red meat, 31 = Nitro-vasodilator use, 32 = height, 33 = unique number of hospitalizations in the year prior to start of follow up, 34 = observational study enrollment, 35 = diet modification trial enrollment, 36 = screening for cancer, 37 = age at menarche, 38 = age at menopause, 39 = gravidity, 40 = age atfirst birth, 41 = duration of estrogen therapy, 42 = duration of combined postmenopausal hormone therapy, 43 = hysterectomy status, 44 = use of antihypertensive medication, 45 = history of coronary heart disease, 46 = use of cholesterol-lowering medication, 47 = history of arthritis, 48 = history of ulcer, 49 = hypertension, 50 = H. pylori CagA seropositivity, 51 = ABO blood group A vs. non-A, 52 = ABO blood group O vs. non-O, 53 = center, 54 = year of interview, 55 = history of chronic pancreatitis

*GPRD* General Practitioners Research Database, *HPFS* Health Professionals follow-up study, *IWHS* Iowa Women’s Health Study, *NHS* nurses’ health study, *QPCS* the Queensland Pancreatic Cancer Study, *RPCI* the Roswell Park Cancer Institute, *WHI* women’s health initiative

^a^Study deemed to be prone to immortal time bias

**Table 6:** Characteristics of included studies- lung cancer

Study source	Sex	Study period	Source of subjects	No of case	No of control/cohort size	Cancer site	Exposure assessment	Exposure Definition	Adjustment for covariates	Study quality
Case-control studies										
Iqbal U [47], 2017, China	M/F	2001–2011	The Taiwan NHI database	68,409	273,636	Lung cancer	Prescription	Patients had aspirin prescribed at least for 2 months during the 3-year period before the initial cancer diagnosis	1,2,13,14,15,16,17	7
Lim WY [119], 2012, Singapore	F	2005–2008	Population from Chinese	252	556	Lung cancer	Questionnaire	Use twice a week or more, for a month or more(regular)	1, 3,4,5,19,34	7
McCormack VA [120], 2011, US	M/F	–	AHFTS	977	683	Lung cancer	Interview	–	1,3,5,11	7
McCormack VA [120], 2011, US	M/F	–	Population from Boston	768	123	Lung cancer	–	–	1,3,5,11	7
McCormack VA [120], 2011, US	M/F	–	Population from Florida	467	889	Lung cancer	–	–	1,3,5,11	7
McCormack VA [120], 2011, US	M/F	–	Population from Hawaii	629	588	Lung cancer	–	–	1,3,5,11	7
McCormack VA [120], 2011, US	M/F	–	MSKCC	102	101	Lung cancer	–	–	1,3,5,11	7
McCormack VA [120], 2011, US	M/F	–	NELCS	276	251	Lung cancer	–	–	1,3,5,11	7
McCormack VA [120], 201, Israel	M/F	–	NICCC	280	270	Lung cancer	–	–	1,3,5,11	7
Kelly JP [121], 2008, US	M/F	1976–2007	Patients in Boston Baltimore New York and Philadelphia	1884	6251	Lung cancer	In-person interview	Use at least 4 days per week for at least three continuous months(regular)	1,2,3,4,6,29, 30,36	6
Van Dyke AL [122], 2008, US	F	2001–2005	Metropolitan Detroit Cancer Surveillance System, a participant in the National Cancer Institute’s Surveillance	580	541	Lung Cancer	Questionnaire	Had taken any aspirin	1,3,4,5,10,11, 3135,37	7
Harris RE [123], 2007, US	M/F	2002–2004	The Ohio State University Medical Center, Columbus, Ohio	375	654	Lung Cancer	Interview	Use no more than one pill per week for less than 1 year(nonuser)	1,2,3,5,6,10,11,35	7
Muscat JE [124], 2003, US	M/F	1992–2000	Hospitals in New York and Washington, D.C	997	918	Lung Cancer	Questionnaire	Use three tablets per week for 1 or more years(regular)	1,2,3,4	7
Moysich KB [125], 2002, US	M/F	1982–1998	RPCI	868	935	Lung Cancer	Epidemiological questionnaire	Use at least once a week for one year(regular)	1,3,4	8
Cohort studies										
Cao Y [8], 2016, US	M/F	1980–2010 1986–2012	NHS and HPFS	2430	135,965	Lung cancer	Questionnaire	Use at least 2 times per week(regular)	3,5,6,7,8,9,10,11,12,18,19,20,21,22,23,24,25,28	9
Baik CS [126], 2015, US	F	1993–2010	WHI	1902	143,841	Lung cancer	Questionnaire	Use at least twice a week in each of the two weeks preceding the interview(regular)	1,3,5,6,10,11,19,25,31,50,51,52	8
Hollestein LM [91], 2014, Netherlands^a^	M/F	1998–2010	PHARMO and the Eindhoven Cancer Registry	915	109,276	Lung cancer	Prescription database	Low dose aspirin (≤ 100 mg daily)- not further defined	1,2,26,27	8
Brasky TM [127], 2012, US	M/F	2000–2007	The VITAL cohort	100	69,919	Lung cancer	The baseline questionnaire	Use aspirin ≥1 day/week for ≥ 1 year(regular)	1,2,3,4,5,10,11, 29,35,46,53,54	8
McCormack VA [120], 2011, US	M/F	–	DDCHS	812	55,396	Lung cancer	Questionnaire	–	1,3,5,11,	7
Siemes C [96], 2008, Netherland	M/F	1992–2004	The Rotterdam Study	134	7621	Lung cancer	Questionnaire and prescriptions.	The absence of a prescription for any non-aspirin or aspirin NSAID(no use)	1,2,10,18,21,25, 35,55,56,57	8
Olse JH [128], 2008, Dermark^a^	M/F	2002–2005	Danish Diet, Cancer and Health prospective cohort study	282	390	Lung cancer	Questionnaire and prescription database	Any use of aspirin or 1 year or more before the index date	1,2,3,4,38,39	7
Hernández-Díaz S [129], 2007, UK^a^	M/F	1995–2004	THIN database	4336	10,000	Lung cancer	THIN database	Had recorded prescription at any time before the index date	1,2,3,6,10, 14,33,35,40,41,42,43,44,45,46,47,48	8
Jacobs EJ [98], 2007, US	M/F	1992–2003	Cancer Prevention Study II Nutrition Cohort	1815	146,113	Lung cancer	Questionnaire	Use at least 30 “times” per month(daily use of adult-strength)	1,2,3,4,9,10,11, 18,25,28,29, 31,32,33	8
Hayes JH [130], 2006, US	F	1992–2002	IWHS	403	27,162	Lung cancer	Questionnaire	Never, less than one weekly, once weekly, two to five times weekly, and six or more times weekly	1,3,4,6,10,19, 29,58	7
Akhmedkhanov A [131], 2002, US	F	1994–1996	NYU and Women’s Health Study cohort.	81	808	Lung cancer	Questionnaire	Use three or more times per week for a period of 6 months or longer	1,3,4,49	7
Schreinemachers DM [63], 1994, US	M/F	1971–1987	The National Health and Examination Survey Ι	163	12,668	Lung cancer	Self reported	Use aspirin during the 30-day period before the interview	1,2	6
Paganini-Hill A [103], 1989, US	M/F	1981–1988	Population in Leisure World, Laguna Hills, US	111	13,870	Lung cancer	Questionnaire	Aspirin use: none,<daily, daily	2	4

1 = age, 2 = sex, 3 = smoking, 4 = education level, 5 = family history, 6 = alcohol intake, 7 = height, 8 = Alternate Healthy Eating Index-2010, 9 = PSA test in past 2 y, 10 = BMI, 11 = race, 12 = folate, 13 = Charlson comorbidity index, 14 = statin, 15 = metformin, 16 = ACE inhibitors, 17 = Angiotensin II receptor blockers, 18 = physical activity, 19 = fruit, vegetable and/or vitamin intake, 20 = history of colonoscopy, 21 = total energy intake, 22 = ever use of calcium supplements in the past 5 years, 23 = former health checkup, 24 = red meat, 25 = hormone replacement therapy, 26 = unique number of dispensing, 27 = unique number of hospitalizations in the year prior to start of follow up, 28 = mammogram in past 2 y, 29 = other NSAIDs, 30 = area (county/region), 31 = history of coronary heart disease, 32 = diabetes, 33 = hypertension, 34 = housing type, 35 = history of arthritis, 36 = interview year, 37 = history of COLD, 38 = study, 39 = use of acetaminophen, 40 = smoking cessation advice by general practitioner, 41 = smoking cessation treatment, 42 = number of visits to general practitioner, 43 = number of referrals, 44 = use of oral corticosteroids, 45 = antihypertensives and other lipid-lowering drugs, 46 = chronic obstructive pulmonary disease, 47 = cerebrovascular disease, 48 = ischemic heart disease, 49 = menopausal status, 50 = age started and years since quitting smoking, 51 = emphysema, 52 = randomization arm of the DM trial, 53 = history of ulcer, migraine or chronic headache, osteoarthritis or chronic joint pain, 54 = coronary artery disease, 55 = C-reactive protein level, 56 = pack years of smoking, 57 = cholesterol, 58 = any heart disease/heart attack

*AHFTS* American Health Foundation Tobacco Study, *DDCHS* Danish Diet Cancer and Health Study, *HPFS* Health Professionals follow-up study, *IWHS* Iowa Women’s Health Study, *MSKCC* Memorial Sloan-Kettering Cancer Center, *NELCS* New England Lung Cancer study, *NHS* nurses’ health study, *NICCC* National Israel Cancer Control Center, *NYU* New York University, *RPCI* the Roswell Park Cancer Institute, *THIN* the Health Improvement Network, *VITAL* the vitamins and lifestyle, *WHI* women’s health initiative

^a^Study deemed to be prone to immortal time bias

**Table 7:** Characteristics of included studies- breast cancer

Study source	Sex	Study period	Source of subjects	No of case	No of control/cohort size	Cancer site	Exposure assessment	Exposure Definition	Adjustment for covariates	Study quality
Case-control studies										
Iqbal U [47], 2017, China	F	2001–2011	The Taiwan NHI database	65,491	435,364	Breast cancer	Prescription	Patients had aspirin prescribed at least for 2 months during the 3-year period before the initial cancer diagnosis	1,2,13,14, 15,16,17	7
Dierssen-Sotos T [132], 2016, Spain	F	2008–2013	The MCC study	1736	1909	Breast cancer	Questionnaire	Use of aspirin- not further defined	1,3,4,5,10,30,36,40,50,51	8
Cui Y [133], 2014, US	F	2001–2011	Nashville Breast Health Study	2154	1831	Breast cancer	Telephone interview	Use aspirin three or more times a week for a minimum duration of 1 year(regular)	1,3,4,5,6,11,18,25,40,50,51,52,53	7
Brasky TM [134], 2010, US	F	1996–2001	WEB Study	1057	2094	Breast cancer	Self-reported	Use 0 days/month (non-users)	1,4,5,11,25,29,36,37,38,53,	6
Cronin-Fenton DP [135], 2010, Denmark	F	1991–2006	Population from North Jutland and Aarhus counties, Denmark	8195	81,950	Breast cancer	Danish healthcare databases	Use at least 2 prescriptions within 2 years of diagnosis(recent use)	25,31,39	8
Slattery ML [136], 2007, US	F	1999–2004	Population from the southwestern United States (4-Corner’s Breast Cancer Study)	2325	2525	Breast cancer	Questionnaire	Use at least thrice weekly for at least 1 month(regular)	1,10,18,38,54,55,56	7
Harris RE [137], 2006, US	F	2003–2004	CHRI	277	493	Breast cancer	Questionnaire	Use at least two times per week for 2 years or more	1,3,5,6,10,38,51	7
Swede H [138], 2005, US	F	1982–1998	The Roswell Park Cancer Institute	1478	3383	Breast cancer	Questionnaire	Use aspirin at least once a week for at least 1 year(regular)	5,10,36,40,53	6
Zhang YQ [139], 2005, US	F	1976–2002	The Case-Control Surveillance Study Revisited	2406	1554	Breast cancer	Questionnaire	Use at least four times per week for 3 or more continuous months(regular)	1,4,5,6,10,11, 36,37,38,40,41,53,54,61,62,63,64	5
Terry MB [140], 2004, US	F	1996–1997	The Long Island Breast Cancer Study Project	1442	1420	Breast cancer	Questionnaire	Use at least once a week for 6 months or longer(ever use)	1,10,29,31	6
Moorman PG [141], 2003, US	F	1996–2000	Phase II of the Carolina Breast Cancer and Carcinoma In Situ Study	500	2631	Breast cancer	Questionnaire	Use at least 8 days a month for three or more months(regular)	1	6
Cotterchio M [142], 2001, Canada	F	1996–1998	Population in Canada	2696	2600	Breast cancer	Questionnaire	Daily use for≥ 2 months(any use)	1,39,53	6
Neugut AI [80], 1998, US	F	1989–1992	Columbia-Prebyterian Medical Center	252	176	Breast cancer	Medical record	Use aspirin-not further defined	1,4,5	6
Cohort studies										
Cao Y [8], 2016, US	F	1980–2010 1986–2012	NHS and HPFS	7424	135,965	Breast cancer	Questionnaire	Use at least 2 times per week(regular)	3,5,6,7,8,9,10,11,12,18,19,20,21,22,23,24,25,28	9
Kim S [143], 2015, US	F	2003–2013	Sister Study	2118	50,884	Breast cancer	Questionnaire	Use at least once a week(current user)	4,5,10,11,40,51,53, 67	8
Hollestein LM [91], 2014, Netherlands^a^	F	1998–2010	PHARMO and the Eindhoven Cancer Registry	585	55,597	Breast cancer	Prescription database	Low dose aspirin (≤ 100 mg daily)- not further defined	1,2,26,27	8
Brasky TM [92], 2014, US	F	1998–2010	WHI	5401	142,330	Breast cancer	Self-administered questionnaires	Use at both baseline and year 3 visits (consistent)	1,3,4,5, 6,7,10,11,18,19,22,24,25,29,30,31,,32,33,34,35,36,37,38,39,40,4142,43,44,45,46,47	9
Bardia A [144], 2011, US	F	1986–2005	The IWHS	1581	26,580	Breast cancer	Questionnaire	Ever use aspirin- not further defined	1,3,4,5,6,10, 18,25,36,37,38,39,40,68,69	8
Bosco JL [145], 2011, US	F	1995–2007	BWHS	1275	59,000	Breast cancer	Questionnaire	Use aspirin ≥ 3 days per week (regular)	1,3,4,10,18,25,29,70	9
Eliassen AH [146], 2009, US	F	1989–2003	NHS II	1229	112,292	Breast cancer	Questionnaire	Use aspirin ≥2 times per week(regular)	5,6,7,10,36,38,40,53, 68,71	9
Friisa S [147], 2008, Denmark	F	1993–2003	The prospective Diet, Cancer and Health cohort study	396	28,695	Breast cancer	Questionnaire	Use more than one pill per month	1,4,25,38,50,53	7
Gierach GL [148], 2008, US	F	1995–2003	AARP	4451	126,124	Breast cancer	Questionnaire	Ever use aspirin- not further defined	1,5,6,11,25,29,40,49, 72	7
Ready A [149], 2008, US	F	2000–2004	VITAL cohort	479	35,323	Breast cancer	Questionnaire	Use at least once a week for a year during the last 10 years(any use)	1,5,6,10,11,19,28,29,36,37,40,72, 73,74	7
Siemes C [96], 2008, Netherland	F	1992–2004	The Rotterdam Study	175	7621	Breast cancer	Questionnaire and prescriptions.	The absence of a prescription for any non-aspirin or aspirin NSAID(no use)	1,3,10,25,36,37,50, 75	8
Jacobs EJ [98], 2007, US	F	1992–2003	Cancer Prevention Study II Nutrition Cohort	3121	76,303	Breast cancer	Questionnaire	Use at least 30 “times” per month(daily use of adult-strength)	1,3,4,10,11,18,20,25, 28,29,45,48,49	8
Gill JK [150], 2007, US	F	1993–2002	Multiethnic Cohort	1457	98,920	Breast cancer	Questionnaire	Use at least two times per week for 1 month or longer	1,4,5,6,10,11, 25,28,36,37,40,50,51,76	7
Gallicchio L [151], 2007, US	F	1989–2006	CLUE II (“Give us a Clue to Cancer and Heart Disease”)	418	15,651	Breast cancer	Questionnaire	Use aspirin in the last 48 h(current user)	1	7
Marshall SF [152], 2005, US	F	1995–2001	The California Teachers Study	2391	114,640	Breast cancer	Questionnaire	Use at least once a Week(regular)	1,3,5,6,10,11,18, 25,28,51,53,59,77	9
Rahme E [153], 2005, Canada^a^	F	1998–2202	RAMQ	664	23,573	Breast cancer	Prescription database	Ever use aspirin during the year prior to the index date	1,25,28,53,57,58,60	7
Rodríguez LA [154], 2004, UK^a^	F	1995–2001	GPRD	3708	23,708	Breast cancer	Prescription database	No recorded use at any time before the index date(nonuser)	1,3,6,10,25,29,53, 62,65,66	8
Harris RE [155], 1999, US	F	1991–1996	Population from The Ohio State University Comprehensive Cancer Center in Columbus, Ohio	316	32,505	Breast cancer	Questionnaire	Use aspirin ≥1 pill per week	1	5
Schreinemachers DM [63], 1994, US	F	1971–1987	The National Health and Examination Survey Ι	147	12,668	Breast cancer	Self reported	Use aspirin during the 30-day period before the interview	1,2	6
Paganini-Hill A [103], 1989, US	F	1981–1988	Population from Leisure World, Laguna Hills, US	214	13,870	Breast cancer	Questionnaire	Aspirin use: none,<daily, daily	2	4

1 = age, 2 = sex, 3 = smoking, 4 = education level, 5 = family history, 6 = alcohol intake, 7 = height, 8 = Alternate Healthy Eating Index-2010, 9 = PSA test in past 2 y, 10 = BMI, 11 = race, 12 = folate, 13 = Charlson comorbidity index, 14 = statin, 15 = metformin, 16 = ACE inhibitors, 17 = Angiotensin II receptor blockers, 18 = physical activity, 19 = fruit, vegetable and/or vitamin intake, 20 = history of colonoscopy, 21 = total energy intake, 22 = ever use of calcium supplements in the past 5 years, 23 = former health checkup, 24 = red meat, 25 = hormone replacement therapy, 26 = unique number of dispensing, 27 = unique number of hospitalizations in the year prior to start of follow up, 28 = mammogram in past 2 y, 29 = other NSAIDs, 30 = area (county/region), 31 = migraine, 32 = Nitro-vasodilator use, 33 = observational study enrollment, 34 = diet modification trial enrollment, 35 = screening for cancer, 36 = age at menarche, 37 = age at menopause, 38 = gravidity, 39 = history of arthritis, 40 = age at first birth, 41 = duration of estrogen therapy, 42 = duration of combined postmenopausal hormone therapy, 43 = hysterectomy status, 44 = use of antihypertensive medication, 45 = history of coronary heart disease, 46 = use of cholesterol-lowering medication, 47 = history of ulcer, 48 = diabetes, 49 = hypertension, 50 = number of deliveries, 51 = menopausal status, 52 = household income, 53 = personal history of benign breast disease, 54 = study center, 55 = referent year, 56 = percentage Native American ancestry, 57 = breast procedure in the prior 3 years, 58 = other breast disease in the prior 3 years, 59 = neighborhood socioeconomic status, 60 = visit to a gynecologist in the prior year, 61 = practice of breast selfexamination, 62 = year of interview, 63 = number of physician visits 2 years before hospitalization, 64 = duration of oral contraceptive use, 65 = paracetamol, 66 = steroid, 67 = time since the last mammogram and duration and frequency of use, 68 = use of oral contraceptives, 69 = relative weight at age 12, 70 = questionnaire cycle, 71 = weight change since age 18 years, 72 = number of breast biopsies, 73 = history of surgical menopause, 74 = years of combined estrogen and progesterone hormone therapy, 75 = C-reactive protein level, 76 = all pain medication use, 77 = parity status before age 30

*AARP* AARP diet and health study, *BWHS* Black Women’s Health Study, *CHRI* Cancer Hospital and Richard J. Solove Research Institute, *GPRD* General Practitioners Research Database, *HPFS* Health Professionals follow-up study, *IWHS* Iowa Women’s Health Study, *MCC* the Spanish Multi-Case-control study, *NHS* nurses’ health study, *RAMQ* Re′gie de l’Assurance Maladie du Que’bec, *VITAL* the vitamins and lifestyle, *WEB* Western New York exposures and breast cancer study, *WHI* women’s health initiative

^a^Study deemed to be prone to immortal time bias

**Table 8:** Characteristics of included studies- ovarian cancer

Study source	Sex	Study period	Source of subjects	No of case	No of control/cohort size	Cancer site	Exposure assessment	Exposure Definition	Adjustment for covariates	Study quality
Case-control studies										
Peres LC [156], 2016, US	F	2010–2015	AACES	541	731	Epithelial ovarian cancer	Questionnaire	Use at least once a week or at least 5 days out of the month, at any point in their lifetime(regular)	1,4,5,10,18, 29,48,49,50,51,52,53,54,55	7
Baandrup L [157], 2015, Denmark	F	2000–2011	The Danish Cancer Registry	4103	58,706	Epithelial ovarian cancer	The Danish Prescription Registry	Use < 2 prescriptions (non-users)	1,5,7,25,29,43,50,52,55, 56,57,58,	8
Lo-Ciganic WH [158], 2012, US	F	2003–2008	HOPE study	625	1210	Ovarian cancer	Questionnaire	Use at least 2 tablets per week for 6 months or more(regular)	1, 5,7,10,11,30,39,42,49,50,55,59,60	7
Ammundsen HB [159], 2012, Denmark	F	1995–1999	Danish MALOVA study	756	1564	Ovarian cancer	Questionnaire	Use two times or more per week for more than 1 month	1,38,50,55,61	6
Pinheiro SP [160], 2010, US	F	1992–2003	New England Case-Control Study	1120	1160	Ovarian cancer	Questionnaire	Use at least twice aweek(regular)	1, 54	7
Wu AH [161], 2009, US	F	1998–2002	Population from Los Angeles County	582	668	Ovarian cancer	Questionnaire	Use aspirin medication 2 or more times a week for 1 month or longer	1,4,5,11,49, 50,51,55, 62	8
Wernli KJ [162], 2008, US	F	1998–2001	Population from Wisconsin and Massachusetts	400	2107	Ovarian cancer	Telephone interview	Use aspirin for more than 6 months and more than twice per week(ever use)	1,4,30, 43,49,51	7
Merritt MA [163], 2008, Australia	F	2002–2005	Australian Ovarian Cancer Study	1564	1502	Ovarian cancer	Self-administered questionnaires	Ever use of aspirin-not further defined	1,5,50,55	6
Schildkraut JM [164], 2006, US	F	1999–2003	North carolina ovarian cancer study	586	627	Ovarian cancer	In-person questionnaires	Use at least 3 month of use during the 5-year period(regular)	1,4,5,11,43,49, 50,53,60,63,64	7
Moysich KB [165], 2001, US	F	1982–1998	RPCI buffalo	547	1094	Ovarian cancer	Self-administered questionnaires	Use at least once a week for 6 consecutive months(regular)	1,4, 40,49,55, 65	6
Rosenberg L [166], 2000, US	F	1976–1998	Patients from hospital in Baltimore, Boston, New York, and Philadelphia	780	4623	Ovarian cancer	Questionnaire	Use at least 1 day per week for at least 6 months(regular)	1,30,59	7
Tavani A [167], 2000, US	F	1992–1999	Population from Italy	749	898	Ovarian cancer	Questionnaires	Use at least once a week for more than six consecutive months(regular)	1,5,10,37,50,54, 55,59	6
Cramer DW [168], 1998, US	F	1992–1997	Patients from hospital in eastern Massachusetts and all of New Hampshire	563	523	Ovarian cancer	In-person interviews	Use at least once a week for at least 6 months	1,5,9,46,54,55,66,67,68	8
Cohort studies										
Brasky TM [92], 2014, US	F	1998–2010	WHI	445	116,248	Ovarian cancer	Questionnaire	Use at both baseline and year 3 visits (consistent)	1,3,4,5, 6, 10,11,18,19,22,24,25,29,30,31,,32,33,34,35,36,37,38,39,40,41,42,43,44,45,46,47	9
SetiawanVW [169], 2012, Multinational	F	1993–2008	MEC	275	64,000	Ovarian cancer	Questionnaire	Use at least 2 times a week for 1 month or longer	1,11,25,36,50, 55	7
Murphy MA [170], 2012, US	F	1995–2006	AARP	438	96,710	Ovarian cancer	Mailed questionnaires	Use one or more pills per week(regular)	1,4,11,25,36,37,43,50,55,	7
Prizment AE [171], 2010, US	F	1992–2006	IWHS	157	21,694	Ovarian cancer	Questionnaire	Had ever taken aspirin- not further defined	1,10,25,45,55,69	9
Pinheiro SP [160], 2010, US	F	1992–2003	NHS and NHS-II cohorts	217	628	Ovarian cancer	Questionnaire	Use at least twice a week(regular)	1,25,51	7
Lacey JV [172], 2004, US	F	1979–1998	BCDDP	116	31,364	Ovarian cancer	Telephone interview and mailed questionnaires	Use at least once a week for 1 year(regular)	1,4,10,11,42,50,51,55	7
Friis S [62], 2003, Denmark^a^	F	1989–1997	Population from North Jutland County	34	29,470	Ovarian cancer	Prescription database	75–150 mg once daily(low-dose aspirin)	1,2	8
Akhmedkhanov A [173], 2001, US	F	1994–1996	The NYU Women’s Health Study	68	680	Epithelial ovarian cancer	Self-administered questionnaires	Use three or more times per week for at least 6 months	4,36,50, 55	8

1 = age, 2 = sex, 3 = smoking, 4 = family history, 5 = educational level, 6 = alcohol intake, 7 = chronic obstructive pulmonary disease or asthma, 8 = Fat distribution, 9 = religion, 10 = BMI, 11 = race, 12 = folate, 13 = Charlson comorbidity index, 14 = statin, 15 = metformin, 16 = ACE inhibitors, 17 = Angiotensin II receptor blockers, 18 = physical activity, 19 = fruit, vegetable and/or vitamin intake, 20 = history of colonoscopy, 21 = total energy intake, 22 = ever use of calcium supplements in the past 5 years, 23 = former health checkup, 24 = red meat, 25 = hormone replacement therapy, 26 = unique number of dispensing, 27 = unique number of hospitalizations in the year prior to start of follow up, 28 = mammogram in past 2 y, 29 = other NSAIDs, 30 = area (county/region), 31 = migraine, 32 = Nitro-vasodilator use, 33 = observational study enrollment, 34 = diet modification trial enrollment, 35 = screening for cancer, 36 = age at menarche, 37 = age at menopause, 38 = gravidity, 39 = history of arthritis, 40 = age at first birth, 41 = duration of estrogen therapy, 42 = duration of combined postmenopausal hormone therapy, 43 = hysterectomy status, 44 = use of antihypertensive medication, 45 = history of coronary heart disease, 46 = use of cholesterol-lowering medication, 47 = history of ulcer, 48 = income, 49 = tubal ligation, 50 = oral contraceptive use, 51 = menopausal status, 52 = endometriosis, 53 = pelvic inflammatory disease, 54 = study site, 55 = parity, 56 = infertility, 57 = diabetes mellitus, 58 = tubal sterilization, 59 = interview year, 60 = breastfeeding, 61 = duration of oral contraceptive use, 62 = talc use, 63 = months of pregnancy, 64 = severe menstrual cramping, 65 = presence of irregular menses, 66 = menstrual, headache, or arthritic pain, 67 = ibuprofen, 68 = paracetamol, 69 = partial oophorectomy

*AACES* the African American Cancer Epidemiology Study, *AARP* AARP Diet and Health Study, *BCDDP* the Breast Cancer Detection Demonstration Project, *HOPE* hormones and Ovarian cancer prediction study, *IWHS* Iowa Women’s Health Study, *MALOVA* Danish MALignant Ovarian cancer study, *MEC* multiethnic cohort study, *NHS* nurses’ health study, *NYU* New York University, *RPCI* the Roswell Park Cancer Institute, *WHI* women’s health initiative

^a^Study deemed to be prone to immortal time bias

**Table 9:** Characteristics of included studies- endometrial cancer

Study source	Sex	Study period	Source of subjects	No of case	No of control/cohort size	Cancer site	Exposure assessment	Exposure Definition	Adjustment for covariates	Study quality
Case-control studies										
Brons N [174], 2015, Denmark	F	2000–2009	Patients from Civil Registration System	5382	72,127	endometrial cancer	Prescription	Use ≥2 prescriptions on separate dates over the entire study period(ever users)	1,5,25,29,48,49,50,51	8
Neill AS [175], 2013, Australia	F	2005–2007	ANECS	1360	712	endometrial cancer	Telephone interview	Had ever taken aspirin- not further defined	1,3,10,25,36,48,50,52	7
Bosetti C [176], 2010, Italy	F	1992–2006	Population from Italy	442	676	Endometrial Cancer	Questionnaire	Use at least once a week for more than 6 months(regular)	1,5,10,25,36,48, 52,53,54,55	5
Fortuny J [177], 2009, US	F	2001–2005	The EDGE Study	469	467	endometrial cancer	Interview	Use aspirin for 6 months or longer	1,10	7
Bodelon C [178], 2009, US	F	2003–2005	Population from King, Pierce, and Snohomish counties	330	286	Endometrial Cancer	In-person interview	Use for more than 5 days per month for at least 6 months	1,7,10,25,30	6
Moysich KB [179], 2005, US	F	1982–1998	RPCI Institute	427	427	Endometrial Cancer	Questionnaire	Use at least once a week for 6 months (regular)	1,5,10,36,37,48	6
Cohort studies										
Brasky TM [92], 2014, US	F	1998–2010	WHI	865	85,351	Endometrial cancer	Questionnaire	Use at both baseline and year 3 visits (consistent)	1,3,4,5, 6, 10,11,18,19,22,24,25,29,30,31, 32,33,34,35,36,37,38,39,40,41,42,43,44,45,46,47	9
Brasky TM [180], 2013, US	F	2000–2010	VITAL Cohort	248	22,268	Endometrial Cancer	Mailed baseline questionnaire	Use≥4 days/week and ≥ 4 years(high use)	1,3,4,5,6,10,11,18,25,29,31,36.37,39,45,47,48, 50,57,58,59	7
SetiawanVW [169], 2012, Multinational	F	1993–2008	MEC	620	64,000	Endometrial cancer	Questionnaire	Use at least 2 times a week for 1 month or longer	1,3,10,11,25,36,48,52,.	7
Prizment AE [171], 2010, US	F	1992–2006	IWHS	311	21,694	Endometrial cancer	Questionnaire	Had ever taken aspirin- not further defined	1,6,10,25,36,37,50,52, 56	9
Danforth KN [181], 2009, US	F	1995–2003	AARP	576	72,524	Endometrial cancer	Mailed questionnaire	Had ever taken aspirin- not further defined	3,4,10,11,18,36,37,45,48,50,52,56	7
Viswanathan AN [182], 2008, US^a^	F	1980–2004	The NHS	436	82,971	Endometrial cancer	Medical record	Use at least 1 tablet per week or 1 day per week(current user)	4,10,18,25,37,40,60,61,62	6
Friis S [62], 2003, Denmark^a^	F	1989–1997	Population of North Jutland County	45	29,470	Endometrial cancer	Prescription database	75–150 mg once daily(low-dose aspirin)	1,2	8
Schreinemachers DM [63], 1994, US	F	1971–1987	The National Health and Examination Survey Ι	26	12,668	Endometrial cancer	Self reported	Use aspirin during the 30-day period before the interview	1,2	6

1 = age, 2 = sex, 3 = smoking, 4 = family history, 5 = educational level, 6 = alcohol intake, 7 = calendar year, 8 = Fat distribution, 9 = social status, 10 = BMI, 11 = race, 12 = folate, 13 = Charlson comorbidity index, 14 = statin, 15 = metformin, 16 = ACE inhibitors, 17 = Angiotensin II receptor blockers, 18 = physical activity, 19 = fruit, vegetable and/or vitamin intake, 20 = history of colonoscopy, 21 = total energy intake, 22 = ever use of calcium supplements in the past 5 years, 23 = former health checkup, 24 = red meat, 25 = hormone replacement therapy, 26 = unique number of dispensing, 27 = unique number of hospitalizations in the year prior to start of follow up, 28 = mammogram in past 2 y, 29 = other NSAIDs, 30 = area (county/region), 31 = migraine, 32 = Nitro-vasodilator use, 33 = observational study enrollment, 34 = diet modification trial enrollment, 35 = screening for cancer, 36 = age at menarche, 37 = age at menopause, 38 = gravidity, 39 = history of arthritis, 40 = age at first birth, 41 = duration of estrogen therapy, 42 = duration of combined postmenopausal hormone therapy, 43 = hysterectomy status, 44 = use of antihypertensive medication, 45 = history of coronary heart disease, 46 = use of cholesterol-lowering medication, 47 = history of ulcer, 48 = parity, 49 = obesity, 50 = diabetes, 51 = chronic obstructive pulmonary disease, 52 = oral contraceptive use, 53 = study center, 54 = period of interview, 55 = menopausal status, 56 = hypertension, 57 = years of oral contraceptive use, 58 = oophoerectomy, 59 = history of stroke, 60 = waist-hip ratio, 61 = intrauterine device use, 62 = height

*AARP* AARP diet and health study, *ANECS* Australian National Endometrial Cancer Study, *EDGE Study* estrogen, diet, genetics, and endometrial cancer, *IWHS* Iowa Women’s Health Study, *MEC* multiethnic cohort study, *NHS* nurses’ health study, *RPCI* the Roswell Park Cancer Institute, *VITAL* the vitamins and lifestyle, *WHI* women’s health initiative

^a^Study deemed to be prone to immortal time bias

**Table 10:** Characteristics of included studies- cervix uterus

Study source	Sex	Study period	Source of subjects	No of case	No of control/cohort size	Cancer site	Exposure assessment	Exposure Definition	Adjustment for covariates	Study quality
Case-control studies										
Friel G [183], 2015, US	F	1982–1998	RPCI	272	1072	Cervical Cancer	Questionnaire	Use at least once a week for 6 months(regular)	1,3,4,5, 6,7,8,9,10,11,12,13	7
Cohort studies										
Wilson JC [184], 2013, UK^a^	F	1995–2010	CPRD	724	3479	Cervical Cancer	Prescription database	Use of aspirin - not further defined	3,14,15,16,17,18,19,20,21	7
Friis S [62], 2003, Denmark^a^	F	1989–1997	Population from North Jutland County	15	29,470	Cervix uterus cancer	Prescription database	75–150 mg once daily(low-dose aspirin)	1,2	8
Schreinemachers DM [63], 1994, US	F	1971–1987	The National Health and Examination Survey Ι	29	12,668	Cervix uterus cancer	Self reported	Use aspirin during the 30-day period before the interview	1,2	6

1 = age, 2 = sex, 3 = smoking, 4 = spermicide contraceptive use, 5 = circulatory system disease, 6 = education, 7 = age at first pregnancy, 8 = menopausal status, 9 = genital tract disease, 10 = year survey completed,11 = blood and blood-forming organs disease, 12 = oral, 13 = barrier, 14 = HRT use, 15 = hormone contraceptive use, 16 = systemic steroids, 17 = DMARD use, 18 = history of cancer, 19 = years of follow-up, 20 = sexually transmitted infections, 21 = use of antiviral drugs

*CPRD* clinical practice research datalink, *RPCI* the Roswell Park Cancer Institute

^a^Study deemed to be prone to immortal time bias

**Table 11:** Characteristics of included studies- prostate cancer

Study source	Sex	Study period	Source of subjects	No of case	No of control/cohort size	Cancer site	Exposure assessment	Exposure Definition	Adjustment for covariates	Study quality
Case-control studies										
Iqbal U [47], 2017, China	M	2001–2011	The Taiwan NHI database	32,419	129,676	Prostate cancer	Prescription	Patients had aspirin prescribed at least for 2 months during the 3-year period before the initial cancer diagnosis	1,2,13,14, 15,16,17	7
Skriver C [185], 2016, Denmark	M	2000–2012	Danish nationwide registries	35,600	177,992	Prostate cancer	Prescription	Use aspirin ≥ 2 prescriptions redeemed on separate dates(ever use)	1, 4,14,28,30,36,37,38,,40,	8
Veitonmäki T [186], 2013, Finland	M	1995–2002	Finnish Cancer Registry	13,478	24,657	Prostate cancer	Prescription database	Ever use aspirin- not further defined	1,32	8
Murad AS [187], 2011, UK	M	2001–2008	ProtecT	1016	5043	Prostate cancer	Questionnaire	Ever use aspirin- not further defined	1,28,33,35	8
Salinas CA [188], 2010, US	M	2002–2005	SEER cancer registry	1000	942	Prostate cancer	Questionnaire	Use at least once per week for 3 months(ever use)	1,11,42	7
Harris RE [189], 2007, US	M	1999–2005	CHRI	24	39	Prostate cancer	Medical-record	At least two times per week for 2 years or more	1,3,5,6,10	5
Bosetti C [190], 2006, Italy	M	1991–2002	Population from the greater Milan area, the provinces of Pordenone, Gorizia, Latina and the urban area of Naples	1261	1131	Prostate cancer	Standard questionnaire	Use at least once a week for more than 6 months (regular)	1,4,5,34	5
Dasgupta K [191], 2006, Canada	M	1999–2002	RAMQ	2025	2150	Prostate cancer	Prescription database	Did not receive any prescription for aspirin (nonuser)	1,43	6
Liu X [192], 2006, US	M	2001–2004	Population from Cleveland, Ohio	471	468	Prostate cancer	Personal interview	Use at least twice a week for more than a month(any use)	1,11,44	5
Menezes RJ [193], 2006, US	M	1982–1998	RPCI	1029	1029	Prostate cancer	Questionnaire	Use at least once a week for at least 6 months (regular)	1,5,10	5
Perron L [194], 2003, Canada	M	1993–1995	RAMQ	2221	11,105	Prostate cancer	Prescription database	Ever use aspirin- not further defined	1,50	6
Norrish AE [195], 1998, New Zealand	M	1996–1997	Auckland Prostate Study	317	480	Prostate cancer	Questionnaire	At least once per week(regular)	1,50,51,52,53	7
Neugut AI [80], 1998, US	M	1989–1992	Columbia-Prebyterian Medical Center	319	189	Prostate cancer	Medical record	Use aspirin-not further defined	1,4,5	6
Cohort studies										
Cao Y [8], 2016, US	M	1980–2010 1986–2012	NHS and HPFS	1019	135,965	Prostate cancer	Questionnaire	Use at least 2 times per week(regular)	3,5,6,7,8,9,10,11,12,18,19,20,21,22,23,24,25,70	9
Lapi F [196], 2016, Italy^a^	M	2002–2013	HSD	187	13,453	Prostate Cancer	Prescription database	Use low-dose aspirin-not further defined	1,3,6,9,13,14,16,28,38,54,55,56,57	8
Nordström T [197], 2015, Sweden^a^	M	2007–2012	Population from Stockholm County, Sweden	8430	204,241	Prostate cancer	Swedish Prescribed Drug Register	Any dispensed prescription of the drug within 2 years before biopsy	1,4,13,14, 58,59,60	5
Hollestein LM [91], 2014, Netherlands^a^	M	1998–2010	PHARMO and the Eindhoven Cancer Registry	882	53,679	Prostate cancer	Prescription database	Low dose aspirin (≤ 100 mg daily)- not further defined	1,2,26,27	8
Shebl FM [198], 2012, US	M	1993–2001	PLCO	3573	29,450	Prostate cancer	Questionnaire	Regular use aspirin-not further defined	5,11,34, 42,62	7
Mahmud SM [199], 2011, Canada^a^	M	1985–2000	Saskatchewan Ministry of Health (SH) databases and the Saskatchewan Cancer Registry (SCR).	9007	35,891	Prostate cancer	Prescription database	Had a participant ever filled a prescription of aspirin in the index class at any time during his exposure history	28,41,42	6
Brasky TM [200], 2010, US	M	2000–2007	VITAL Cohort	1547	34,132	Prostate cancer	Questionnaire	Use aspirin ≥1 day/ week for ≥ 1 year(regular)	1,4,5,9,10,11,19,30,55, 65,66,67	5
Siemes C [96], 2008, Netherland	M	1992–2004	The Rotterdam Study	216	7621	Prostate cancer	Questionnaire and prescriptions	The absence of a prescription for any non-aspirin or aspirin NSAID(no use)	1,3,10,61	8
Jacobs EJ [98], 2007, US	M	1992–2003	Cancer Prevention Study II Nutrition Cohort	5539	69,810	Prostate cancer	Questionnaire	Use at least 30 “times” per month(daily use of adult-strength)	1,3,4,9,10,11,18, 20,28,29,30,31	8
Platz EA [201], 2005, US	M	1980–2004	BLSA	141	9748	Prostate cancer	Self-reported	Had ever taken aspirin-not further defined	1,28,45,68	7
García Rodríguez LA [44], 2004, UK^a^	M	1995–2001	GPRD	2096	9579	Prostate cancer	Prescription database	No use of aspirin at any time before the index date(nonuser)	1,45,46,47,48,49	8
Friis S [62], 2003, Denmark^a^	M	1989–1997	Population of North Jutland County	196	29,470	Prostate cancer	Prescription database	75–150 mg once daily(low-dose aspirin)	1,2	8
Habel LA [202], 2002, US	M	1964–1973	The Kaiser Permanente Medical Care Program in Northern California	2574	90,100	Prostate cancer	Questionnaire	Use more than six aspirin per days	1,4,11,69	6
Schreinemachers DM [63], 1994, US	M	1971–1987	The National Health and Examination Survey Ι	123	12,668	Prostate cancer	Self reported	Use aspirin during the 30-day period before the interview	1,2	6
Paganini-Hill A [103], 1989, US	M	1981–1988	Population from Leisure World, Laguna Hills, US	149	13,870	Prostate cancer	Questionnaire	Aspirin use: none,<daily, daily	2	4

1 = age, 2 = sex, 3 = smoking, 4 = education level, 5 = family history, 6 = alcohol intake, 7 = height, 8 = Alternate Healthy Eating Index-2010, 9 = PSA test in past 2 y, 10 = BMI, 11 = race, 12 = folate, 13 = Charlson comorbidity index, 14 = statin, 15 = metformin, 16 = ACE inhibitors, 17 = Angiotensin II receptor blockers, 18 = physical activity, 19 = fruit, vegetable and/or vitamin intake, 20 = history of colonoscopy, 21 = total energy intake, 22 = ever use of calcium supplements in the past 5 years, 23 = former health checkup, 24 = mammogram in past 2 y, 25 = hormone replacement therapy, 26 = unique number of dispensing, 27 = unique number of hospitalizations in the year prior to start of follow up, 28 = other NSAIDs, 29 = history of heart attack, 30 = diabetes, 31 = hypertension, 32 = simultaneous use of other medications (cholesterol lowering drugs, anti-diabetic drugs, antihypertensive drugs and benign prostatic hyperplasia medication), 33 = the primary care centres from which they were recruited, 34 = study center, 35 = any paracetamol use, 36 = residence (by design), 37 = use of high-dose aspirin, 38 = 5-alpha reductase inhibitors, 39 = income, 40 = selected cardiovascular drugs, and antidepressants or neuroleptics, 41 = ever visited a urologist 1–11 years prior, 42 = SCREENED and volume of family physician visits in the 5 years prior to the index date, 43 = finasteride, 44 = medical institution, 45 = calendar year, 46 = prior BPH history, 47 = number of visits to general practitioners, 48 = referrals, 49 = hospitalizations, 50 = recent medical contacts, 51 = socio-economic status, 52 = total polyunsaturated fat consumption, 53 = a-linolenic acid and ratio of dietary n-6:long-chain n-3 polyunsaturated fatty acids, 54 = presence of obesity, 55 = benign prostatic hypertrophy, 56 = alpha-adrenoreceptor antagonists, 57 = immunosuppressive drugs, 58 = natural log-transformed prostate specific antigen (PSA) concentration, 59 = PSA quotient, 60 = use of antidiabetic medication, 61 = C-reactive protein level, 62 = ibuprofen use, 63 = osteoarthritis, 64 = rheumatoid arthritis,65 = enlarged prostate, 66 = coronary artery disease, 67 = chronic joint pain, chronic headaches, and migraines, 68 = acetaminophen, 69 = and number of health checkups, 70 = red meat

*BLSA* Baltimore Longitudinal study of Aging, *CHRI* Cancer Hospital and Richard J. Solove Research Institute, *GPRD* general practitioners research database, *HPFS* Health Professionals follow-up study, *HSD* health search IMS health longitudinal patient database, *NHS* nurses’ health study, *PLCO* prostate, lung, colorectal and ovarian cancer screening trial, *ProtecT* prostate testing for cancer and Treatment, *RAMQ* Re′gie de l’Assurance Maladie du Que’bec, *RPCI* the Roswell Park Cancer Institute, *SEER* surveillance, epidemiology and end results, *VITAL* the vitamins and lifestyle

^a^Study deemed to be prone to immortal time bias

**Table 12:** Characteristics of included studies- renal cancer

Study source	Sex	Study period	Source of subjects	No of case	No of control/cohort size	Cancer site	Exposure assessment	Exposure Definition	Adjustment for covariates	Study quality
Case-control studies										
Karami S [203], 2016, US	M/F	2002–2007	US Kidney Cancer Study	1187	1204	Renal-cell cancer	Questionnaires	Use at least once a week for 3 months or longer, at least 2 years prior to the interview	1,2,3,4,5,10,11, 27,51,52	8
Tavani A [204], 2010, Italy	M/F	1992–2004	Population from Italian areas	755	1297	Renal-cell cancer	Questionnaires	Use at least once a week for more than 6 months(regular)	1,2,3,5,6,7,27,55,56	7
Gago-Dominguez M [205], 1999, US	M/F	1986–1994	Patients from Los Angeles County	1204	1204	Renal-cell cancer	Questionnaires	Had ever taken the drug 20 or more times	3,5,10,27,57	6
Chow WH [206], 1994, US	M/F	1988–1990	Population from Minnesota	440	691	Renal-cell cancer	Interviewer	Use at least 2 or more times per week for 1 month or longer (regular)	1,3,10	6
McCredie M [207], 1993, Austrilia	M/F	1989–1990	The NSW Central Cancer Registry	489	523	Renal-cell cancer	Questionnaires	Had ever taken the drug 20 or more times	1,2,3,50,58,	7
McCredie M [208], 1988, Austrilia	M/F	1977–1982	New South Wales Central Cancer Registry	360	985	Kidney cancer	Questionnaires	Had taken a total of more than 0.1 kg	1,2,3,44,59,60,61	6
Cohort studies										
Karami S [203], 2016, US	M/F	2002–2007	Prostate, Lung, Colorectal and Ovarian Cancer Screening Trial	135	98,807	Renal cell carcinoma	Questionnaires	Use at least once per week	1,3,5,10,11,27,51	7
Brasky TM [92], 2014, US	F	1998–2010	WHI	329	141,880	Kidney cancer	Questionnaires	Use at both baseline and year 3 visits (consistent)	1,3,4,5, 6,10,11,17,18,19,25,26,28,29,30,31,32,34,35,36,37,38,39,40,41,42,43,44,45,46,47,48	9
Liu W [209], 2013, US	M/F	1996–2006	AARP	884	298,468	Renal cell carcinoma	Questionnaires	Any use of aspirin	1,2,3,4,5,6,7,10,11,18,27,53,54	7
Cho E [210], 2011, US	F	1986–2006	NHS	153	77,525	Renal cell carcinoma	Questionnaires	Use aspirin ≥2 times/week(regular)	1,3,6,10,18,19,27,39	7
	M	1990–2006	HPFS	180	49,403	Renal cell carcinoma	Questionnaires	Use aspirin ≥2 times/week(regular)	1,3,6,10,18,19,27	9
Jacobs EJ [98], 2007, US	M/F	1992–2003	Cancer Prevention Study II Nutrition Cohort	365	146,113	Kidney cancer	Questionnaires	Use at least 30 “times” per month(daily use of adult-strength)	1,2,3,5,7,10,11, 15,16,17,18,20,25, 27,45	8
Friis S [62], 2003, Denmark^a^	M/F	1989–1997	Population from North Jutland County	67	29,470	Kidney cancer	Prescription database	75–150 mg once daily(low-dose aspirin)	1,2	8
Schreinemachers DM [63], 1994, US	M/F	1971–1987	The National Health and Examination Survey Ι	32	12,668	Kidney cancer	Self reported	Use aspirin during the 30-day period before the interview	1,2	6
Paganini-Hill A [103], 1989, US	M/F	1981–1988	Population from Leisure World, Laguna Hills, US	25	13,870	Kidney cancer	Questionnaires	Aspirin use: none,<daily, daily	2	4

1 = age, 2 = sex, 3 = smoking, 4 = family history, 5 = educational level, 6 = alcohol intake, 7 = diabetes, 8 = fat distribution, 9 = social status, 10 = BMI,11 = race, 12 = folate, 13 = height, 14 = Alternate Healthy Eating Index-2010, 15 = PSA test in past 2 y, 16 = mammogram in past 2 y, 17 = hormone replacement therapy, 18 = physical activity, 19 = fruit, vegetable and/or vitamin intake, 20 = history of colonoscopy, 21 = total energy intake, 22 = ever use of calcium supplements in the past 5 years, 23 = former health checkup, 24 = red meat, 25 = other NSAIDs, 26 = area (county/region), 27 = hypertension, 28 = migraine, 29 = ever use of calcium supplements in the past 5 years, 30 = red meat, 31 = Nitro-vasodilator use, 32 = height, 33 = unique number of hospitalizations in the year prior to start of follow up, 34 = observational study enrollment, 35 = diet modification trial enrollment, 36 = screening for cancer, 37 = age at menarche, 38 = age at menopause, 39 = gravidity, 40 = age atfirst birth, 41 = duration of estrogen therapy, 42 = duration of combined postmenopausal hormone therapy, 43 = hysterectomy status, 44 = use of antihypertensive medication, 45 = history of coronary heart disease, 46 = use of cholesterol-lowering medication, 47 = history of arthritis, 48 = history of ulcer, 49 = method of interview, 50 = obesity, 51 = center, 52 = dialysis treatment, 53 = marital status, 54 = total dietary fiber, 55 = study center, 56 = year of interview, 57 = regular use of amphetamines, 58 = method of interview, 59 = phenacetin, 60 = paracetamol, 61 = urological disease

*AARP* AARP diet and health study, *HPFS* Health Professionals follow-up study, *NHS* nurses’ health study, *WHI* women’s health initiative

^a^Study deemed to be prone to immortal time bias

**Table 13:** Characteristics of included studies- renal pelvis and ureter

Study source	Sex	Study period	Source of subjects	No of case	No of control/cohort size	Cancer site	Exposure assessment	Exposure Definition	Adjustment for covariates	Study quality
Case-control studies										
Linet MS [211], 1995, US	M/F	1983–1986	Cancer registries in New Jersey,Iowa and Los Angeles	418	405	Renal pelvis and ureter cancer	Questionnaire	Use 2 or more doses per week for at least 1 month or longer(regular)	1,2,3,7	8
Mccredie M [207], 1993, Australia	M/F	1989–1990	The NSW Central Cancer Registry	147	523	Renal pelvis cancer	Questionnaire	Had ever taken the drug 20 or more times	1,2,3,5,8	7
Ross RK [212], 1989, US	M/F	1978–1982	The Cancer Surveillance Program in Los Angeles County	187	187	Renal pelvis and ureter cancer	Telephone interviews	Use aspirin for more than 30 days in a single year	1,2,6	8
Jensen OM [213], 1989, Denmark	M/F	1979–1982	Patients in hospitals of Copenhagen	90	251	Renal pelvis and ureter cancer	Face-to-face interviews	Use of aspirin - not further defined	1,2,4	7

1 = age, 2 = sex, 3 = smoking, 4 = hospital, 5 = educational level, 6 = race, 7 = geographic site, 8 = method of interview

**Table 14:** Characteristics of included studies- bladder cancer

Study source	Sex	Study period	Source of subjects	No of case	No of control/cohort size	Cancer site	Exposure assessment	Exposure Definition	Adjustment for covariates	Study quality
Case-control studies										
Baris D [214], 2013, US	M/F	2001–2004	Population from Maine, Vermont and New Hampshire	783	890	Bladder cancer	Self-reported	Use at least 20 times(any)	1,2,3,11,26,51	6
Fortuny J [215], 2007, US	M/F	1998–2001	The New Hampshire State Department of Health and Human Services’ rapid reporting Cancer Registry	456	369	Bladder cancer	Interview	Use at least four times a week for 1 month or longer prior to the reference date	1,2,3,25	7
Fortuny J [216], 2006,Spain	M/F	1997–2000	Patients from five regions in Spain (Barcelona, Valle’s/Bages, Alacant, Tenerife, and Asturias)	907	965	Bladder cancer	Self-reported	Use twice or more weekly for ≥ 1 month (regular)	1,2,3,25,26,52,53	8
Castelao JE [217], 2000, US	M/F	1987–1996	SEER cancer registry	1514	1514	Bladder cancer	Questionnaire	Use at least 20 times(any)	3,5,53,54,55,56, 57,58,59,60	7
Steineck G [218], 1995, Sweden	M/F	1985–1987	Population from the County of Stockholm	325	393	Bladder cancer	Questionnaire	Had ever taken aspirin-not further defined	1,2,3,55,56,61,62,63	5
Cohort studies										
Brasky TM [92], 2014, US	F	1998–2010	WHI	175	142,330	Bladder cancer	Questionnaire	Use at both baseline and year 3 visits (consistent)	1,3,4,5,6,10,11,17,18,19,25,26,,28,29,30,31,32,34,35,36,37,38,39,40,41,42,43,44,45,46,47,48	9
Shih C [219], 2013, US	M/F	2000–2010	The VITAL cohort	344	77,048	Bladder cancer	Questionnaire	Use at least once per week, for at least 1 year	1,2,3,4,5,11,49	8
Daugherty SE [220], 2011, US	M/F	1995–1996	AARP	1660	334,908	Bladder cancer	Questionnaire	Use aspirin ≥ 2times/week (regular)	3,10,11,25,27	7
		1993–2001	PLCO Cancer Screening	704	154,952	Bladder cancer	Questionnaire	Use aspirin ≥ 2times/week (regular)	3,10,11,25,27	7
		1994–1998	The USRT Study	97	90,972	Bladder cancer	Questionnaire	Use aspirin ≥ 2times/week (regular)	3,10,11,25,27	7
Genkinger JM [221], 2007, US	M	1986–2004	HPFS	392	49,448	Bladder cancer	Questionnaire	Use 2 or more times per week(regular)	1,3,26,50	9
Jacobs EJ [98], 2007, US	M/F	1992–2003	Cancer Prevention Study II Nutrition Cohort	867	146,113	Bladder cancer	Questionnaire	Use at least 30 “times” per month(daily use of adult-strength)	1,2,3,5,7,10,11, 15,16,17,18,22, 25,45, 63	8
Friis S [62], 2003, Denmark^a^	M/F	1989–1997	Population of North Jutland County	161	29,470	Bladder cancer	Prescription database	75–150 mg once daily(low-dose aspirin)	1,2	8
Schreinemachers DM [63], 1994, US	M/F	1971–1987	The National Health and Examination Survey Ι	35	12,668	Bladder cancer	Self reported	Use aspirin during the 30-day period before the interview	1,2	6
Paganini-Hill A [103], 1989, US	M/F	1981–1988	Population from Leisure World, Laguna Hills, US	96	13,870	Bladder cancer	Questionnaire	Aspirin use: none,<daily, daily	2	4

1 = age, 2 = sex, 3 = smoking, 4 = family history, 5 = educational level, 6 = alcohol intake, 7 = history of colorectal endoscopy, 8 = Fat distribution, 9 = social status, 10 = BMI,11 = race, 12 = folate, 13 = height, 14 = Alternate Healthy Eating Index-2010, 15 = PSA test in past 2 y, 16 = mammogram in past 2 y, 17 = hormone replacement therapy, 18 = physical activity, 19 = fruit, vegetable and/or vitamin intake, 20 = history of colonoscopy, 21 = total energy intake, 22 = diabetes, 23 = former health checkup, 24 = red meat, 25 = other NSAIDs, 26 = area (county/region), 27 = study, 28 = migraine, 29 = ever use of calcium supplements in the past 5 years, 30 = red meat, 31 = Nitro-vasodilator use, 32 = height, 33 = unique number of hospitalizations in the year prior to start of follow up, 34 = observational study enrollment, 35 = diet modification trial enrollment, 36 = screening for cancer, 37 = age at menarche, 38 = age at menopause, 39 = gravidity, 40 = age atfirst birth, 41 = duration of estrogen therapy, 42 = duration of combined postmenopausal hormone therapy, 43 = hysterectomy status, 44 = use of antihypertensive medication, 45 = history of coronary heart disease, 46 = use of cholesterol-lowering medication, 47 = history of arthritis, 48 = history of ulcer, 49 = indications for NSAID use, 50 = fluid intake, 51 = hispanic status, 52 = Metamizol, 53 = Acetic acids, 54 = number of years employed as hairdresser/barber, 55 = use of phenacetin, 56 = acetaminophen, 57 = Other salicylic acids, 58 = Propionic acids, 59 = Oxicam,60 = Pyrazolon derivatives, 61 = Dextropropoxyphene, 62 = Phenazon, 63 = Other analgesics (codeine, chlormezanone, caffeine), 63 = hypertension

*AARP* AARP diet and health study, *HPFS* Health Professionals follow-up study, *PLCO* prostate, lung, colorectal and ovarian cancer screening trial, *SEER* surveillance, epidemiology and end results, *USRT* United State Radiologic Technologist Study, *VITAL* the vitamins and lifestyle, *WHI* women’s health initiative

^a^Study deemed to be prone to immortal time bias

**Table 15:** Characteristics of included studies- brain tumor

Study source	Sex	Study period	Source of subjects	No of case	No of control/cohort size	Cancer site	Exposure assessment	Exposure Definition	Adjustment for covariates	Study quality
Case-control studies										
Egan KM [41], 2016, US	M/F	2004–2012	Population in Southeastern US	1433	1296	Brain tumor	Interview	Use at least twice a week for 12 consecutive months (regular)	1,2,5,7,8	6
Gaist D [222], 2013, Denmark	M/F	2000–2009	Danish Cancer Registry, Civil Registration System, National Prescription Registry, Danish National Registry of Patients, and Danisheducation and fertility registries within Statistics Denmark	2688	18,848	Glioma	National Prescription Registry	Use aspirin as a ‘low’ (≤ 100 mg) or ‘high’ (150 mg) daily dose of low-dose aspirin	5,10,13,14,15,16,17,18	7
Ferris J [223], 2012, US	M/F	2007–2010	CUMC	236	230	Glioma	Questionnaire	Use at least twice a weekfor 6 months or longer(ever use)	1,2,7,9,11,12,13	7
			The UCSF	281	170	Glioma	Questionnaire	Use at leasttwice a week for 6 months or longer(ever use)	1,2,7,9,11,12,13	
Cohort studies										
Bannon FJ [224], 2013, UK^a^	M/F	1987–2009	UK Clinical Practice Research Datalink(CPRD)	5052	42,678	Brain tumor	Prescription database	Had ever taken aspirin- not further defined	1,2,8	7
Daugherty SE [225], 2011, US	M/F	1996–2006	AARP	605	302,767	Glioma	Questionnaire	Use aspirin ≥ 2 times/wk.(regular)	1,2,7,19	7
Friis S [62], 2003, Denmark^a^	M/F	1989–1997	Population from North Jutland County	70	29,470	Brain tumor	Prescription database	75–150 mg once daily(low-dose aspirin)	1,2	8

1 = age, 2 = sex, 3 = smoking, 4 = family history, 5 = educational level, 6 = alcohol intake, 7 = race, 8 = state of residence, 9 = center, 10 = anti-asthma medications, 11 = individual NSAIDs, 12 = acetaminophen, 13 = statins, 14 = diabetes, 15 = stroke, 16 = allergy, 17 = asthma, 18 = antihistamines, 19 = history of heart disease using age as time metric

*AARP* AARP diet and health study, *CPRD* clinical practice research datalink, *CUMC* Columbia University Medical Center, *UCSF* University of California San Francisco

^a^Study deemed to be prone to immortal time bias

**Table 16:** Characteristics of included studies- head and neck cancers

Study source	Sex	Study period	Source of subjects	No of case	No of control/cohort size	Cancer site	Exposure assessment	Exposure Definition	Adjustment for covariates	Study quality
Case-control studies										
Di Maso M [226], 2015, Italy	M/F	1992–2008	Population from Aviano, Pordenone and the greater Milan area in northern Italy	198	596	Nasopharyngeal cancer	Questionnaire	Use at least one aspirin a week for at least 6 months(regular)	1,2,3,5,11,12,13	6
Becker C [227], 2015, UK	M/F	1995–2013	CPRD	2745	16,470	Head and neck cancer	Prescription database	Use aspirin ≥1 Prescription	3,6,8,10	7
Macfarlane TV [228], 2012, Europe	M/F		ARCAGE	1779	1993	Head and neck cancer	Questionnaire	Use at least once a weekfor a year(regular)	1,2,3,5,6,10,18	7
Ahmadi N [229], 2010, US	M/F	2003–2007	Patients from the Lombardi Comprehensive Cancer Center, at GUMC	25	25	Head and neck cancer	Questionnaire	Daily use of aspirin	5,19	5
Jayaprakash V [230], 2006, US	M/F	1982–1998	RPCI	529	529	Head and neck cancer	Questionnaire	Had ever taken aspirin before the onset of the present illness	1,2,3,6	7
Rosenquist K [231], 2005, Sweden	M/F	2000–2004	Population from the Southern healthcare region of Sweden	132	320	Oral and oropharyngeal squamous cell carcinoma	Interview	Had ever taken aspirin-not further defined	3,6	6
Bosetti C [232], 2003, Italy	M/F	1992–2000	Population from Italy	740	1779	Oral and pharyngeal, laryngeal cancer	Questionnaire	Use at least once a week for more than 6 months	1,2,3,5,6,11	6
Cohort studies										
Macfarlane TV [69], 2014, UK^a^	M/F	1996–2010	PCCIU database	1195	3580	Head and neck cancer	Prescription database	Had at least one Prescription (users)	1,2,8,14,15,16,17	7
Wilson JC [233], 2013, US	M/F	1993–2001	PLCO	316	142,034	Head and neck cancer	Questionnaire	Use aspirin regularly -not further defined	1,2,3,10	7
Friis S [62], 2003, Denmark^a^	M/F	1989–1997	Population from North Jutland County	68	29,470	Head and neck cancer	Prescription database	75–150 mg once daily(low-dose aspirin)	1,2	8

1 = age, 2 = sex, 3 = smoking, 4 = family history, 5 = educational level, 6 = alcohol intake, 7 = race, 8 = other NSAIDs, 9 = social status, 10 = BMI, 11 = area of residence, 12 = period of interview, 13 = occupation, 14 = deprivation, 15 = CHD, 16 = stroke, 17 = COX-2 inhibitors, 18 = fruit consumption, 19 = marital status

*ARCAGE* the alcohol-related cancers and genetic susceptibility, *CPRD* clinical practice research datalink, *GUMC* Georgetown University Medical School, *PCCIU* primary care clinical informatics unit database, *PLCO* prostate, lung, colorectal and ovarian cancer screening trial, *RPCI* the Roswell Park Cancer Institute

^a^Study deemed to be prone to immortal time bias

**Table 17:** Characteristics of included studies- thyroid cancer

Study source	Sex	Study period	Source of subjects	No of case	No of control/cohort size	Cancer site	Exposure assessment	Exposure Definition	Adjustment for covariates	Study quality
Cohort studies										
Patel D [234], 2015, US	M/F	1993–2001	AARP	292	269,553	Thyroid cancer	Questionnaires	Use aspirin ≤ 2 times/Week(no regular use)	2,3,6,8,11	7
			PLCO	56	58,433	Thyroid cancer	Questionnaires	Use aspirin ≤ 2 times/Week(no regular use)	2,3,6,8,11	6
			U.S. Radiologic Technologists Study	133	60,591	Thyroid cancer	Questionnaires	Use aspirin ≤ 2 times/Week(no regular use)	2,3,6,8,11	6
Brasky TM [92], 2014, US	F	1998–2010	WHI	229	142,330	Thyroid cancer	Questionnaires	Use at both baseline and year 3 visits (consistent)	1,3,4,5,6,7,9,10,11,12,13,14,15,16,17,18,19,22,24,25,26,27,28,29,30,31,32,33,34,35,36,37	9

1 = age, 2 = sex, 3 = smoking, 4 = education level, 5 = family history, 6 = alcohol intake, 7 = height, 8 = weight, 9 = history of ulcer, 10 = BMI, 11 = race, 12 = duration of estrogen therapy, 13 = duration of combined postmenopausal hormone therapy, 14 = hysterectomy status, 15 = use of antihypertensive medication, 16 = history of coronary heart disease, 17 = use of cholesterol-lowering medication, 18 = physical activity, 19 = fruit, vegetable and/or vitamin intake, 20 = history of colonoscopy, 21 = total energy intake, 22 = ever use of calcium supplements in the past 5 years, 23 = former health checkup, 24 = red meat, 25 = hormone replacement therapy, 26 = gravidity, 27 = history of arthritis, 28 = age at first birth, 29 = other NSAIDs, 30 = area (county/region), 31 = migraine, 32 = Nitro-vasodilator use, 33 = observational study enrollment, 34 = diet modification trial enrollment, 35 = screening for cancer, 36 = age at menarche, 37 = age at menopause

*AARP* AARP diet and health study, *PLCO* prostate, lung, colorectal and ovarian cancer screening trial, *WHI* women’s health initiative

**Table 18:** Characteristics of included studies- skin cancer

Study source	Sex	Study period	Source of subjects	No of case	No of control/cohort size	Cancer site	Exposure assessment	Exposure Definition	Adjustment for covariates	Study quality
Case-control studies										
Reinau D [235], 2015, UK	M/F	1995–2013	GPRD	73,262	96,854	Skin cancer	Prescription database	Last prescription ≤ 1 year before the index date(current user)	3,6,10, 29,43,47,48,49,50,51,52	8
Johannesdottir SA [236], 2012, Denmark	M/F	1991–2009	Population from northern Denmark	18,532	178,655	Skin cancer	Prescription records	Redeemed > 2 prescriptions during the entire study period	1,2,20,44,45,46	8
Torti DC [237], 2011, US	M/F	1997–2000	Population from New Hampshire and bordering regions	1022	1484	Skin cancer	Interview	Use at least four times a week for at least 1 month	1,2,3,53,54,55	8
Curiel-Lewandrowski C [238], 2011, US	M/F	2004–2007	Dana Farber Harvard Cancer Center Institutions and Dermatology Associates of Concord, Boston(USA)	400	600	Cutaneous melanoma	Telephone interview	Use at least once weekly within a year preceding the interview (current user)	56	8
Jeter JM [239], 2011, US	M/F	2000–2003	The GEM study	327	119	Melanoma	Self-reported	Daily basis for at least 3 months	1,2,4,53,57	6
Asgari MM [240], 2010, US	M/F	1994–2004	KPNC	415	415	Cutaneous squamous sell sarcinoma	Questionnaire	Use at least once a week for at least 1 year(regular)	3,4,5,30,53,56,58,59,60,61,62,63,64,65,66,67,68	8
Cohort studies										
Hollestein LM [91], 2014, Netherlands^a^	M/F	1998–2010	PHARMO and the Eindhoven Cancer Registry	2363	109,276	Skin cancer	Prescription database	Low dose aspirin (≤ 100 mg daily)- not further defined	1,2,11,12	8
Wysong A [241], 2014, US	F	1993–1998	WHI	7652	54,728	Non-melanoma skin cancer	Questionnaire	Use ≥ 2 times/week for at least 2 weeks(regular)	1,3,5,7,10,14,15,19,21,29,43,69,70,71,72,73,74	6
Brasky TM [92], 2014, US	F	1998–2010	WHI	585	142,330	Melanoma	Self-administered questionnaires	Use at both baseline and year 3 visits (consistent)	1,3,4,5,6,10,13,14,15,16,17,18,19,20,21,22,23,24,25,26,27,28,29,30,31,32,33,34,35,36,37	9
Jeter JM [242], 2012, US	F	1980–2008	NHS	17,074	92,125	Skin cancer	Questionnaire	Use at least 1–2 tablets/week or 1 day/week of regular use at any lifetime(current user)	1,3,4,7,10,14,15,54,57,75,76,77,78,79	7
Cahoon EK [243], 2012, US	M/F	1994–1998 2003–2005	United States Radiologic Technologists study	2215	58,213	Basal cell carcinoma	Questionnaire	Use at least 1 days per month in the past year	1,2,80	8
Asgari MM [244], 2008, US	M/F	2000–2005	The VITAL cohort	216	39,909	Melanoma	Questionnaire	Use at least once a week for a year in the 10-year period before baseline(ever use)	1,2,4,5,7,15,29, 30,56,59,69,73,81,82,83	8
Jacobs EJ [98], 2007, US	M/F	1992–2003	Cancer Prevention Study II Nutrition Cohort	1049	146,113	Melanoma	Questionnaire	Use at least 30 “times” per month(daily use of adult-strength)	1,2,3,5,10,13,14,18,19,36,38,39,40,41,42	8
Schreinemachers DM [63], 1994, US	M/F	1971–1987	The National Health and Examination Survey Ι	69	12,668	Melanoma	Self reported	Use aspirin during the 30-day period before the interview	1,2	6

1 = age, 2 = sex, 3 = smoking, 4 = family history, 5 = educational level, 6 = alcohol intake, 7 = skin reaction to the sun, 8 = Fat distribution, 9 = social status, 10 = BMI, 11 = unique number of dispensing, 12 = unique number of hospitalizations in the year prior to start of follow up, 13 = race, 14 = physical activity, 15 = fruit, vegetable and/or vitamin intake, 16 = ever use of calcium supplements in the past 5 years, 17 = red meat, 18 = hormone replacement therapy, 19 = other NSAIDs, 20 = area (county/region), 21 = migraine, 22 = Nitro-vasodilator use, 23 = observational study enrollment, 24 = diet modification trial enrollment, 25 = screening for cancer, 26 = age at menarche, 27 = age at menopause, 28 = gravidity, 29 = history of arthritis, 30 = history of ulcer, 31 = age at first birth, 32 = duration of estrogen therapy, 33 = duration of combined postmenopausal hormone therapy, 34 = hysterectomy status, 35 = use of antihypertensive medication, 36 = history of coronary heart disease, 37 = use of cholesterol-lowering medication, 38 = mammography, 39 = history of colorectal endoscopy, 40 = history of PSA testing, 41 = diabetes, 42 = hypertension,43 = the number of general practitioner visits in the year before the index date, 44 = use of systemic glucocorticoids, cytostatic or immunosuppressive medication, 45 = drugs with pigmenting adverse effects, 46 = Charlson comorbidity index, 47 = photosensitising or phototoxic drugs,48 = inflammatory bowel disease, 49 = ischemic stroke/ transient ischemic attack, 50 = ischemic heart disease, 51 = psoriasis, 52 = systemic glucocorticoids and other immunosuppressants, 53 = skin type, 54 = lifelong number of painful sunburns, 55 = lifelong cumulative number of hours of sun exposure, 56 = number of sunburns of children, 57 = number of moles, 58 = eye color, 59 = natural hair color, 60 = exposure to industrial chemicals, 61 = history of freckling, 62 = outdoor sun exposure, 63 = occupational sun exposure, 64 = tanning bed use, 65 = history of high-risk exposures such as UV light, 66 = burn scar, 67 = radiation treatment, 68 = arsenic exposure, 69 = personal history of nonmelanoma skin cancer, 70 = personal history of melanoma, 71 = current and childhood summer sun exposure, 72 = sunscreen use, 73 = history of cardiovascular disease, 74 = regional solar radiation (Langleys), 75 = menopausal status and use of postmenopausal hormones, 76 = questionnaire cycle, 77 = ability to tan, 78 = UV-B availability at state of residence, 79 = height, 80 = solar UV exposure quartile calculated from summer erythemal UV values weighted by time outdoors, 81 = ever had moles removed, 82 = chronic pain in last year, 83 = kidney disease or ulcer

*GEM* the genes, environment, and melanoma study, *GPRD* general practitioners research database, *KPNC* Kaiser Permanente Northern California population, *NHS* nurses’ health study, *VITAL* the vitamins and lifestyle, *WHI* women’s health initiative

^a^Study deemed to be prone to immortal time bias

**Table 19:** Characteristics of included studies- lymphoma

Study source	Sex	Study period	Source of subjects	No of case	No of control/cohort size	Cancer site	Exposure assessment	Exposure Definition	Adjustment for covariates	Study quality
Case-control studies										
Baecklund E [245], 2006, Swedish	M/F	196–1995	From the Swedish Inpatient Register	269	225	Lymphoma	Hospital records	Use aspirin for 4 consecutive weeks	15,16	5
Zhang YQ [246], 2006, US	M/F	197–2002	Subjects were recruited from patients admitted to hospitals in New York, Philadelphia, Boston and Baltimore	412	1524	Non-Hodgkin lymphoma	Nurse-interviewers administered standard questionnaires	Use at least four times per week for at least three or more continuous months(regular)	1,2,7,8	7
Flick ED [247], 2006, US+	M/F	200–2004	Population from the California counties of San Francisco, Alameda, Marin, Contra Costa, San Mateo, and Santa Clara	604	638	Non-Hodgkin lymphoma	Interview	Use at least 2 days per week for 3 months or longer during the past 20 years	1,2,17	7
Baker JA [248], 2005, US	M/F	198–1998	RPCI	628	2512	Non-Hodgkin lymphoma	Questionnaire	Use at least once per week for 6 months	1	5
Chang ET [249], 2004, US	M/F	1997–2000	population from the greater Boston, Massachusetts, metropolitan area and in the state of Connecticut	565	679	Hodgkin’s lymphoma	Telephone interview	Use two or more tablets per Week(regular)	1,2,3,9,17	6
Zhang YW [250], 2004, US	M/F	1996–2000	Patients in Yale Cancer Center’s Rapid Case Ascertainment Shared Resource(RCA)	601	717	Non-Hodgkin lymphoma	Iinterview	Use at least once a day for a period of 6 months or longer previous to 1 year ago	1,4,10,18	7
Cohort studies										
Hollestein LM [91], 2014, Netherlands ^a^	M/F	1998–2010	PHARMO and the Eindhoven Cancer Registry	256	109,276	Lymphoma	Prescription database	Low dose aspirin (≤100 mg daily)- not further defined	1,2,11,12	8
Birmann BM [251], 2014, US	F	1976–2008	NHS	196	85,942	Multiple myeloma	Questionnaire	81-mg “baby” and 325-mg “adult” strength	1,10	8
	M	1986–2008	HPFS	132	47,029	Multiple myeloma	Questionnaire	81-mg “baby” and 325-mg “adult” strength	1,10	8
Teras LR [252], 2013, US	M/F	1992–2007	The CPS-II Nutrition Cohort Cancer Prevention Study-II (CPS-II) Nutrition Cohort	1709	149,570	Lymphoma	Questionnaire	Use aspirin ≥30 aspirin pills/Month(regular)	1,3,4,5,6,10,19,20, 21,22,23,24,25	7
Chang ET [253], 2011, Denmark^a^	M/F	1995–2008	Population from Denmark	1659	8089	Hodgkin lymphoma	Prescription database	Use aspirin ≥ 2 times per week	1,2,13,14	8
Walter RB [254], 2011, US	M/F	2000–2002	VITAL Study	224	64,839	Lymphoma	Questionnaire	Had ever taken low dose aspirin(81 mg)	4,21,23,26,27,28,29,33	6
Erber E [255], 2009, US	M/F	199–1996	MEC Study	896	193,050	Non-Hodgkin Lymphoma	Self-completed questionnaire	Use at least two times per week for 1 month or longer	5,6,10	8
Cerhan JR [256], 2003, US	M/F	199–1999	IWHS	130	27,290	Non-Hodgkin Lymphoma	Self-completed questionnaire	Had ever taken aspirin- not further defined	1,3,6,17,21,25, 29,30,31,32	7
Friis S [62], 2003, Denmark^a^	M/F	1989–1997	Population from North Jutland County	57	29,470	Non-Hodgkin’s lymphoma	Prescription database	75–150 mg once daily(low-dose aspirin)	1,2	8
Schreinemachers DM [63], 1994, US	M/F	1971–1987	The National Health and Examination Survey Ι	48	12,668	Lymphoma	Self reported	Use aspirin during the 30-day period before the interview	1,2	6

1=age, 2=sex, 3=smoking, 4=family history, 5=educational level, 6=alcohol intake, 7=year of interview, 8=study center, 9=use of other analgesics, 10=BMI, 11=unique number of dispensing, 12=unique number of hospitalizations in the year prior to start of follow up, 13=Charlson comorbidity index, 14= history of connective tissue disorder, 15=auranofin, chlorambucil, cyclophosphamide, cyclosporine, D-penicillamine, and podophyllotoxin, 16=disease activity, 17=residence, 18=menopausal status, 19=race, 20=sitting time, 21=diabetes status, 22=rheumatoid arthritis status, 23=cholesterol-lowering drug use, 24=acetaminophen use, 25=postmenopausal hormone use, 26=self-reported health, 27=history of coronary artery disease, 28=stroke, 29=marital status, 30=transfusion history,31= red meat and fruit intake,32= replacement therapy, 33=history of fatigue/lack of energy

*HPFS* Health Professionals follow-up study, *IWHS* Iowa Women’s Health Study, *MEC* multiethnic cohort study, *NHS* nurses’ health study, *RPCI* the Roswell Park Cancer Institute, *VITAL* the vitamins and lifestyle

^a^Study deemed to be prone to immortal time bias

**Table 20:** Characteristics of included studies- leukemia

Study source	Sex	Study period	Source of subjects	No of case	No of control/cohort size	Cancer site	Exposure assessment	Exposure Definition	Adjustment for covariates	Study quality
Case-control studies										
Ross JA [257], 2011, US	M/F	2005–2009	The MCSS	734	697	Leukemia	Questionnaire	Use at least once per week for at least 1 year	1,7,10	9
Weiss JR [258], 2006, US	M/F	1981–1998	RPCI	169	676	Leukemia	Questionnaire	Use at least once per week for 6 months(regular)	1,2	6
Oleske D [7], 1985, US	M/F	1975–1981	Hairy Cell Tumor Registry and Treatment Center	45	134	Leukemia	Questionnaire	Use three times a week or more for more than 2 months	1,2,6,11	6
Cohort studies										
Jacobs EJ [98], 2007, US	M/F	1992–2003	Cancer Prevention Study II Nutrition Cohort	465	146,113	Leukemia	Questionnaire	Use at least 30 “times” per month(daily use of adult-strength)	1,2,3,5,10,11,12,13,14,15,16,17,18,19	8
Kasum CM [259], 2003, US	F	1992–2000	IWHS	81	28,224	Leukemia	Questionnaire	Had ever taken aspirin- not further defined	1,3,5	8
Friis S [62], 2003, Denmark^a^	M/F	1989–1997	Population from North Jutland County	69	29,470	Leukemia	Prescription database	75–150 mg once daily(low-dose aspirin)	1,2	8
Schreinemachers DM [63], 1994, US	M/F	1971–1987	The National Health and Examination Survey Ι	39	12,668	Leukemia	Self reported	Use aspirin during the 30-day period before the interview	1,2	6

1=age, 2=sex, 3=smoking, 4=family history, 5=educational level, 6=residence, 7=other analgesic use, 8=fat distribution, 9=social status, 10=BMI, 11=race, 12=physical activity level, 13=use of hormone replacement therapy, 14=history of mammography, 15=history of colorectal endoscopy, 16=use of non-aspirin NSAIDs, 17= history of heart attack, 18=diabetes, 19=hypertension

*IWHS* Iowa Women’s Health Study, *MCSS* the Minnesota Cancer Surveillance System, *RPCI* the Roswell Park Cancer Institute

^a^Study deemed to be prone to immortal time bias

**Table 21:** Characteristics of included studies- small intestine neuroendocrine tumors

Study source	Sex	Study period	Source of subjects	No of case	No of control/cohort size	Cancer site	Exposure assessment	Exposure Definition	Adjustment for covariates	Study quality
Case-control studies										
Rinzivillo M [260], 2016, Italy	M/F	2009–2012	Population from Universities of Rome and Bologna and at the European Institute of Oncology	215	860	Small Intestine Neuroendocrine Tumors	Questionnaire	Use at any dose at least twice a week for more than one consecutive year	1,2,	7

1= age, 2=sex

### Aspirin use and the risk of cancers

**Fig. 2:**
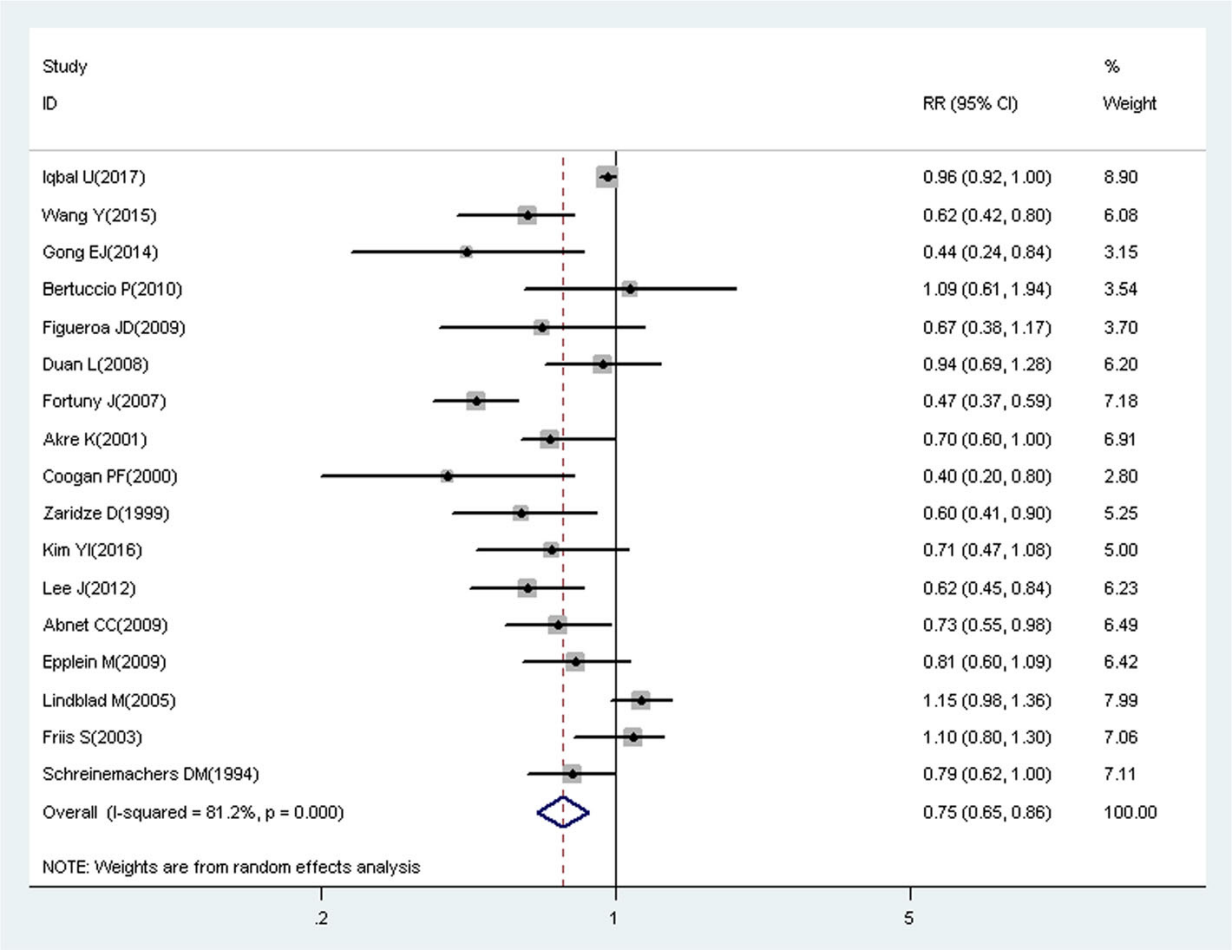
Forest plot of aspirin use and the risk of gastric cancer

**Fig. 3:**
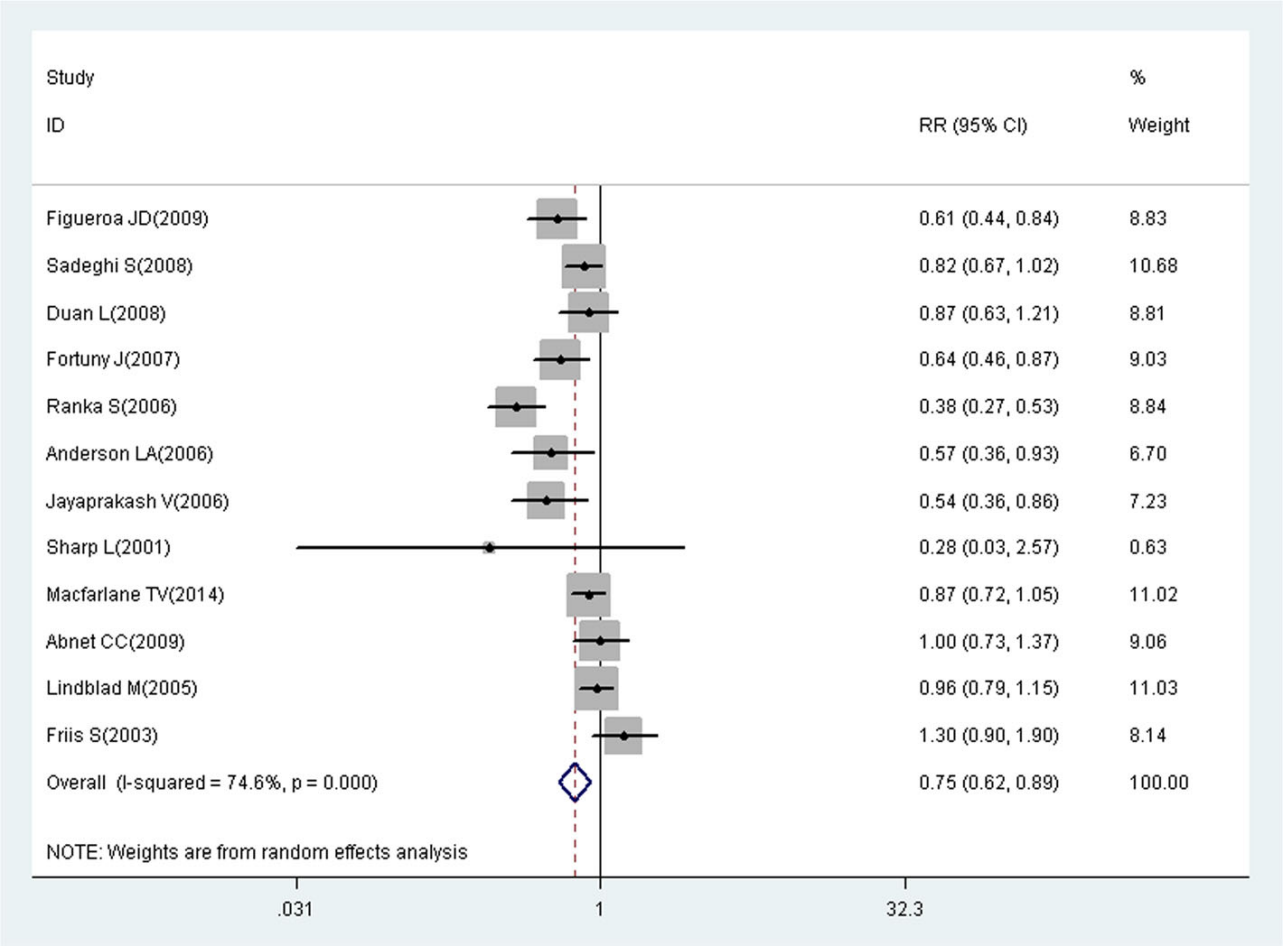
Forest plot of aspirin use and the risk of esophagus cancer

**Fig. 4:**
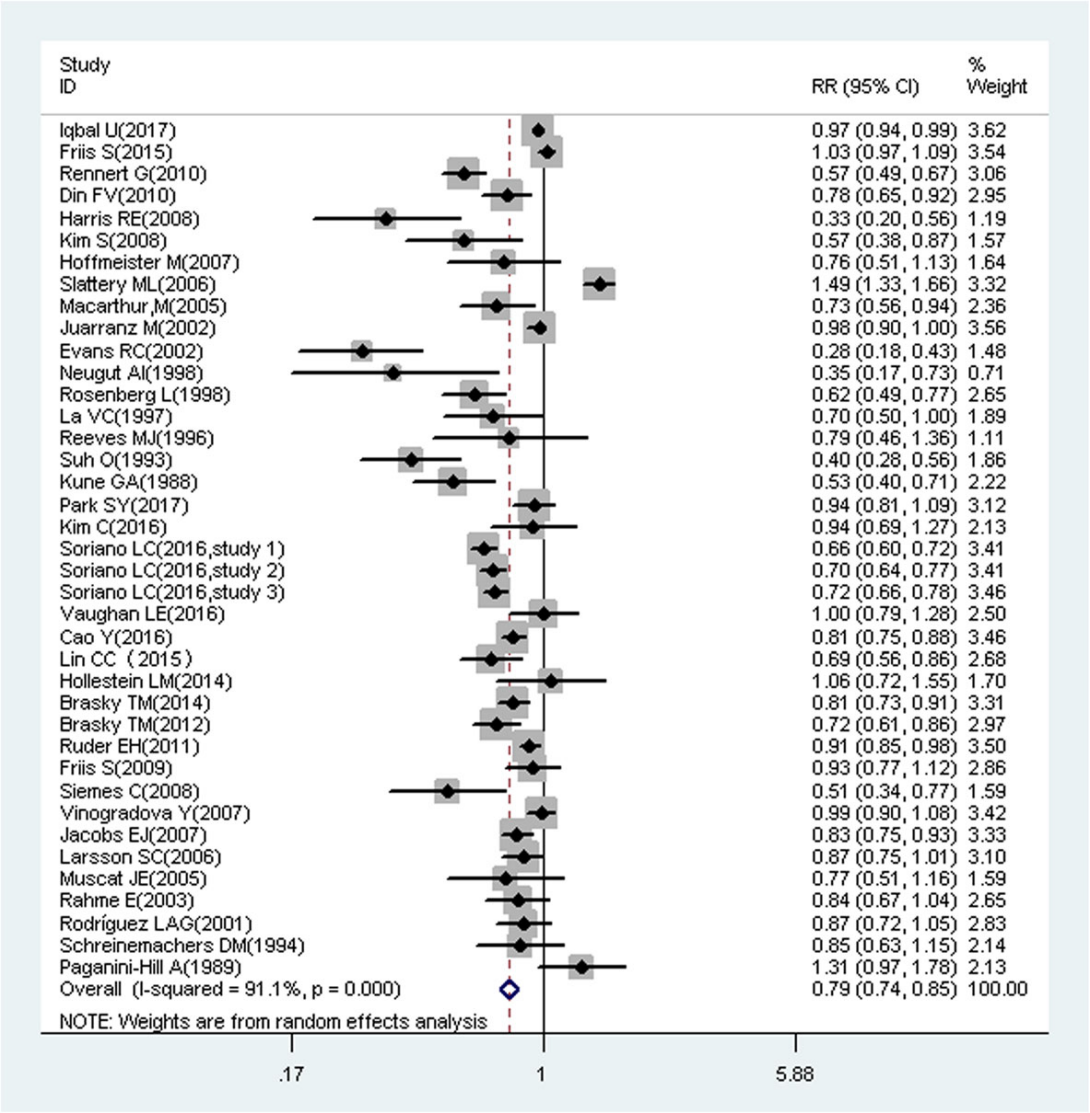
Forest plot of aspirin use and the risk of colorectal cancer

**Fig. 5:**
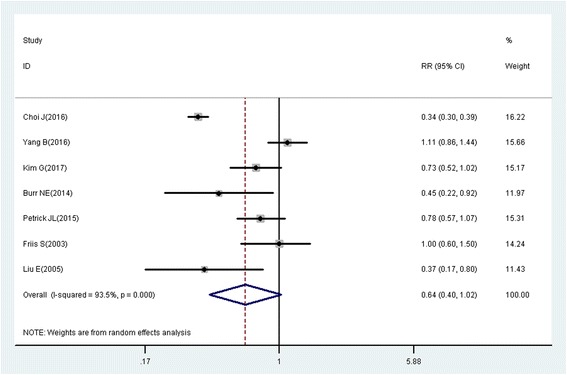
Forest plot of aspirin use and the risk of hepato-biliary cancer

**Fig. 6:**
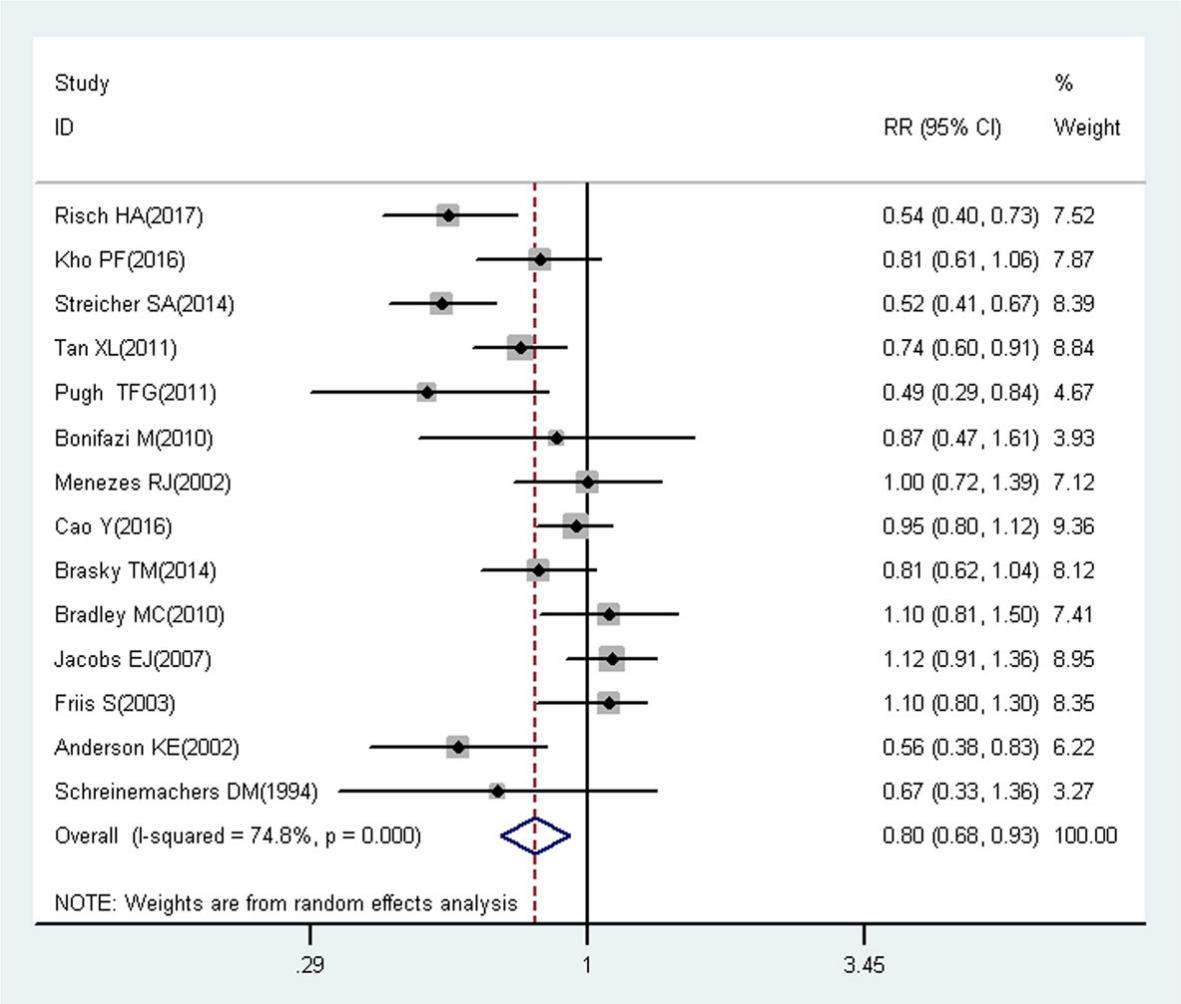
Forest plot of aspirin use and the risk of pancreatic cancer

**Fig. 7:**
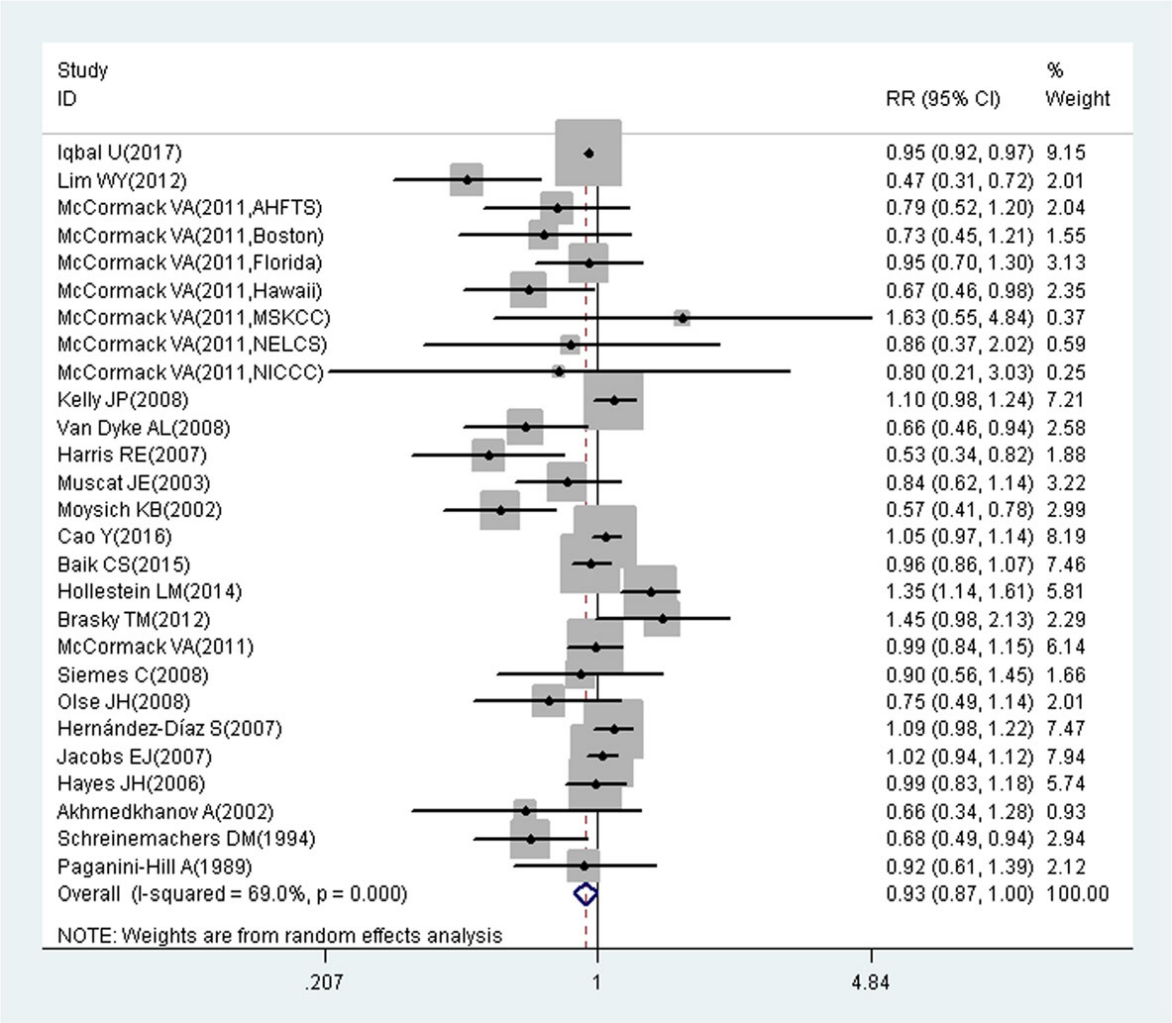
Forest plot of aspirin use and the risk of lung cancer

**Fig. 8:**
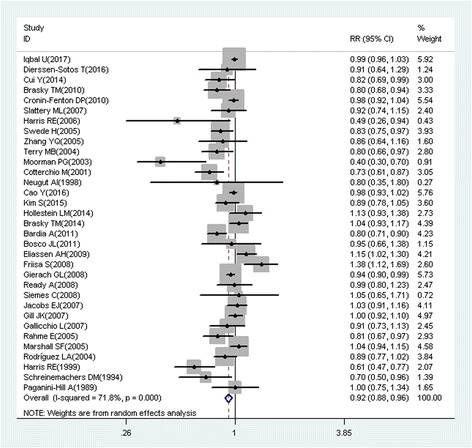
Forest plot of aspirin use and the risk of breast cancer

**Fig. 9:**
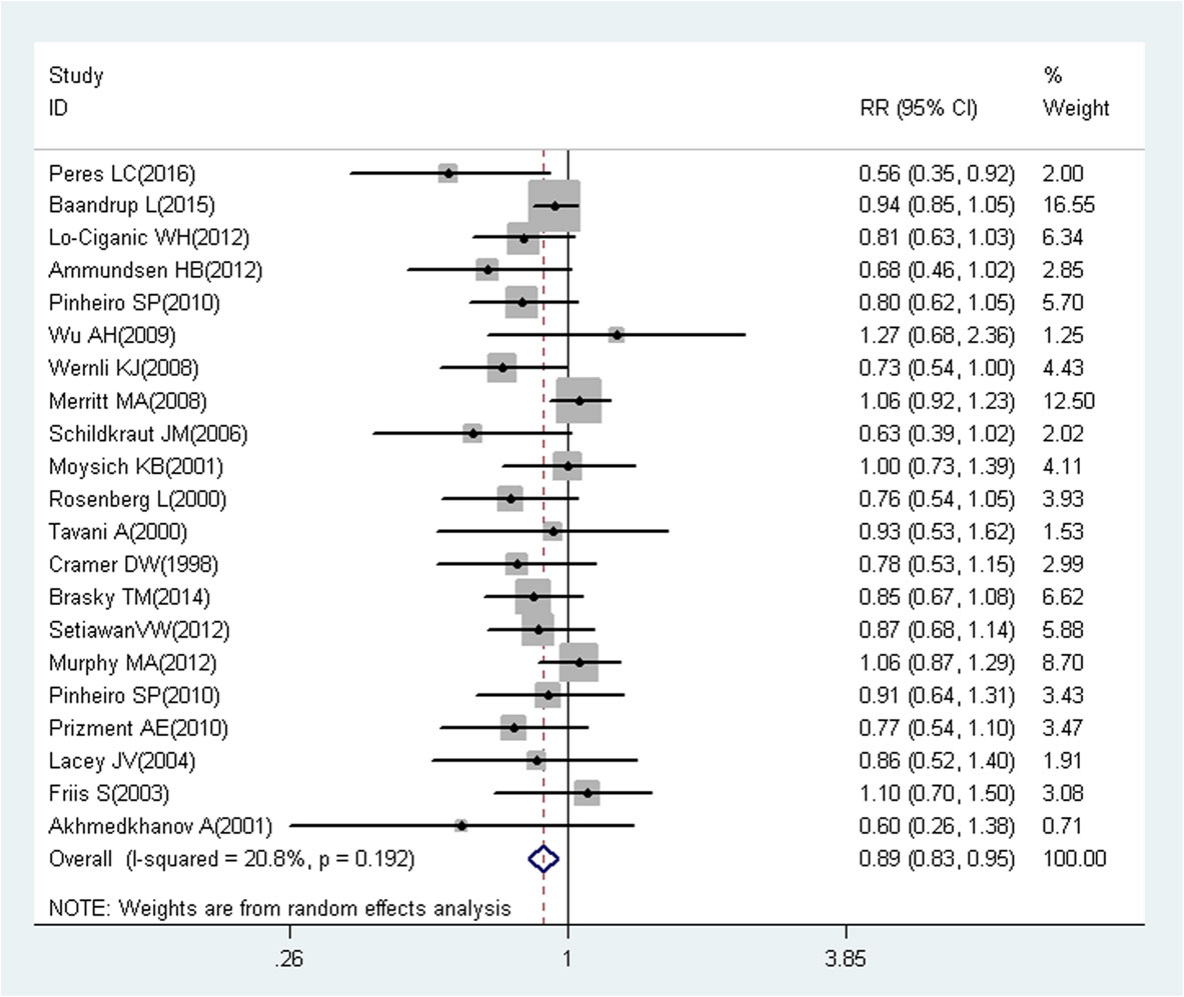
Forest plot of aspirin use and the risk of ovarian cancer

**Fig. 10:**
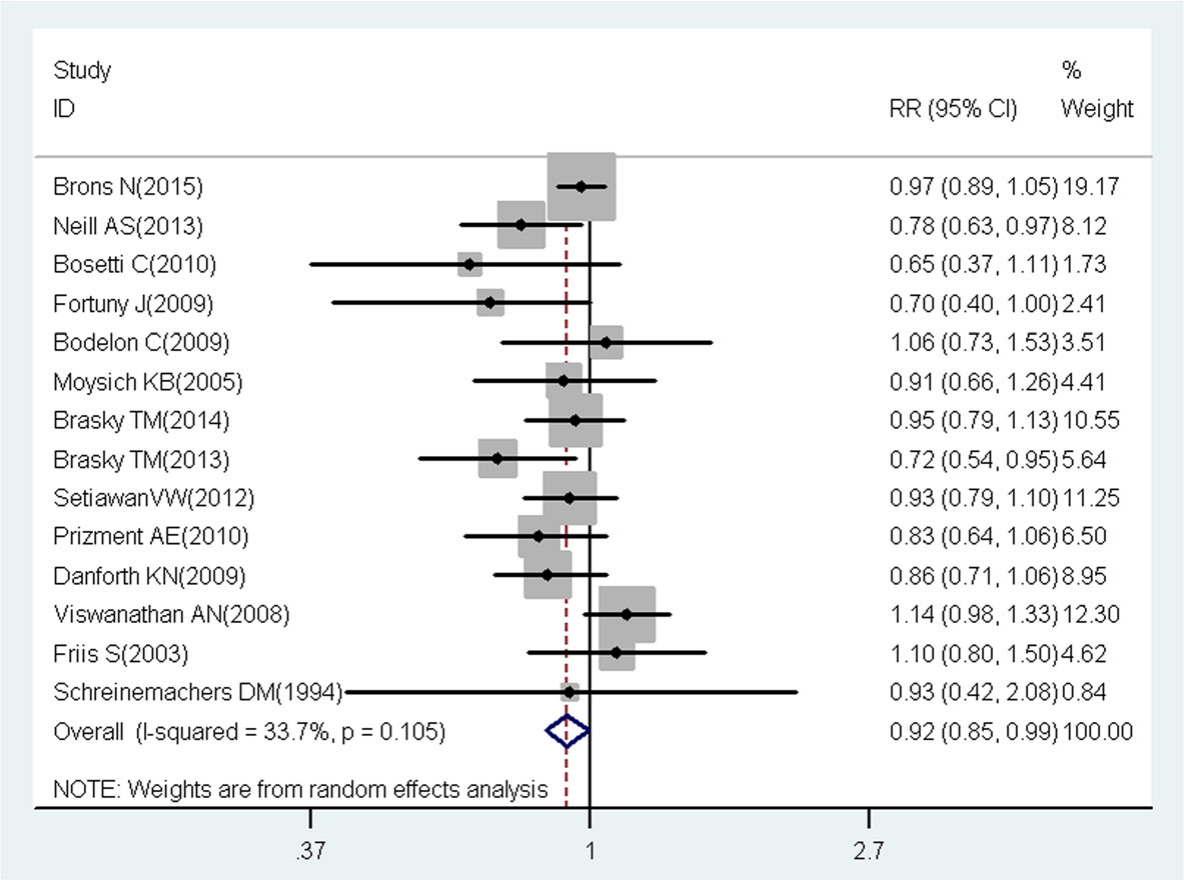
Forest plot of aspirin use and the risk of endometrial cancer

**Fig. 11:**
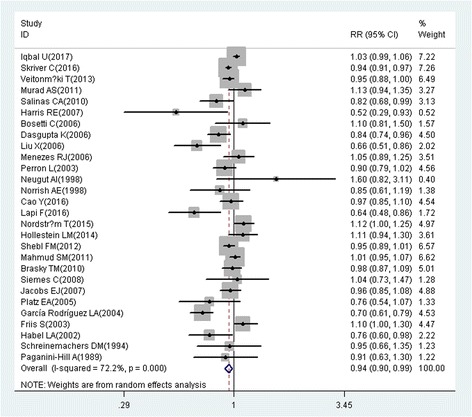
Forest plot of aspirin use and the risk of prostate cancer

**Fig. 12:**
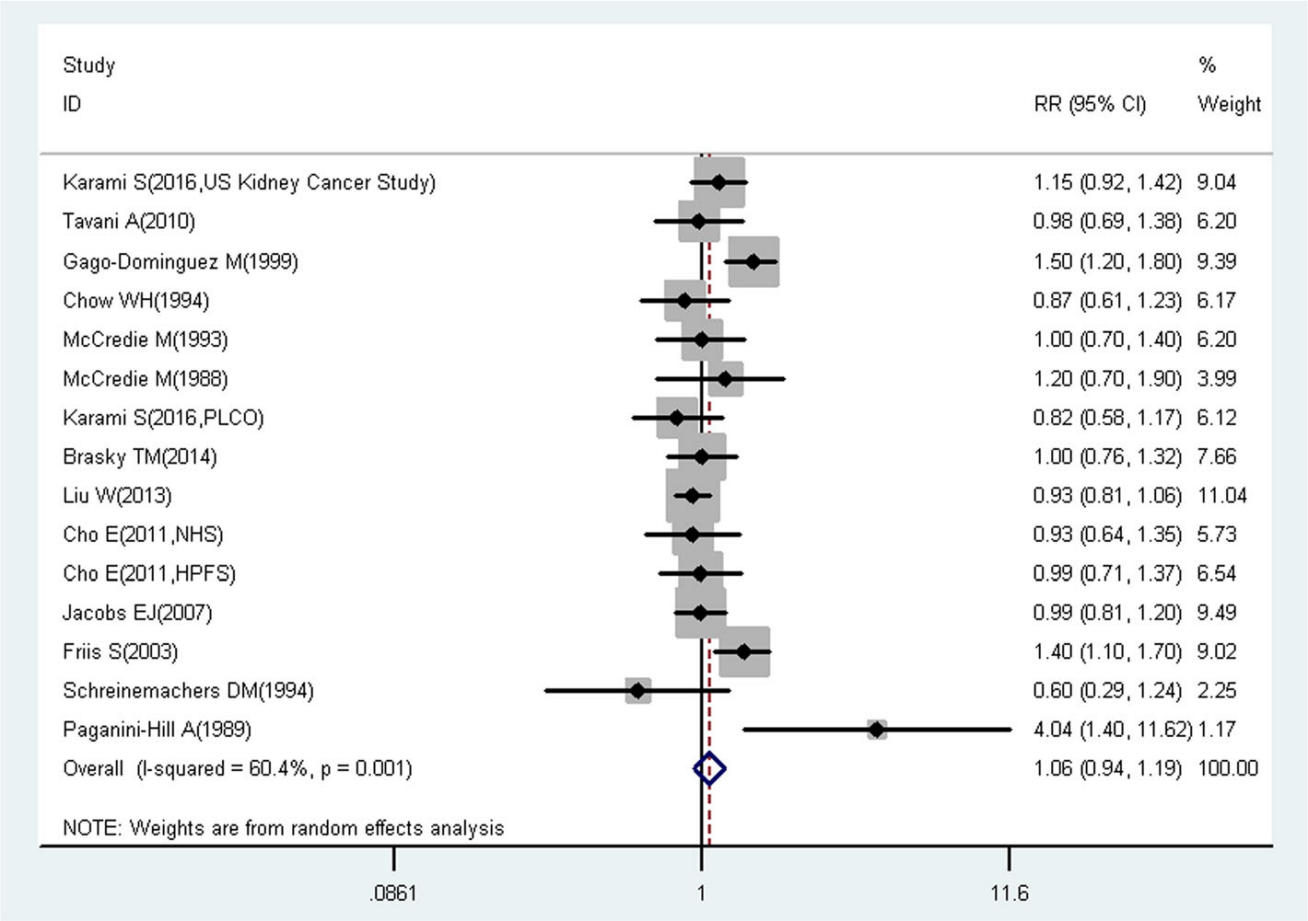
Forest plot of aspirin use and the risk of renal cancer

**Fig. 13:**
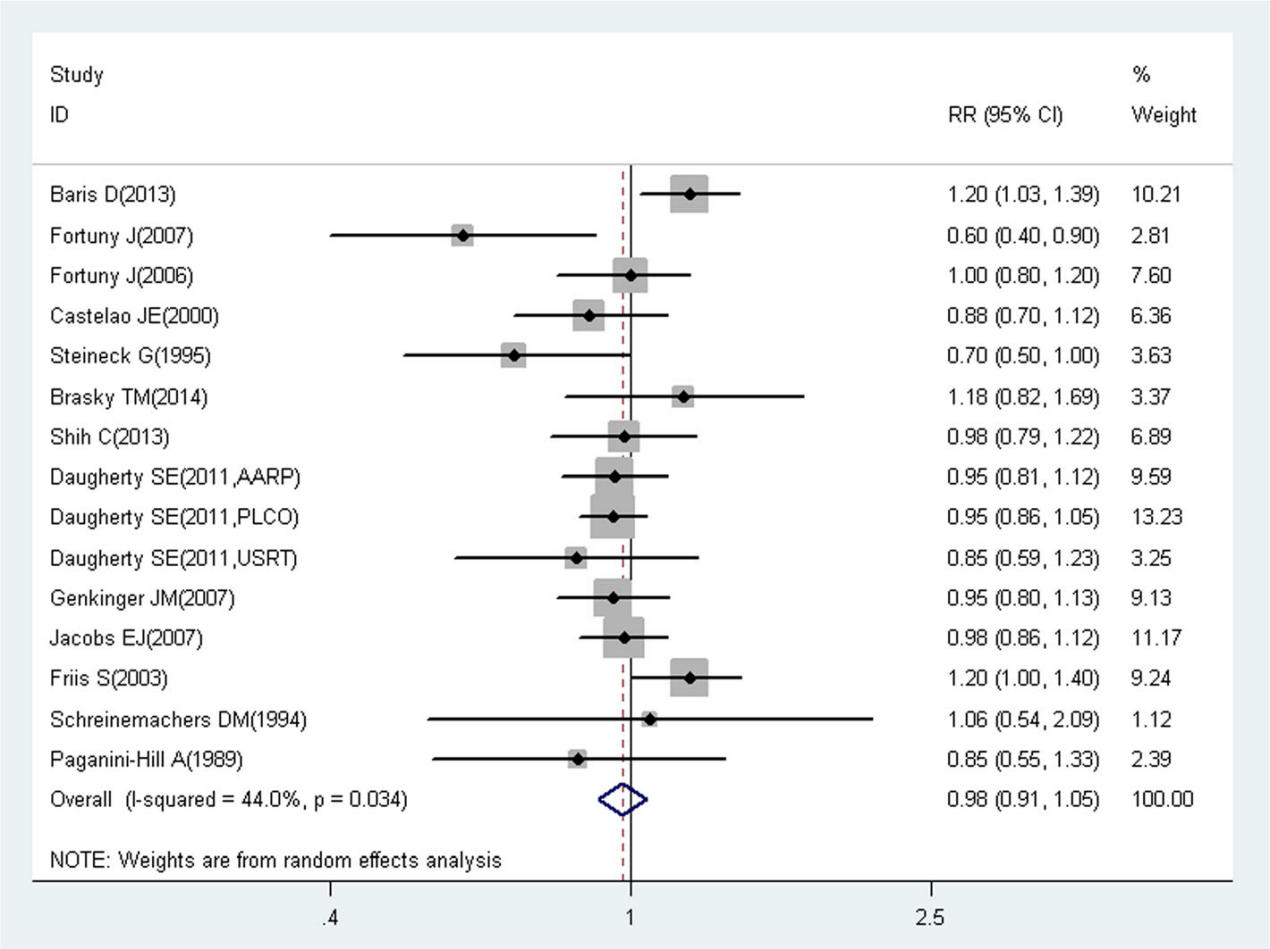
Forest plot of aspirin use and the risk of bladder cancer

**Fig. 14:**
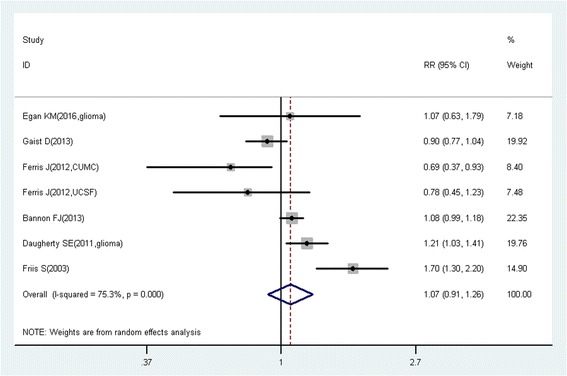
Forest plot of aspirin use and the risk of brain tumors

**Fig. 15:**
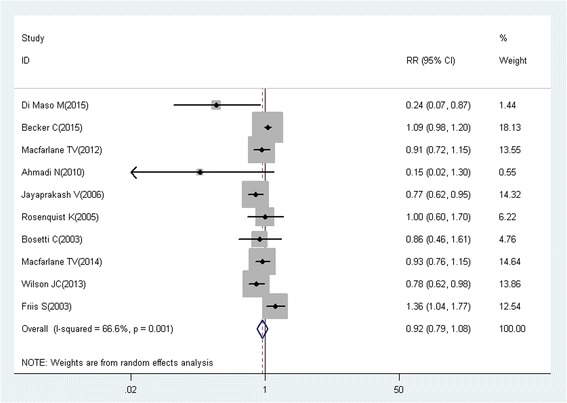
Forest plot of aspirin use and the risk of head and neck cancers

**Fig. 16:**
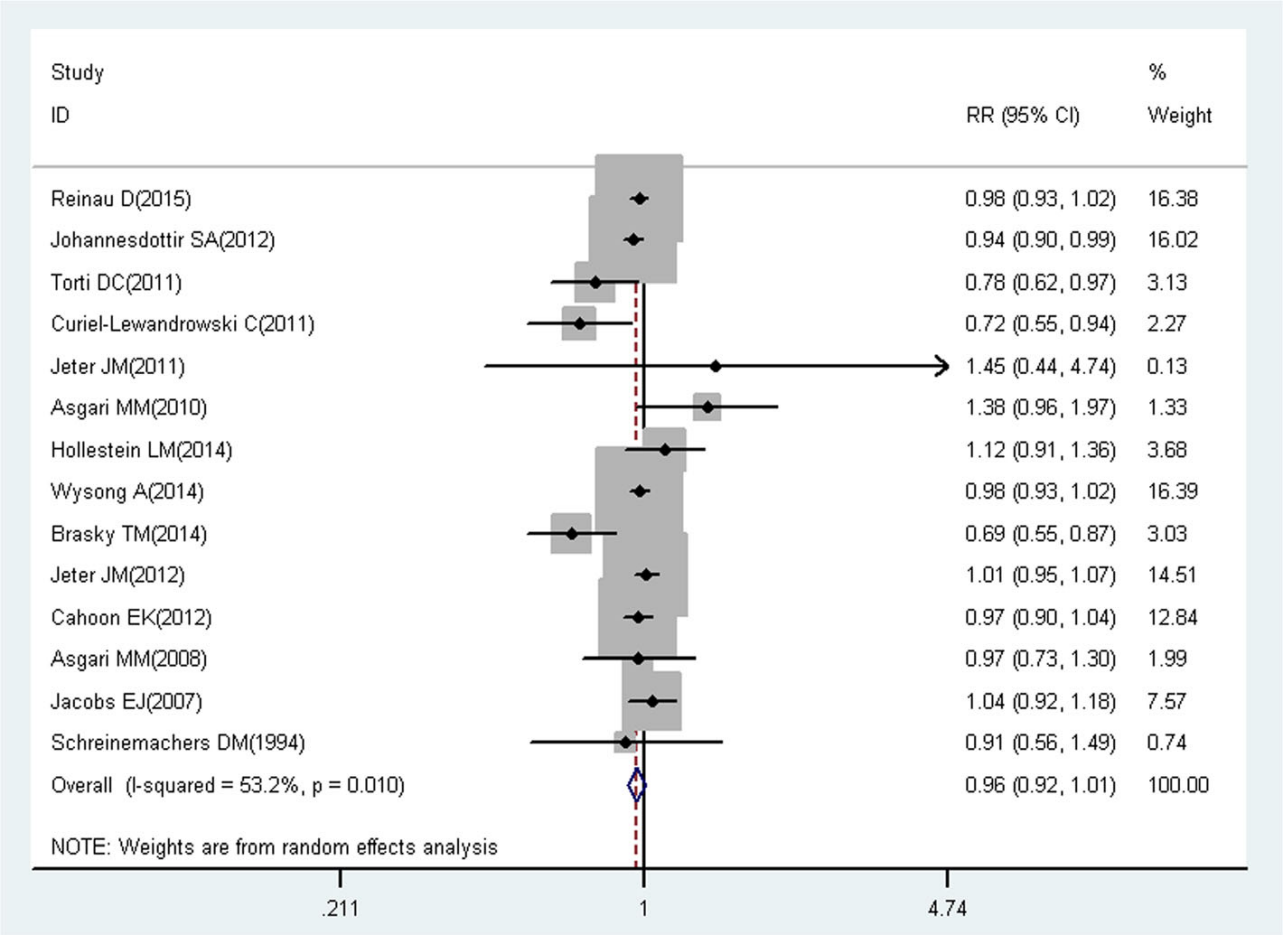
Forest plot of aspirin use and the risk of skin cancer

**Fig. 17:**
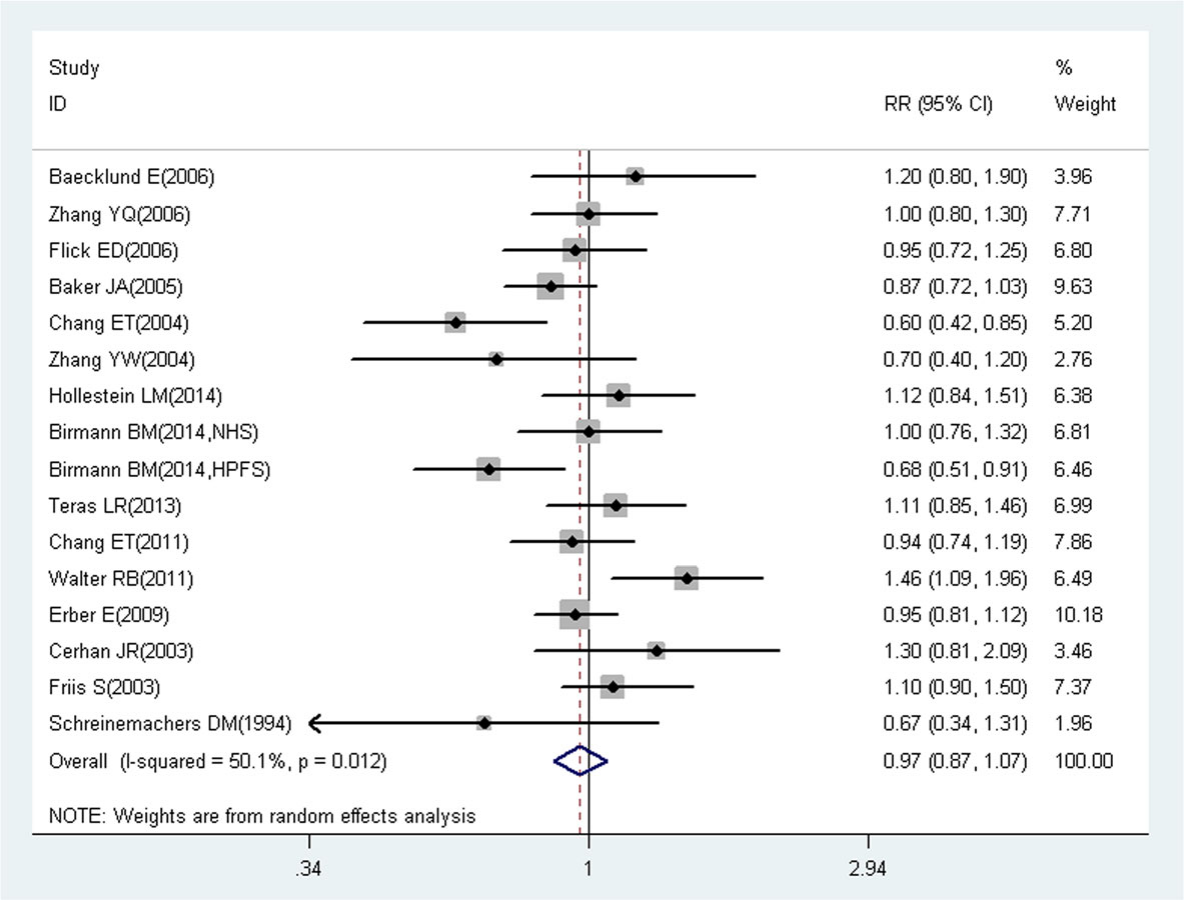
Forest plot of aspirin use and the risk of lymphoma

**Fig. 18:**
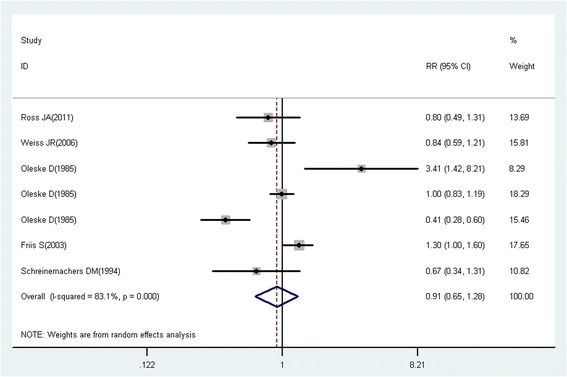
Forest plot of aspirin use and the risk of leukemia

Figures 2, 3, 4, 5, 6, 7, 8, 9, 10, 11, 12, 13, 14, 15, 16, 17 and 18 and Additional file 1: Table S1 shows the RRs for the 21 separate cancer sites that we assessed and that of the total cancers. The use of aspirin was associated with a reduced cancer risk for ten specific sites: gastric cancer (RR =0.75, 95%CI:0.65–0.86), esophagus cancer (RR = 0.75, 95%CI:0.62–0.89), colorectal cancer(RR = 0.79, 95%CI:0.74–0.85), pancreatic cancer (RR = 0.80, 95%CI:0.68–0.93), breast cancer (RR = 0.92, 95%CI:0.88–0.96), ovarian cancer (RR = 0.89, 95%CI:0.83–0.95), endometrial cancer (RR = 0.92, 95%CI:0.85–0.99), prostate cancer (RR = 0.94, 95%CI:0.90–0.99), and small intestine neuroendocrine tumors (RR = 0.17, 95%CI:0.05–0.58). However, there was no significant association between aspirin use and the risk of some cancers, including hepato-biliary, lung, cervical uterus, renal, renal pelvis and ureter, bladder, brain, head and neck, thyroid, and skin cancers, as well as lymphoma and leukemia.

Additional file 1: Tables S1–S18 shows the RRs for cancers at 17 sites, in subgroups of studies defined by their design, study location, gender, exposure assessment, quality assessment, duration of aspirin use, and frequency of aspirin use.

We conducted a subgroup analysis stratified by questionnaires and medical records, and found a lower risk in medical records with most cancers (gastric, esophageal, colorectal, hepato-biliary, and pancreatic cancers), however, significant heterogeneity of effects was noted for those subgroups (Additional file 1: Tables S2–S18). As we expected, the decreased risk of colorectal cancer (RRs = 0.76, 95%CI: 0.66–0.87 for ≥5 years), pancreatic cancer (RRs = 0.75, 95%CI: 0.57–0.99 for ≥5 years), ovarian cancer (RRs = 0.77, 95%CI: 0.63–0.93 for ≥5 years), and brain cancer (RRs = 0.65, 95%CI: 0.43–0.97 for ≥5 years) were more pronounced with longer duration of aspirin use. However, the aspirin-associated RR for 21 specific cancers did not vary significantly by other characteristics (gender, quality assessment and frequency of aspirin use).

### Publication bias

**Fig. 19:**
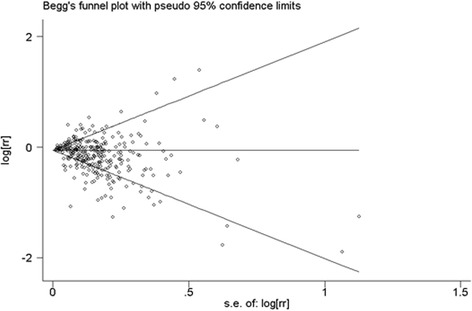
Funnel plot of aspirin use and cancer

The funnel plot showed asymmetry (Fig. 19). In addition, the Begg’s test and Egger’s test provided evidence of publication bias among the included studies (Begg’s test *Z* = 4.34, *P* < 0.001; Egger’s test *Z* = − 5.27, *P* < 0.001).

## Discussion

The results of our meta-analysis supported the presence of inverse associations between aspirin use and the risk of overall cancer, gastric, esophageal, colorectal, pancreatic, breast, ovarian, endometrial, and prostate cancers, as well as small intestine neuroendocrine tumors. However, no significant associations were observed between the use of aspirin and the risk of other cancers, including hepato-biliary, lung, cervical uterus, renal, renal pelvis and ureter, bladder, brain, head and neck, thyroid, and skin cancers, as well as lymphoma, and leukemia.

There are several potential biological mechanisms through which aspirin could reduce the risk of cancer. First, aspirin and other NSAIDs have been proven to inhibit the activity of the enzyme cyclooxygenase 2 (COX-2), which is responsible for the synthesis of prostaglandins [29]. COX-2 has been reported to be overexpressed in many cancers and participates in key cellular activities, including cell proliferation, apoptosis, angiogenesis, and metastasis [30–32]. Second, aspirin could activate the NF-kappa B (NF-κB) signaling pathway, which triggers apoptosis in neoplasia [33, 34]. In addition, some studies showed that aspirin might induce gene selection and modulate mitochondrial voltage dependent anion channels (VDACs) to reduce the risk of cancer progression and metastasis [35, 36].

The results of this meta-analysis indicated that utilization of aspirin had different protective effects on the development of cancer. This difference may be attributed to the different expression levels of COX in various cancers [37]. Furthermore, Zumwalt et al. [38] reported that the effectiveness of aspirin was primarily determined by specific genetic variants. Aspirin inhibited cell growth in all cancer cell lines regardless of mutational background, however, the effects were exacerbated in cells with PIK3CA mutations, which might explain the different effects of aspirin on cancers.

The decreased risk of gastric, esophageal, pancreatic, lung, breast, and ovarian cancers was observed in the case-control studies but not in the cohort studies. One possible explanation for the difference might be that cases in the case-control studies might have a recall bias and tended to overestimate the risk of cancer by aspirin use. Another possible explanation is that misclassification or measurement errors for aspirin use in the cohort studies might have distorted the association because most of our analyses were based on baseline data, and there might be a discrepancy between initial recruitment and subsequent aspirin consumption.

The longer those who had used aspirin, the lower their risk of cancer was, with longer duration of use associated with an RR of 0.90 (95% CI 0.89–0.74), based on 118 studies that reported associations with longer (≥5 years) duration of aspirin use and 105 studies that reported associations with shorter (< 5 years) duration of aspirin use. For most cancers (colorectal, pancreatic, ovarian, and brain cancers), risk reductions were more pronounced with longer duration of use, and these results agree with those of previous studies [39–41]. In addition, the United States Preventive Services Task Force (USPSTF) indicated that cancer prevention was a significant aspect in the overall health benefit of aspirin, but this benefit was not apparent until several years after the initiation of aspirin therapy [42, 43]. It is of note that a significant inverse association with prostate cancer was observed in the patients who took aspirin for less than 5 years. Indeed, after the study that relied on the General Practice Research Database [44] was excluded, the discrepancy disappeared. Considering that aspirin use was off-prescription in the United Kingdom, misclassification was likely to occur in this study because many commonly used aspirins do not require a prescription. Therefore, it can be deduced that the patients who used aspirin for at least 5 years were more likely to realize the potential cancer prevention benefit.

There was no statistically significant difference between the pooled RRs for the frequency of aspirin in most studies. Given that a few studies were included in the subgroup analysis on the basis of the frequency of aspirin use and most studies lacked information on this variable, the results on the risks associated with the frequency of aspirin use should be interpreted with caution. Further studies that explore the associations between the frequency of aspirin use and cancer risk are necessary to elucidate the effects of aspirin.

In addition, our results indicated that the strongest reduction in the risk of most cancers associated with aspirin was found in North American countries. However, two-thirds of the included studies were performed in North America and a few studies were performed in Asian and European countries, which might distort the accuracy of the results. Therefore, more studies are necessary to examine the discrepancies among the different countries and regions.

### Comparison with other studies

Bosetti et al. (2011) [45] conducted a meta-analysis on aspirin and 12 selected cancer sites based on 139 observational studies and 187,167 cases. Our study included 218 studies involving 737,409 cases and examined the correlation between aspirin use and the risk of skin, head and neck, hepatobiliary, thyroid, cervical uterus, renal pelvis, ureter, and brain cancers, lymphoma, small intestine neuroendocrine tumors, and leukemia, thereby providing more comprehensive and reliable evidence for this correlation. More importantly, this study was the first meta-analysis to evaluate the association between aspirin use and the risk of hepatobiliary cancer and we found a non-significant effect of aspirin on the risk of hepatobiliary cancer (OR = 0.64, 95% CI: 0.40–1.02).

Algra and Rothwell (2012) [46] conducted a meta-analysis on the association between aspirin use and the risk of cancer based on 195 studies and 215,211 cases. Compared with their review, our meta-analysis have added approximately 70 new articles published since 2012, with a total of 737,409 cases, which significantly enhanced the statistical power to determine this potential association. In addition, the exposure in the previous review was inconsistent, which may mislead the estimation. Many studies defined aspirin as the exposure but only a few studies defined NSAIDs as the exposure, and thus the specific effect of aspirin on cancers was not defined. The exposure to aspirin in our meta-analysis was consistent and ensured the reliability of the findings.

### Strengths and limitations

This study is the most up-to-date comprehensive review of the effect of aspirin use on the risk of all types of cancers, and the large sample size provides reliable results with greater precision and power. The potential limitations of this study should be noted. First, there was substantial heterogeneity across the included studies, which was likely due to differences in the definitions of exposure, units, assessment methods, and the adjusted variables across different studies. Second, misclassification or measurement errors for aspirin use might distort the association because our analyses were based on baseline data, and changes in the exposure to aspirin were not updated during the follow-up period. Third, the visual inspection of a funnel plot showed asymmetry, and the Begg’s test and Egger’s test also identified evidence of publication bias among the studies included in our meta-analysis.

Our meta-analysis indicated a beneficial role for aspirin for overall cancers; however, the results should be interpreted with caution. Considering that most evaluated studies were based on secondary prevention rather than on primary prevention, the totality of evidence for the high-risk population was incomplete, and it is appropriate to let the beneficial role remain uncertain. At present, we should accept the uncertainties, and future chemoprevention trials should clarify the extent to which aspirin decreases cancers incidence.

## Conclusions and implications

Evidence from observational studies indicates that utilization of aspirin is associated with reduced risk of gastric, colorectal, esophageal, pancreatic, ovarian, endometrial, breast, and prostate cancers, in addition to small intestine neuroendocrine tumors. A stronger protective effect was observed in the North American populations and patients who used aspirin for at least 5 years. It is important to address immortal time bias not only to ensure the integrity of the meta-analysis, but also to ensure the integrity of pharmacoepidemiological studies. Moreover, given the confidence limits of the evaluated studies, adequately powered mechanistic studies should help elucidate the mechanisms underlying this correlation.

## Additional file

Additional file 1: **Table S1.** Summary table. **Table S2.** Subgroup analysis of relative risk of gastric cancer. **Table S3.** Subgroup analysis of relative risk of esophagus cancer. **Table S4.** Subgroup analysis of relative risk of colorectal cancer. **Table S5.** Subgroup analysis of relative risk of hepato-biliary cancer. **Table S6.** Subgroup analysis of relative risk of pancreatic cancer. **Table S7.** Subgroup analysis of relative risk of lung cancer. **Table S8.** Subgroup analysis of relative risk of breast cancer. **Table S9.** Subgroup analysis of relative risk of ovarian cancer. **Table S10.** Subgroup analysis of relative risk of endometrial cancer. **Table S11.** Subgroup analysis of relative risk of prostate cancer. **Table S12.** Subgroup analysis of relative risk of renal cancer. **Table S13.** Subgroup analysis of relative risk of bladder cancer. **Table S14.** Subgroup analysis of relative risk of brain tumor. **Table S15.** Subgroup analysis of relative risk of head and neck cancers. **Table S16.** Subgroup analysis of relative risk of skin cancer. **Table S17.** Subgroup analysis of relative risk of lymphoma. **Table S18.** Subgroup analysis of relative risk of leukemia. (DOC 549 kb)

## Abbreviations

CI: Confidence interval
COX-2: Cyclooxygenase 2
HRs: Hazard ratios
NF-κB: NF-kappa B
NSAIDs: Non-steroidal anti-inflammatory drugs
ORs: Odds ratios
RRs: Relative risks
USPSTF: United States Preventive Services Task Force
VDACs: Voltage dependent anion channels
