# Supplementary material for: Associations between aspirin use and the risk of cancers: a meta-analysis of observational studies
Source: BMC Cancer. 2018 Mar 13;18:288. doi: 10.1186/s12885-018-4156-5 (PMC5851082; doi:10.1186/s12885-018-4156-5)
Supplement: Supplementary file 1 — Table S1. Summary table. Table S2. Subgroup analysis of relative risk of gastric cancer. Table S3. Subgroup analysis of relative risk of esophagus cancer. Table S4. Subgroup analysis of relative risk of colorectal cancer. Table S5. Subgroup analysis of relative risk of hepato-biliary cancer. Table S6. Subgroup analysis of relative risk of pancreatic cancer. Table S7. Subgroup analysis of relative risk of lung cancer. Table S8. Subgroup analysis of relative risk of breast cancer. Table S9. Subgroup analysis of relative risk of ovarian cancer. Table S10. Subgroup analysis of relative risk of endometrial cancer. Table S11. Subgroup analysis of relative risk of prostate cancer. Table S12. Subgroup analysis of relative risk of renal cancer. Table S13. Subgroup analysis of relative risk of bladder cancer. Table S14. Subgroup analysis of relative risk of brain tumor. Table S15. Subgroup analysis of relative risk of head and neck cancers. Table S16. Subgroup analysis of relative risk of skin cancer. Table S17. Subgroup analysis of relative risk of lymphoma. Table S18. Subgroup analysis of relative risk of leukemia. (DOC 549 kb) [file 12885_2018_4156_MOESM1_ESM.doc]

**Additional file**

Table S1 Summary table

Table S2 Subgroup analysis of relative risk of gastric cancer

Table S3 Subgroup analysis of relative risk of esophagus cancer

Table S4 Subgroup analysis of relative risk of colorectal cancer

Table S5 Subgroup analysis of relative risk of hepato-biliary cancer

Table S6 Subgroup analysis of relative risk of pancreatic cancer

Table S7 Subgroup analysis of relative risk of lung cancer

Table S8 Subgroup analysis of relative risk of breast cancer

Table S9 Subgroup analysis of relative risk of ovarian cancer

Table S10 Subgroup analysis of relative risk of endometrial cancer

Table S11 Subgroup analysis of relative risk of prostate cancer

Table S12 Subgroup analysis of relative risk of renal cancer

Table S13 Subgroup analysis of relative risk of bladder cancer

Table S14 Subgroup analysis of relative risk of brain tumor

Table S15 Subgroup analysis of relative risk of head and neck cancers

Table S16 Subgroup analysis of relative risk of skin cancer

Table S17 Subgroup analysis of relative risk of lymphoma

Table S18 Subgroup analysis of relative risk of leukemia

**Table S1**. Summary table

| Cancer site | No. of reports | No. of cases | Relative risk (95%*CI*) | *I*^2^ | No. of reports  for ≥5 years | Relative risk(95%*CI*) for ≥5 years | *I*^2^ for ≥5 year |
| --- | --- | --- | --- | --- | --- | --- | --- |
| Gastric cancer | 17 | 28589 | 0.75(0.65-0.86) | 81.2% | 4 | 0.77(0.58-1.03) | 0.0% |
| Esophagus cancer | 12 | 5198 | 0.75(0.62-0.89) | 74.6% | 2 | 0.74(0.49-1.10) | 0.0% |
| Colorectal cancer | 39 | 151367 | 0.79(0.74-0.85) | 91.1% | 14 | 0.76(0.66-0.87) | 78.4% |
| Hepato-biliary cancer | 7 | 4812 | 0.64(0.40-1.02) | 93.5% |  | - | - |
| Pancreatic cancer | 14 | 5235 | 0.80(0.68-0.93) | 74.8% | 6 | 0.75(0.57-0.99) | 48.5% |
| Lung cancer | 27 | 90348 | 0.93(0.87-1.00) | 69.0% | 9 | 0.97(0.28-1.15) | 51.6% |
| Breast cancer | 33 | 127559 | 0.92(0.88-0.96) | 71.8% | 13 | 0.96(0.88-1.03) | 38.6% |
| Ovarian cancer | 21 | 14666 | 0.89(0.83-0.95) | 20.8% | 11 | 0.77(0.63-0.93) | 12.3% |
| Endometrial cancer | 14 | 11587 | 0.92(0.85-0.99) | 33.7% | 7 | 1.01(0.89-1.15) | 19.5% |
| Cervix uterus | 4 | 1040 | 0.89(0.69-1.14) | 0.0% |  | - | - |
| Prostate cancer | 28 | 126859 | 0.94(0.90-0.99) | 72.2% | 9 | 0.90(0.81-1.00) | 78.1% |
| Renal cancer | 15 | 6605 | 1.06(0.94-1.19) | 60.4% | 7 | 1.16(0.98-1.37) | 0.0% |
| Renal pelvis and ureter | 4 | 842 | 1.23(0.65-2.34) | 79.7% |  | - | - |
| Bladder cancer | 15 | 8516 | 0.89(0.91-1.05) | 44.0% | 11 | 1.10(0.95-1.28) | 0.0% |
| Brain tumor | 7 | 10365 | 1.07(0.91-1.26) | 75.3% | 3 | 0.65(0.43-0.97) | 68.6% |
| Head and neck cancers | 10 | 7727 | 0.92(0.79-1.08) | 66.6% |  | - | - |
| Thyroid cancer | 4 | 710 | 0.99(0.80-1.23) | 0.0% |  | - | - |
| Skin cancer | 14 | 125181 | 0.96(0.92-1.01) | 53.2% | 9 | 0.98(0.87-1.10) | 63.8% |
| Lymphoma | 16 | 8386 | 0.97(0.87-1.07) | 50.1% | 10 | 0.99(0.88-1.10) | 30.3% |
| Leukemia | 7 | 1602 | 0.91(0.65-1.28) | 83.1% | 3 | 0.84(0.69-1.02) | 0.0% |
| Small intestine neuroendocrine tumors | 1 | 215 | 0.17(0.05-0.58) | - |  | - | - |
| Total cancer | 309 | 737409 | 0.89(0.87-0.91) | 80.2% | 118 | 0.90(0.86-0.94) | 62.8 |

**Table S2.**Subgroup analysis of relative risk of gastric cancer

|  | No. of reports | Relative risk | 95%*CI* | *I*^2^ | *P* for heterogeneity |
| --- | --- | --- | --- | --- | --- |
| Study design |  |  |  |  |  |
| Case-control | 10 | 0.67 | 0.53-0.85 | 85.8% | <0.001 |
| Cohort | 7 | 0.84 | 0.70-1.02 | 72.3% | 0.001 |
| Exposure assessment |  |  |  |  |  |
| Prescription | 2 | 0.79 | 0.52-1.22 | 86.9% | 0.006 |
| Questionnaire | 8 | 0.71 | 0.59-0.85 | 41.2% | 0.104 |
| Interviews | 2 | 0.69 | 0.55-0.88 | 0.0% | 0.892 |
| Records | 1 | 0.47 | 0.37-0.59 | - | - |
| Database | 3 | 1.03 | 0.83-1.29 | 55.4% | 0.106 |
| Self report | 1 | 0.79 | 0.62-1.00 | - | - |
| Quality assessment |  |  |  |  |  |
| <7 | 4 | 0.66 | 0.54-0.81 | 81.2% | <0.001 |
| ≥7 | 13 | 0.79 | 0.67-0.92 | 82.1% | <0.001 |
| Study location |  |  |  |  |  |
| North America | 7 | 0.69 | 0.55-0.86 | 68.9% | 0.004 |
| Asia | 5 | 0.69 | 0.51-0.93 | 81.4% | <0.001 |
| Europe | 5 | 0.90 | 0.69-1.18 | 76.9% | 0.002 |
| Duration of aspirin use |  |  |  |  |  |
| <5 years | 6 | 0.98 | 0.81-1.19 | 45.5% | 0.103 |
| ≥5 years | 4 | 0.77 | 0.58-1.03 | 0.0% | 0.855 |
| Sex |  |  |  |  |  |
| Men | 3 | 0.82 | 0.56-1.21 | 53.6% | 0.116 |
| Women | 3 | 0.70 | 0.55-0.89 | 0.0% | 0.405 |
| Combined | 14 | 0.75 | 0.64-0.87 | 82.1% | <0.001 |
| Frequency of aspirin use |  |  |  |  |  |
| <7 tablets/week | 2 | 0.75 | 0.38-1.46 | 77.4% | 0.035 |
| ≥7 tablets/week | 3 | 0.69 | 0.50-0.96 | 14.1% | 0.312 |

**Table S3.**Subgroup analysis of relative risk of esophagus cancer

|  | No. of reports | Relative risk | 95%*CI* | *I*^2^ | *P* for heterogeneity |  |
| --- | --- | --- | --- | --- | --- | --- |
| Study design |  |  |  |  |  |  |
| Case-control | 8 | 0.62 | 0.50-0.77 | 64.5% | 0.006 |  |
| Cohort | 4 | 0.97 | 0.85-1.10 | 17.6% | 0.303 |  |
| Exposure assessment |  |  |  |  |  |  |
| Questionnaire | 4 | 0.82 | 0.67-1.00 | 42.0% | 0.160 |  |
| Interviews | 3 | 0.59 | 0.45-0.77 | 0.0% | 0.791 |  |
| Records | 2 | 0.49 | 0.30-0.82 | 79.5% | 0.027 |  |
| Database | 3 | 0.97 | 0.81-1.16 | 43.7% | 0.169 |  |
| Quality assessment |  |  |  |  |  |  |
| <7 | 3 | 0.67 | 0.50-0.91 | 52.0% | 0.125 |  |
| ≥7 | 9 | 0.78 | 0.62-0.97 | 78.9% | <0.001 |  |
| Study location |  |  |  |  |  |  |
| North America | 5 | 0.72 | 0.58-0.90 | 54.0% | 0.069 |  |
| Europe | 6 | 0.74 | 0.53-1.05 | 84.4% | <0.001 |  |
| Australia | 1 | 0.82 | 0.67-1.02 | - | - |  |
| Duration of aspirin use |  |  |  |  |  |  |
| <5 years | 3 | 0.81 | 0.52-1.27 | 70.1% | 0.035 |  |
| ≥5 years | 2 | 0.74 | 0.49-1.10 | 0.0% | 0.709 |  |
| Sex |  |  |  |  |  |  |
| Men | 1 | 1.30 | 0.77-2.20 | - | - |  |
| Women | 2 | 0.92 | 0.21-4.07 | 49.9% | 0.158 |  |
| Combined | 10 | 0.72 | 0.60-0.86 | 74.7% | 0.001 |  |

**Table S4.**Subgroup analysis of relative risk of colorectal cancer

|  | No. of reports | Relative risk | 95%*CI* | *I*^2^ | *P* for heterogeneity |
| --- | --- | --- | --- | --- | --- |
| Study design |  |  |  |  |  |
| Case-control | 17 | 0.71 | 0.63-0.80 | 93.4% | <0.001 |
| Cohort | 22 | 0.83 | 0.78-0.89 | 78.5% | <0.001 |
| Exposure assessment |  |  |  |  |  |
| Prescription | 6 | 0.74 | 0.61-0.89 | 96.5% | <0.001 |
| Questionnaire | 23 | 0.80 | 0.72-0.88 | 89.1% | <0.001 |
| Interviews | 2 | 0.56 | 0.49-0.65 | 0.0% | 0.663 |
| Records | 1 | 0.35 | 0.17-0.73 | - | - |
| Database | 5 | 0.93 | 0.83-1.05 | 71.8% | 0.007 |
| Self report | 2 | 0.84 | 0.64-1.09 | 0.0% | 0.817 |
| Quality assessment |  |  |  |  |  |
| <7 | 8 | 0.67 | 0.53-0.85 | 82.0% | <0.001 |
| ≥7 | 31 | 0.81 | 0.76-0.87 | 91.5% | <0.001 |
| Study location |  |  |  |  |  |
| North America | 19 | 0.80 | 0.71-0.91 | 88.9% | <0.001 |
| Asia | 3 | 0.73 | 0.50-1.07 | 96.1% | <0.001 |
| Europe | 16 | 0.78 | 0.71-0.88 | 91.4% | <0.001 |
| Australia | 1 | 0.53 | 0.40-0.71 | - | - |
| Duration of aspirin use |  |  |  |  |  |
| <5 years | 14 | 0.82 | 0.73-0.92 | 85.3% | <0.001 |
| ≥5 years | 14 | 0.76 | 0.66-0.87 | 78.4% | <0.001 |
| Sex |  |  |  |  |  |
| Men | 7 | 0.81 | 0.71-0.93 | 68.0% | 0.005 |
| Women | 9 | 0.81 | 0.70-0.94 | 76.3% | <0.001 |
| Combined | 29 | 0.78 | 0.72-0.85 | 92.0% | <0.001 |

**Table S5.**Subgroup analysis of relative risk of hepato-biliary cancer

|  | No. of reports | Relative risk | 95%*CI* | *I*^2^ | *P* for heterogeneity |
| --- | --- | --- | --- | --- | --- |
| Study design |  |  |  |  |  |
| Case-control | 4 | 0.51 | 0.24-1.10 | 95.3% | <0.001 |
| Cohort | 3 | 0.80 | 0.65-0.98 | 0.0% | 0.543 |
| Exposure assessment |  |  |  |  |  |
| Prescription | 1 | 0.73 | 0.52-1.02 | - | - |
| Questionnaire | 2 | 0.59 | 0.29-1.19 | 67.3% | 0.080 |
| Records | 2 | 0.34 | 0.30-0.39 | 0.0% | 0.450 |
| Database | 2 | 1.08 | 0.86-1.36 | 0.0% | 0.697 |
| Quality assessment |  |  |  |  |  |
| <7 | 2 | 0.57 | 0.30-1.08 | 59.8% | 0.115 |
| ≥7 | 5 | 0.67 | 0.36-1.24 | 95.4% | <0.001 |
| Study location |  |  |  |  |  |
| North America | 2 | 0.51 | 0.22-1.15 | 95.6% | <0.001 |
| Asia | 2 | 0.57 | 0.30-1.08 | 59.8% | 0.115 |
| Europe | 3 | 0.88 | 0.58-1.35 | 63.1% | 0.067 |
| Sex |  |  |  |  |  |
| Men | 1 | 1.10 | 0.65-1.85 | - | - |
| Women | 1 | 0.70 | 0.24-2.04 | - | - |
| Combined | 6 | 0.59 | 0.35-0.99 | 94.0% | <0.001 |

**Table S6.**Subgroup analysis of relative risk of pancreatic cancer

|  | No. of reports | Relative risk | 95%*CI* | *I*^2^ | *P* for heterogeneity |
| --- | --- | --- | --- | --- | --- |
| Study design |  |  |  |  |  |
| Case-control | 7 | 0.69 | 0.56-0.83 | 63.2% | 0.012 |
| Cohort | 7 | 0.93 | 0.80-1.09 | 57.1% | 0.030 |
| Exposure assessment |  |  |  |  |  |
| Questionnaire | 7 | 0.78 | 0.65-0.94 | 76.6% | <0.001 |
| Interviews | 1 | 0.54 | 0.40-0.73 | - | - |
| Records | 1 | 0.49 | 0.29-0.83 | - | - |
| Database | 2 | 1.10 | 0.91-1.33 | 74.8% | <0.001 |
| Self report | 2 | 0.93 | 0.69-1.26 | 1.1% | 0.315 |
| Quality assessment |  |  |  |  |  |
| <7 | 4 | 0.75 | 0.58-0.97 | 45.2% | 0.140 |
| ≥7 | 10 | 0.81 | 0.67-0.98 | 79.8% | <0.001 |
| Study location |  |  |  |  |  |
| North America | 8 | 0.79 | 0.65-0.96 | 77.8% | <0.001 |
| Asia | 1 | 0.54 | 0.40-0.73 | - | - |
| Europe | 4 | 0.91 | 0.66-1.24 | 62.8% | 0.045 |
| Australia | 1 | 0.81 | 0.61-1.07 | - | - |
| Duration of aspirin use |  |  |  |  |  |
| <5 years | 5 | 0.85 | 0.62-1.16 | 52.2% | 0.079 |
| ≥5 years | 6 | 0.75 | 0.57-0.99 | 48.5% | 0.084 |
| Sex |  |  |  |  |  |
| Men | 3 | 0.90 | 0.75-1.08 | 0.0% | 0.872 |
| Women | 5 | 0.90 | 0.71-1.14 | 61.4% | 0.035 |
| Combined | 9 | 0.74 | 0.59-0.93 | 78.3% | <0.001 |

**Table S7.**Subgroup analysis of relative risk of lung cancer

|  | No. of reports | Relative risk | 95%*CI* | *I*^2^ | *P* for heterogeneity |
| --- | --- | --- | --- | --- | --- |
| Study design |  |  |  |  |  |
| Case-control | 14 | 0.79 | 0.69-0.90 | 70.1% | <0.001 |
| Cohort | 13 | 1.02 | 0.95-1.10 | 55.1% | 0.008 |
| Exposure assessment |  |  |  |  |  |
| Prescription | 1 | 0.95 | 0.93-0.98 | - | - |
| Questionnaire | 13 | 0.91 | 0.82-1.01 | 68.1% | <0.001 |
| Interviews | 9 | 0.84 | 0.67-1.04 | 56.4% | 0.019 |
| Database | 3 | 1.11 | 0.88-1.39 | 75.4% | 0.017 |
| Self report | 1 | 0.68 | 0.49-0.94 | - | - |
| Quality assessment |  |  |  |  |  |
| <7 | 3 | 0.91 | 0.66-1.24 | 74.0% | 0.021 |
| ≥7 | 24 | 0.93 | 0.87-1.00 | 69.2% | <0.001 |
| Study location |  |  |  |  |  |
| North America | 19 | 0.90 | 0.82-0.98 | 63.1% | <0.001 |
| Asia | 3 | 0.71 | 0.39-1.27 | 81.4% | 0.005 |
| Europe | 5 | 1.06 | 0.91-1.24 | 64.4% | 0.024 |
| Duration of aspirin use |  |  |  |  |  |
| <5 years | 8 | 0.94 | 0.81-1.09 | 51.5% | 0.044 |
| ≥5 years | 9 | 0.97 | 0. 28-1.15 | 51.6% | 0.036 |
| Sex |  |  |  |  |  |
| Men | 10 | 0.79 | 0.66-0.95 | 57.3% | 0.012 |
| Women | 15 | 0.86 | 0.79-1.02 | 59.3% | 0.002 |
| Combined | 11 | 1.00 | 0.90-1.10 | 78.0% | <0.001 |

**Table S8.**Subgroup analysis of relative risk of breast cancer

|  | No. of reports | Relative risk | 95%*CI* | *I*^2^ | *P* for heterogeneity |
| --- | --- | --- | --- | --- | --- |
| Study design |  |  |  |  |  |
| Case-control | 13 | 0.84 | 0.77-0.91 | 75.7% | <0.001 |
| Cohort | 20 | 0.96 | 0.91-1.02 | 69.8% | <0.001 |
| Exposure assessment |  |  |  |  |  |
| Prescription | 1 | 0.99 | 0.96-1.02 | - | - |
| Questionnaire | 24 | 0.92 | 0.87-0.98 | 74.7% | <0.001 |
| Interviews | 1 | 0.82 | 0.68-0.98 | - | - |
| Records | 1 | 0.80 | 0.35-1.81 | - | - |
| Database | 4 | 0.94 | 0.85-1.05 | 61.6% | 0.050 |
| Self report | 2 | 0.78 | 0.67-0.90 | 0.0% | 0.472 |
| Quality assessment |  |  |  |  |  |
| <7 | 10 | 0.76 | 0.68-0.85 | 52.3% | 0.026 |
| ≥7 | 23 | 0.97 | 0.94-1.01 | 59.2% | <0.001 |
| Study location |  |  |  |  |  |
| North America | 26 | 0.89 | 0.84-0.94 | 72.6% | <0.001 |
| Asia | 1 | 0.99 | 0.96-1.03 | - | - |
| Europe | 6 | 1.04 | 0.92-1.18 | 65.9% | 0.012 |
| Duration of aspirin use |  |  |  |  |  |
| <5 years | 13 | 1.00 | 0.94-1.06 | 10.3% | 0.342 |
| ≥5 years | 13 | 0.96 | 0.88-1.03 | 38.6% | 0.076 |
| Sex |  |  |  |  |  |
| Women | 33 | 0.92 | 0.88-0.96 | 71.8% | <0.001 |
| Frequency of aspirin use |  |  |  |  |  |
| <7 tablets/week | 3 | 0.89 | 0.57-1.38 | 92.3% | <0.001 |
| ≥7 tablets/week | 3 | 0.84 | 0.64-1.12 | 73.8% | 0.022 |

**Table S9.**Subgroup analysis of relative risk of ovarian cancer

|  | No. of reports | Relative risk | 95%*CI* | *I*^2^ | *P* for heterogeneity |
| --- | --- | --- | --- | --- | --- |
| Study design |  |  |  |  |  |
| Case-control | 13 | 0.85 | 0.77-0.95 | 38.9% | 0.074 |
| Cohort | 8 | 0.92 | 0.83-1.03 | 0.0% | 0.601 |
| Exposure assessment |  |  |  |  |  |
| Prescription | 1 | 0.94 | 0.85-1.04 | - | - |
| Questionnaire | 17 | 0.87 | 0.80-0.96 | 24.9% | 0.167 |
| Interviews | 2 | 0.75 | 0.59-0.95 | 0.0% | 0.793 |
| Database | 1 | 1.10 | 0.75-1.61 | - | - |
| Quality assessment |  |  |  |  |  |
| <7 | 4 | 0.96 | 0.80-1.15 | 30.0% | 0.232 |
| ≥7 | 17 | 0.87 | 0.81-0.94 | 9.9% | 0.338 |
| Study location |  |  |  |  |  |
| North America | 16 | 0.85 | 0.78-0.92 | 0.0% | 0.451 |
| Asia | 1 | 0.87 | 0.52-1.46 | - | - |
| Europe | 5 | 0.93 | 0.85-1.03 | 0.0% | 0.525 |
| Australia | 1 | 1.06 | 0.92-1.23 | - | - |
| Duration of aspirin use |  |  |  |  |  |
| <5 years | 10 | 0.95 | 0.76-1.18 | 54.1% | 0.021 |
| ≥5 years | 11 | 0.77 | 0.63-0.93 | 12.3% | 0.327 |
| Sex |  |  |  |  |  |
| Women | 21 | 0.89 | 0.83-0.95 | 20.8% | 0.192 |

**Table S10.**Subgroup analysis of relative risk of endometrial cancer

|  | No. of reports | Relative risk | 95%*CI* | *I*^2^ | *P* for heterogeneity |
| --- | --- | --- | --- | --- | --- |
| Study design |  |  |  |  |  |
| Case-control | 6 | 0.89 | 0.79-1.01 | 30.1% | 0.210 |
| Cohort | 8 | 0.93 | 0.84-1.04 | 43.3% | 0.090 |
| Exposure assessment |  |  |  |  |  |
| Prescription | 1 | 0.97 | 0.85-1.05 | - | - |
| Questionnaire | 7 | 0.88 | 0.80-0.96 | 0.0% | 0.594 |
| Interviews | 3 | 0.83 | 0.67-1.02 | 20.4% | 0.285 |
| Records | 1 | 1.14 | 0.98-1.33 | - | - |
| Database | 1 | 1.10 | 0.80-1.51 | - | - |
| Self report | 1 | 0.93 | 0.42-2.07 | - | - |
| Quality assessment |  |  |  |  |  |
| <7 | 5 | 1.02 | 0.87-1.20 | 17.7% | 0.302 |
| ≥7 | 9 | 0.90 | 0.83-0.97 | 25.2% | 0.220 |
| Study location |  |  |  |  |  |
| North America | 11 | 0.91 | 0.82-1.01 | 31.6% | 0.147 |
| Asia | 1 | 0.84 | 0.58-1.22 | - | - |
| Europe | 4 | 0.97 | 0.90-1.05 | 0.0% | 0.437 |
| Australia | 1 | 0.78 | 0.63-0.97 | - | - |
| Duration of aspirin use |  |  |  |  |  |
| <5 years | 5 | 0.93 | 0.86-1.00 | 0.0% | 0.686 |
| ≥5 years | 7 | 1.01 | 0.89-1.15 | 19.5% | 0.281 |
| Sex |  |  |  |  |  |
| Women | 14 | 0.92 | 0.85-0.99 | 33.7% | 0.105 |

**Table S11.**Subgroup analysis of relative risk of prostate cancer

|  | No. of reports | Relative risk | 95%*CI* | *I*^2^ | *P* for heterogeneity |
| --- | --- | --- | --- | --- | --- |
| Study design |  |  |  |  |  |
| Case-control | 13 | 0.94 | 0.88-1.00 | 73.2% | <0.001 |
| Cohort | 15 | 0.94 | 0.88-1.01 | 73.3% | <0.001 |
| Exposure assessment |  |  |  |  |  |
| Prescription | 2 | 0.98 | 0.90-1.08 | 93.2% | <0.001 |
| Questionnaire | 12 | 0.96 | 0.92-1.01 | 10.5% | 0.342 |
| Interviews | 1 | 0.66 | 0.51-0.86 | - | - |
| Records | 2 | 0.90 | 0.30-2.71 | 83.8% | 0.013 |
| Database | 9 | 0.93 | 0.85-1.02 | 84.6% | <0.001 |
| Self report | 2 | 0.85 | 0.66-1.08 | 0.0% | 0.377 |
| Quality assessment |  |  |  |  |  |
| <7 | 13 | 0.94 | 0.86-1.02 | 64.6% | 0.001 |
| ≥7 | 15 | 0.94 | 0.89-1.00 | 77.9% | <0.001 |
| Study location |  |  |  |  |  |
| North America | 16 | 0.92 | 0.87-0.97 | 51.8% | 0.018 |
| Asia | 1 | 1.03 | 1.00-1.07 | - | - |
| Europe | 10 | 0.97 | 0.89-1.06 | 81.7% | <0.001 |
| New Zealand | 1 | 0.85 | 0.61-1.19 | - | - |
| Duration of aspirin use |  |  |  |  |  |
| <5 years | 11 | 0.89 | 0.81-0.97 | 77.8% | <0.001 |
| ≥5 years | 9 | 0.90 | 0.81-1.00 | 78.1% | <0.001 |
| Sex |  |  |  |  |  |
| Men | 28 | 0.94 | 0.90-0.99 | 72.2% | <0.001 |

**Table S12.**Subgroup analysis of relative risk of renal cancer

|  | No. of reports | Relative risk | 95%*CI* | *I*^2^ | *P* for heterogeneity |
| --- | --- | --- | --- | --- | --- |
| Study design |  |  |  |  |  |
| Case-control | 6 | 1.13 | 0.94-1.35 | 52.0% | 0.065 |
| Cohort | 9 | 1.02 | 0.87-1.19 | 60.5% | 0.009 |
| Exposure assessment |  |  |  |  |  |
| Questionnaire | 12 | 1.06 | 0.93-1.20 | 56.62% | 0.008 |
| Interviews | 1 | 0.87 | 0.61-1.23 | - | - |
| Database | 1 | 1.40 | 1.13-1.74 | - | - |
| Self report | 1 | 0.60 | 0.29-1.24 | - | - |
| Quality assessment |  |  |  |  |  |
| <7 | 5 | 1.20 | 0.80-1.80 | 74.9% | 0.003 |
| ≥7 | 10 | 1.03 | 0.93-1.13 | 31.1% | 0.160 |
| Study location |  |  |  |  |  |
| North America | 11 | 1.03 | 0.89-1.19 | 64.4% | 0.002 |
| Europe | 2 | 1.20 | 0.85-1.70 | 65.7% | 0.088 |
| Australia | 2 | 1.06 | 0.80-1.41 | 0.0% | 0.557 |
| Duration of aspirin use |  |  |  |  |  |
| <5 years | 7 | 0.92 | 0.78-1.08 | 0.0% | 0.865 |
| ≥5 years | 7 | 1.16 | 0.98-1.37 | 0.0% | 0.839 |
|  |  |  |  |  |  |
|  |  |  |  |  |  |
| Sex |  |  |  |  |  |
| Men | 4 | 1.42 | 0.86-2.34 | 83.1% | 0.001 |
| Women | 5 | 1.02 | 0.85-1.22 | 0.0% | 0.841 |
| Combined | 8 | 1.06 | 0.92-1.22 | 62.1% | 0.010 |

**Table S13.**Subgroup analysis of relative risk of bladder cancer

|  | No. of reports | Relative risk | 95%*CI* | *I*^2^ | *P* for heterogeneity |
| --- | --- | --- | --- | --- | --- |
| Study design |  |  |  |  |  |
| Case-control | 5 | 0.89 | 0.71-1.11 | 76.3% | 0.002 |
| Cohort | 10 | 0.99 | 0.93-1.04 | 0.0% | 0.525 |
| Exposure assessment |  |  |  |  |  |
| Questionnaire | 10 | 0.95 | 0.89-1.00 | 0.0% | 0.780 |
| Interviews | 1 | 0.60 | 0.40-0.90 | - | - |
| Database | 1 | 1.20 | 1.01-1.42 | - | - |
| Self report | 3 | 1.12 | 1.00-1.26 | 0.7% | 0.365 |
| Quality assessment |  |  |  |  |  |
| <7 | 4 | 0.95 | 0.69-1.29 | 66.4% | 0.030 |
| ≥7 | 11 | 0.97 | 0.91-1.04 | 29.3% | 0.166 |
| Study location |  |  |  |  |  |
| North America | 12 | 0.97 | 0.90-1.04 | 30.8% | 0.145 |
| Europe | 3 | 0.98 | 0.76-1.27 | 74.7% | 0.019 |
| Duration of aspirin use |  |  |  |  |  |
| <5 years | 5 | 0.97 | 0.74-1.26 | 50.9% | 0.086 |
| ≥5 years | 5 | 1.10 | 0.95-1.28 | 0.0% | 0.411 |
| Sex |  |  |  |  |  |
| Men | 4 | 1.04 | 0.94-1.14 | 28.8% | 0.240 |
| Women | 4 | 1.01 | 0.88-1.16 | 0.0% | 0.743 |
| Combined | 8 | 0.94 | 0.83-1.08 | 58.8% | 0.018 |

**Table S14.**Subgroup analysis of relative risk of brain tumor

|  | No. of reports | Relative risk | 95%*CI* | *I*^2^ | *P* for heterogeneity |
| --- | --- | --- | --- | --- | --- |
| Study design |  |  |  |  |  |
| Case-control | 4 | 0.88 | 0.77-1.01 | 0.0% | 0.593 |
| Cohort | 3 | 1.26 | 1.02-1.55 | 81.5% | 0.005 |
| Exposure assessment |  |  |  |  |  |
| Prescription | 1 | 0.90 | 0.77-1.05 | - | - |
| Questionnaire | 3 | 0.91 | 0.61-1.36 | 71.9% | 0.028 |
| Interviews | 1 | 1.07 | 0.63-1.79 | - | - |
| Database | 2 | 1.33 | 0.86-2.07 | 90.3% | 0.001 |
| Quality assessment |  |  |  |  |  |
| <7 | 1 | 1.07 | 0.63-1.79 | - | - |
| ≥7 | 6 | 1.07 | 0.89-1.28 | 75.3% | <0.001 |
| Study location |  |  |  |  |  |
| North America | 4 | 0.96 | 0.71-1.29 | 58.0% | 0.068 |
| Europe | 3 | 1.15 | 0.89-1.49 | 88.3% | <0.001 |
| Duration of aspirin use |  |  |  |  |  |
| <5 years | 3 | 0.82 | 0.68-0.98 | 29.6% | 0.242 |
| ≥5 years | 3 | 0.65 | 0.43-0.97 | 68.6% | 0.276 |
| Sex |  |  |  |  |  |
| Men | 2 | 1.22 | 0.88-1.68 | 60.5% | 0.111 |
| Women | 3 | 1.33 | 0.84-2.09 | 90.7% | <0.001 |
| Combined | 4 | 0.95 | 0.76-1.19 | 39.4% | 0.175 |

**Table S15.**Subgroup analysis of relative risk of head and neck cancers

|  | No. of reports | Relative risk | 95%*CI* | *I*^2^ | *P* for heterogeneity |
| --- | --- | --- | --- | --- | --- |
| Study design |  |  |  |  |  |
| Case-control | 7 | 0.87 | 0.70-1.09 | 64.9% | 0.009 |
| Cohort | 3 | 0.99 | 0.73-1.33 | 79.4% | 0.008 |
| Exposure assessment |  |  |  |  |  |
| Questionnaire | 6 | 0.79 | 0.66-0.94 | 32.2% | 0.195 |
| Interviews | 1 | 1.00 | 0.59-1.68 | - | - |
| Database | 3 | 1.09 | 0.92-1.29 | 58.4% | 0.090 |
| Quality assessment |  |  |  |  |  |
| <7 | 4 | 0.62 | 0.32-1.22 | 55.4% | <0.081 |
| ≥7 | 6 | 0.95 | 0.81-1.11 | 73.4% | 0.002 |
| Study location |  |  |  |  |  |
| North America | 3 | 0.76 | 0.64-0.92 | 15.6% | 0.306 |
| Europe | 7 | 1.01 | 0.87-1.18 | 51.3% | 0.055 |
| Sex |  |  |  |  |  |
| Men | 1 | 1.26 | 0.93-1.72 | - | - |
| Women | 1 | 1.64 | 1.05-2.58 | - | - |
| Combined | 9 | 0.88 | 0.75-1.03 | 61.8% | 0.007 |

**Table S16.**Subgroup analysis of relative risk of skin cancer

|  | No. of reports | Relative risk | 95%*CI* | *I*^2^ | *P* for heterogeneity |
| --- | --- | --- | --- | --- | --- |
| Study design |  |  |  |  |  |
| Case-control | 6 | 0.93 | 0.86-1.02 | 61.7% | 0.023 |
| Cohort | 8 | 0.98 | 0.93-1.03 | 45.6% | 0.076 |
| Exposure assessment |  |  |  |  |  |
| Questionnaire | 7 | 0.98 | 0.92-1.04 | 59.2% | 0.023 |
| Interviews | 2 | 0.75 | 0.64-0.89 | 0.0% | 0.6632 |
| Records | 1 | 0.94 | 0.90-0.99 | - | - |
| Database | 2 | 1.00 | 0.90-1.12 | 38.0% | 0.204 |
| Self report | 2 | 0.97 | 0.62-1.53 | 0.0% | 0.479 |
| Quality assessment |  |  |  |  |  |
| <7 | 3 | 0.98 | 0.93-1.02 | 0.0% | 0.778 |
| ≥7 | 11 | 0.96 | 0.91-1.01 | 63.2% | 0.002 |
| Study location |  |  |  |  |  |
| North America | 11 | 0.95 | 0.89-1.02 | 59.1% | 0.006 |
| Europe | 3 | 0.97 | 0.92-1.01 | 33.6% | 0.222 |
| Duration of aspirin use |  |  |  |  |  |
| <5 years | 7 | 1.00 | 0.91-1.10 | 20.7% | 0.271 |
| ≥5 years | 9 | 0.98 | 0.87-1.10 | 63.8% | 0.005 |
| Sex |  |  |  |  |  |
| Men | 1 | 0.73 | 0.50-1.07 | - | - |
| Women | 4 | 0.92 | 0.82-1.02 | 78.0% | 0.003 |
| Combined | 10 | 0.97 | 0.93-1.02 | 27.0% | 0.195 |

**Table S17.**Subgroup analysis of relative risk of lymphoma

|  | No. of reports | Relative risk | 95%*CI* | *I*^2^ | *P* for heterogeneity |
| --- | --- | --- | --- | --- | --- |
| Study design |  |  |  |  |  |
| Case-control | 6 | 0.88 | 0.75-1.03 | 41.5% | 0.129 |
| Cohort | 10 | 1.02 | 0.90-1.16 | 50.1% | 0.035 |
| Exposure assessment |  |  |  |  |  |
| Questionnaire | 8 | 1.00 | 0.87-1.15 | 60.1% | 0.014 |
| Interviews | 3 | 0.75 | 0.55-1.03 | 52.1% | 0.124 |
| Records | 1 | 1.20 | 0.78-1.85 | - | - |
| Database | 3 | 1.04 | 0.89-1.21 | 0.0% | 0.565 |
| Self report | 1 | 0.67 | 0.34-1.32 | - | - |
| Quality assessment |  |  |  |  |  |
| <7 | 4 | 0.93 | 0.67-1.28 | 78.0% | 0.001 |
| ≥7 | 11 | 0.98 | 0.90-1.07 | 13.6% | 0.314 |
| Study location |  |  |  |  |  |
| North America | 12 | 0.93 | 0.82-1.06 | 58.5% | 0.005 |
| Europe | 4 | 1.05 | 0.92-1.22 | 0.0% | 0.677 |
| Duration of aspirin use |  |  |  |  |  |
| <5 years | 6 | 0.96 | 0.86-1.07 | 0.0% | 0.479 |
| ≥5 years | 10 | 0.99 | 0.88-1.10 | 30.3% | 0.166 |
| Sex |  |  |  |  |  |
| Men | 6 | 0.92 | 0.74-1.15 | 74.5% | 0.001 |
| Women | 8 | 0.88 | 0.78-0.98 | 0.0% | 0.448 |
| Combined | 7 | 1.09 | 0.95-1.24 | 26.4% | 0.227 |

**Table S18.**Subgroup analysis of relative risk of leukemia

|  | No. of reports | Relative risk | 95%*CI* | *I*^2^ | *P* for heterogeneity |
| --- | --- | --- | --- | --- | --- |
| Study design |  |  |  |  |  |
| Case-control | 3 | 1.17 | 0.61-2.26 | 77.9% | 0.011 |
| Cohort | 4 | 0.80 | 0.51-1.26 | 88.7% | <0.001 |
| Exposure assessment |  |  |  |  |  |
| Questionnaire | 5 | 0.89 | 0.57-1.38 | 84.8% | <0.001 |
| Database | 1 | 1.30 | 0.03-1.64 | - | - |
| Self report | 1 | 0.67 | 0.34-1.32 | - | - |
| Quality assessment |  |  |  |  |  |
| <7 | 3 | 1.15 | 0.53-2.53 | 79.2% | 0.008 |
| ≥7 | 4 | 0.83 | 0.54-1.27 | 88.4% | <0.001 |
| Study location |  |  |  |  |  |
| North America | 6 | 0.85 | 0.58-1.26 | 81.3% | <0.001 |
| Europe | 1 | 1.30 | 1.03-1.64 | - | - |
| Duration of aspirin use |  |  |  |  |  |
| <5 years | 2 | 1.05 | 0.84-1.31 | 0.0% | 0.920 |
| ≥5 years | 3 | 0.84 | 0.69-1.02 | 0.0% | 0.467 |
| Sex |  |  |  |  |  |
| Men | 1 | 1.20 | 0.87-1.65 | - | - |
| Women | 4 | 0.78 | 0.47-1.30 | 86.9% | <0.001 |
| Combined | 4 | 1.04 | 0.70-1.53 | 69.1% | 0.021 |
